# Identification and Characterization of Neuropeptides and Their G Protein-Coupled Receptors (GPCRs) in the Cowpea Aphid *Aphis craccivora*

**DOI:** 10.3389/fendo.2020.00640

**Published:** 2020-09-17

**Authors:** Xiao Li, Long Du, Xiao-Jing Jiang, Qian Ju, Chun-Juan Qu, Ming-Jing Qu, Tong-Xian Liu

**Affiliations:** ^1^Department of Plant Protection, Shandong Peanut Research Institute, Qingdao, China; ^2^State Key Laboratory of Crop Stress Biology for Arid Areas and Key Laboratory of Integrated Pest Management on the Loess Plateau of Ministry of Agriculture, Northwest A&F University, Yangling, China

**Keywords:** *Aphis craccivora*, neuropeptides, G protein-coupled receptors (GPCRs), expression profiling, transcriptome

## Abstract

Neuropeptides are the most abundant and diverse signal molecules in insects. They act as neurohormones and neuromodulators to regulate the physiology and behavior of insects. The majority of neuropeptides initiate downstream signaling pathways through binding to G protein-coupled receptors (GPCRs) on the cell surface. In this study, RNA-seq technology and bioinformatics were used to search for genes encoding neuropeptides and their GPCRs in the cowpea aphid *Aphis craccivora*. And the expression of these genes at different developmental stages of *A. craccivora* was analyzed by quantitative real-time PCR (qRT-PCR). A total of 40 candidate genes encoding neuropeptide precursors were identified from the transcriptome data, which is roughly equivalent to the number of neuropeptide genes that have been reported in other insects. On this basis, software analysis combined with homologous prediction estimated that there could be more than 60 mature neuropeptides with biological activity. In addition, 46 neuropeptide GPCRs were obtained, of which 40 belong to rhodopsin-like receptors (A-family GPCRs), including 21 families of neuropeptide receptors and 7 orphan receptors, and 6 belong to secretin-like receptors (B-family GPCRs), including receptors for diuretic hormone 31, diuretic hormone 44 and pigment-dispersing factor (PDF). Compared with holometabolous insects such as *Drosophila melanogaster*, the coding genes for sulfakinin, corazonin, arginine vasopressin-like peptide (AVLP), and trissin and the corresponding receptors were not found in *A. craccivora*. It is speculated that *A. craccivora* likely lacks the above neuropeptide signaling pathways, which is consistent with *Acyrthosiphon pisum* and that the loss of these pathways may be a common feature of aphids. In addition, expression profiling revealed neuropeptide genes and their GPCR genes that are differentially expressed at different developmental stages and in different wing morphs. This study will help to deepen our understanding of the neuropeptide signaling systems in aphids, thus laying the foundation for the development of new methods for aphid control targeting these signaling systems.

## Introduction

Insect neuropeptides are trace polypeptides produced by neurosecretory glands or neurosecretory cells in insects (1). They are the most abundant and diverse signal molecules and play important regulatory roles in the physiology and behavior in insects such as the growth, development, metabolism, reproduction, and feeding of insects (2). Most of the known neuropeptides are oligopeptides or small protein molecules composed of several to tens of amino acids. Biologically active mature neuropeptides are formed by complex post-translational processing of neuropeptide precursors. Neuropeptides act as signal molecules to exert their physiological functions as neuromodulators or neurohormones by binding to corresponding membrane receptors. Except for a few families of neuropeptides, such as prothoracicotropic hormone (PTTH), eclosion hormone (EH), and neuropeptide-like precursor 1 (NPLP1), the majority of neuropeptide receptors belong to the G protein-coupled receptors (GPCRs) superfamily (3). In recent years, insect GPCRs have been proposed to be used as potential targets to explore new insecticides or behavioral regulators, opening up new ways for the safe control of pests (4) By targeting neuropeptide GPCRs, future insecticides or behavioral regulators can prevent or over-stimulate the normal activity of these proteins, producing a lethal or deleterious effect on pests. Many species of aphids are important agricultural and forestry pests. At the same time, aphids are also model organisms for studying phenotypic plasticity, insect-plant interactions, insect-plant-virus interactions and endosymbionts and thus have attracted much attention from researchers (5).

In recent years, the continuous innovation of next-generation sequencing technology and bioinformatics technology has greatly promoted the prediction and identification of insect neuropeptides and their receptors (6–9). However, only *Acyrthosiphon pisum* has so far been comprehensively investigated for neuropeptides and their receptors in aphids (10–12). *Aphis craccivora* is a worldwide insect pest that causes harm to many crops such as cowpea, peanut and pea (13, 14). In addition to directly sucking plant sap, it can also cause fungal diseases and acts as a vector for various plant viruses such as cowpea mosaic virus (15) and peanut stripe virus (16). At present, *A. craccivora* is mainly controlled by chemical insecticides, resulting in increasingly severe insecticide resistance and ecological problems (17). So it is imperative to develop alternative new control strategy and identification of neuropeptides and their receptors of *A. craccivora* will be the first step toward achieving this goal.

In the present study, based on transcriptome sequencing and bioinformatics analysis, we screened the genes encoding neuropeptide precursors and their GPCRs in *A. craccivora*. We also conducted structural analysis and mature peptide prediction for the identified neuropeptide precursors and classified the neuropeptide receptors into subclasses based on phylogenetic analysis. The expression profiles of these neuropeptides and their GPCRs at different developmental stages were determined by quantitative real-time PCR (qRT-PCR). These identified gene sequences provide the basis for further investigation of the function of *A. craccivora* neuropeptides and their GPCRs.

## Materials and Methods

### Insect Rearing, Sample Collection, and RNA Extraction

*Aphis craccivora* was collected from peanut fields in Laixi, Shandong Province, China, in 2015. Then the collected aphids were reared for many generations with *Vicia faba* seedlings in our laboratory under the photoperiod of 14 L: 10 D, at 22 ± 1°C. For transcriptome sequencing in the next step, the heads of 1,000 wingless parthenogenetic adults were quickly removed into phosphate-buffered saline (PBS) buffer and immediately frozen with liquid nitrogen. To find as many target genes as possible, we also collected 50–60 whole-body aphids from each instar (including wingless and winged morphs) as a mixed sample to generate the second transcriptome. Total RNA extraction was performed from the above two samples using RNAiso Plus reagent (Takara, Dalian, China) following the manufacturer's instructions.

### Transcriptome Sequencing, Assembly and Annotation

We used 2 μg total RNA to construct each library with the NEBNext® Ultra™ RNA Library Prep Kit for Illumina® (Illumina, San Diego, CA, USA). Paired-end sequencing was performed using an Illumina HiSeq^TM^ 2,500 by Gene *Denovo* Biotechnology Co. (Guangzhou, China). After the raw sequencing reads were filtered to remove adaptors, low-quality sequences and reads with *N* content <5%, *de novo* assembly were performed using Trinity software with the default parameters (18). Finally, the annotation of unigene sequences were performed using a Blastx search against the NCBI non-redundant protein sequence (Nr), A manually annotated and reviewed protein sequence database (Swiss-Prot), Kyoto Encyclopedia of Genes and Genomes (KEGG), and Clusters of Orthologous Groups (COG) databases with a cut-off *E*-value of 10^−5^.

### Identification of Neuropeptides and Their Putative GPCRs in *A. craccivora*

tBlastn was utilized to search for unigenes encoding neuropeptides (including protein hormones) and their putative GPCRs from *A. craccivora* transcriptome using the homologs from *A. pisum* (10–12), *D. melanogaster* (19–21), *Anopheles gambiae* (22, 23), *Apis mellifera* (24, 25), *Bombyx mori* (26, 27), *Tribolium castaneum* (28, 29), *Nilaparvata lugens* (6), *Rhodnius prolixus* (30), and *Chilo suppressalis* (31) as the query sequences. The open reading frames (ORFs) of the candidate unigenes were predicted using the online tool ORF finder (http://www.ncbi.nlm.nih.gov/gorf/gorf.html) and manually corrected based on homology to the published insect neuropeptides and their GPCRs. And then the encoded protein sequences were aligned to the Nr database using NCBI's Blastp program (https://blast.ncbi.nlm.nih.gov/Blast.cgi).

### Structure and Domain Analysis

For the neuropeptide precursors, SignalP 4.1 (http://www.cbs.dtu.dk/services/SignalP/) was employed to predict the signal peptides (32). The splice sites were predicted using the Known Motif and Insect Models methods of NeuroPred (http://stagbeetle.animal.uiuc.edu/cgi-bin/neuropred.py) (33) and were corrected based on the processing procedures of known insect neuropeptide precursors. Sulfinator (https://web.expasy.org/sulfinator/) was used to identify the sulfation of tyrosine residues (34). Cyclization of N-terminal glutamine/glutamic acid residue and amidation of the C-terminal glycine residue were predicted based on homology to the reported invertebrate neuropeptides. Multiple alignments of amino acid sequences were performed using Clustal X 2.0 and the results were visualized using GeneDoc software.

For the neuropeptide GPCRs, trans-membrane domains (TMDs) were predicted by TMHMM 2.0 (http://www.cbs.dtu.dk/services/TMHMM.NCBI) CD-Search tool (http://www.ncbi.nlm.nih.gov/Structure/cdd/wrpsb.cgi) was employed to search the conserved domain database (CDD) for the domain (35, 36).

### Reverse Transcription PCR (RT-PCR) Amplification of Neuropeptide GPCR Genes

Two unigene sequences in the transcriptome data were likely partial fragments from the same gene. RT-PCR was used to amplify and obtain longer sequences. Upstream and downstream primers were designed for each of the fragments using Primer Premier 5.0 software (Table S1). The cDNA template was synthesized using a PrimeScript™ II 1st Strand cDNA Synthesis Kit (Takara, Dalian, China) The reaction procedure was as follows: pre-denaturation at 94°C for 3 min; 37 cycles of denaturation at 94°C for 30 s, annealing at 56°C for 30 s, and elongation at 72°C for 1 min; and a final elongation at 72°C for 5 min. The amplication products were sent to Invitrogen (Shanghai, China) for sequencing. The sequencing results were compared with the corresponding unigene sequences using Blastn to determine sequence identity.

### Phylogenetic Analysis

Neuropeptide GPCRs of *A. craccivora* were compared with the reported homologs from *A. pisum, N. lugens, D. melanogaster, B. mori*, and *T. castaneum*. Amino acid sequences with a length shorter than 150 aa were removed from the data set for phylogenetic analysis. Multiple alignments were carried out using MAFFT (37). The maximum-likelihood (ML) phylogenetic trees were constructed using the FastTree software (version 2.1.7) under the default settings (38). Local support values were calculated based on the Shimodaira-Hasegawa (SH) test implemented within FastTree. Metabotropic glutamate receptor (mGluR) of *D. melanogaster* (encoded by CG11144) served as the outgroup for the phylogenetic tree of A-family GPCRs, and 5-hydroxytryptamine receptor (5-HTR) of *D. melanogaster* (encoded by CG1056) served as the outgroup for the phylogenetic tree of B-family GPCRs. The phylogenetic trees were finally edited and displayed using FigTree 1.4.3 (http://tree.bio.ed.ac.uk/software/figtree).

### Expression Profiling of Neuropeptides and Their GPCRs at Different Developmental Stages

To determine the transcriptional expression levels of neuropeptides and their GPCRs at different life stages of *A. craccivora*, 20–60 aphids from each instar were collected separately for qRT-PCR analysis. From the third instar, we were able to distinguish winged from wingless aphids by wing buds of the thorax. For this reason, winged and wingless morphs of the aphids were collected separately from the 3rd, the 4th instar nymphs and adults. After total RNA was extracted as described above, cDNA was synthesized by reverse transcribing 1 μg of total RNA using a PrimeScript™ RT reagent Kit with gDNA Eraser (Perfect Real Time) (Takara, Dalian, China) according to the manufacturer's instructions. The gene-specific primers for qRT-PCR were designed using the NCBI online primer design tool (https://www.ncbi.nlm.nih.gov/tools/primer-blast/) (Table S2). We select 40S ribosomal protein S8 gene (*RPS8*) as the reference gene to normalize the expression of the target genes (39).

The qRT-PCR reactions were run with SYBR Premix Ex Taq II (Tli RNaseH Plus) (Takara, Dalian, China) on an iQ™5 Multicolor Real-Time PCR Detection System (Bio-Rad Hercules, CA, USA). The qRT-PCR program was set as follows: 95°C for 3 min; 40 cycles of 95°C for 10 s and 58°C for 30 s; and followed by a melting cycle (from 60 to 95°C with an increase of 0.5°C per cycle). The experiment included 3 biological replicates and 3 technical replicates. The relative expression level of the target genes was calculated using the 2^Δ*ΔCt*^ method (40). Statistical analysis was performed using SPSS 20.0 (Systat Software Inc., London, UK). Statistical significance was evaluated using one-way ANOVA followed by the Tukey HSD test at the 0.05 level.

## Results

### Transcriptome Sequencing, Assembly, and Annotation

We sequenced the sample from the heads of *A. craccivora* wingless parthenogenetic adults and 56,634,657 clean reads (8.4 Gbp) were obtained. For the mixed sample, 70,096,246 clean reads (10.3 Gbp) were obtained. After assembly, 60,113 unigenes (N50 = 1,169) were obtained with an average length of 703 bp and a GC content of 38.08%. Among all the unigenes, a total of 45,150 were annotated in the Nr, Swissprot, KOG, and KEGG databases (Figure S1A), and the top 3 species with the most matches in Nr database were *A. pisum, Diuraphis noxia*, and *Diachasma alloeum* (Figure S1B). The transcriptome raw data have been submitted to the Sequence Read Archive (SRA) database at NCBI under the accession number SRP189414.

### Identification of Neuropeptide Precursors in *A. craccivora*

Based on the homology search and Nr-annotation, a total of 40 genes encoding neuropeptide precursors were identified, involving 32 neuropeptide families (Table 1; Supplementary Material 1) and covering most insect neuropeptide families (Table S3). However, a small number of family members were missing, including CNMamide (CNMa), corazonin (Crz), elevenin, neuroparsin, pigment-dispersing factor (PDF), RYamide (RYa), sulfakinin (SK), sex peptide (SP), trissin, arginine vasopressin-like peptide (AVLP), and adipokinetic hormone/corazonin-related peptide (ACP). Of the 40 neuropeptide genes obtained, 36 had intact ORFs, and the remaining four non-full-length sequences include three insulin-like peptide (ILP)-encoding genes and 1 PTTH -encoding gene (Table 1). The majority of *A. craccivora* neuropeptide precursors showed more than 90% similarity with their homologous sequences in other aphids, whereas a few had low similarity with homologous sequences in related species, such as proctolin (Proc), leucokinin (LK), and diuretic hormone 44 (DH44). Besides, orthologs of some *A. pisum* neuropeptides were not identified from *A. craccivora* transcriptome, including ion transport peptides (ITPs) and ILPs. However, the transcript encoding ITP-like was found in the *A. craccivora* transcriptome. Ten different genes coding for ILPs have been found in *A. pisum* genome but only six of these genes in *A. craccivora*, i.e., genes encoding ILP1, ILP4, ILP5, ILP7, ILP8, and ILP10. By contrast, the gene coding for NPLP3 possesses orthologs in *A. craccivora* but not in *A. pisum*.

**Table 1:** Putative neuropeptides identified from *A. craccivora*.

**Neuropeptide (abbreviation)**	**Unigene ID**	**ORF** **(aa)**	**SP** **(aa)**	**Homology search with known protein (Blastp)**			
				**Accession no**.	**Species**	***E*-value**	**Identity (%)**
Adipokinetic hormone (AKH)	Unigene0027719	70	19	ALH44119.1	*Aphis gossypii*	4.00E-45	100
Allatostatin A (AstA)	Unigene0032333	265	20	XP_025204927.1	*Melanaphis sacchari*	2.00E-165	91
Allatostatin B (AstB)	Unigene0029473	219	22	XP_026805639.1	*Rhopalosiphum maidis*	2.00E-153	97
Allatostatin C (AstC)	Unigene0013997	124	29	XP_022182906.1	*Myzus persicae*	3.00E-69	90
Allatostatin CC (AstCC)	Unigene0053841	136	20	AWT50588.1	*Diaphorina citri*	9.00E-30	65
Allatotropin (AT)	Unigene0043447	131	26	XP_025207325.1	*Melanaphis sacchari*	3.00E-85	95
Bursicon alpha subunit (Burα)	Unigene0009955	160	23	XP_022171710.1	*Myzus persicae*	3.00E-113	98
Bursicon beta subunit (Burβ)	Unigene0017703	137	21	XP_025201526.1	*Melanaphis sacchari*	1.00E-92	97
Capability/CAP2b (CAPA)	Unigene0012156	149	23	XP_015366927.1	*Diuraphis noxia*	5.00E-94	89
Crustacean cardioactive peptide (CCAP)	Unigene0044769	135	23	XP_025193376.1	*Melanaphis sacchari*	7.00E-93	98
CCHamide 1 (CCHa1)	Unigene0034430	141	23	XP_022173158.1	*Myzus persicae*	3.00E-92	90
CCHamide 2 (CCHa2)	Unigene0056057	119	–	XP_022183663.1	*Myzus persicae*	9E-78	92
Diuretic hormone 31/Calcitonin-like peptide (DH31)	Unigene0016990	123	30	XP_008189818.1	*Acyrthosiphon pisum*	6.00E-82	98
Diuretic hormone 44/CRF-like diuretic hormone (DH44)	Unigene0030947	125	19	PRD30623.1	*Nephila clavipes*	2.00E-11	53
Eclosion hormone 1 (EH1)	c17200_g1	83	26	XP_016662241.1	*Acyrthosiphon pisum*	9.00E-44	85
Eclosion hormone 2 (EH2)	c17200_g2	83	26	XP_016662241.1	*Acyrthosiphon pisum*	8.00E-42	81
Eclosion hormone 3 (EH3)	Unigene0032823	83	26	XP_016662241.1	*Acyrthosiphon pisum*	4.00E-43	83
Ecdysis-triggering hormone (ETH)	Unigene0053622	180	20	NP_001156684.1	*Acyrthosiphon pisum*	2.00E-122	92
FMRFamide (FMRFa)	Unigene0034006	151	13	XP_003240304.1	*Acyrthosiphon pisum*	2.00E-92	93
Glycoprotein hormone alpha 2 (GPA2)	Unigene0030917	129	28	XP_025205714.1	*Melanaphis sacchari*	1.00E-90	98
Glycoprotein hormone beta 5 (GPB5)	Unigene0022160	142	21	XP_022165863.1	*Myzus persicae*	9.00E-94	91
Insulin-like peptide 1 (ILP1)	c15478_g1	125	21	XP_025196181.1	*Melanaphis sacchari*	4.00E-46	86
Insulin-like peptide 4 (ILP4)	c25074_g2	122	21	XP_026819766.1	*Rhopalosiphum maidis*	3.00E-70	93
Insulin-like peptide 5 (ILP5)	c20960_g2	94	19	XP_026821759.1	*Rhopalosiphum maidis*	7.00E-09	32
Insulin-like peptide 7 (ILP7)	c10146_g1	41^*^	–	XP_025203322.1	*Melanaphis sacchari*	9.00E-21	95
Insulin-like peptide 8 (ILP8)	c15326_g1	112^*^	–	XP_025191957.1	*Melanaphis sacchari*	1.00E-34	54
Insulin-like peptide 10 (ILP10)	c15424_g1	138	22	XP_025191957.1	*Melanaphis sacchari*	1E-46	59
Ion transport peptide-like (ITPL)	Unigene0031227	152	29	XP_015370790.1	*Diuraphis noxia*	2.00E-107	98
Leucokinin (LK)	Unigene0012286	203	23	BAV78814.1	*Diaphorina citri*	3.00E-07	33
Myosuppressin (MS)	Unigene0026279	108	23	XP_025197199.1	*Melanaphis sacchari*	7.00E-72	98
Neuropeptide F (NPF)	Unigene0058658	156	19	AEE01348.1	*Aphis gossypii*	1.00E-120	97
Neuropeptide-like precursor 1 (NPLP1)	Unigene0027013	444	19	XP_022201186.1	*Nilaparvata lugens*	e-16	27
Neuropeptide-like precursor 3 (NPLP3)	Unigene0044545	90	16	XP_011304822.1	*Fopius arisanus*	2.00E-07	63
Orcokinin (OK)	Unigene0031395	144	22	XP_001947462.1	*Acyrthosiphon pisum*	2.00E-80	75
Pheromone biosynthesis activating neuropeptide /Pyrokinin 2 (PBAN/PK2)	Unigene0026434	304	19	XP_003245653.1	*Acyrthosiphon pisum*	7.00E-121	78
Proctolin (Proc)	Unigene0027851	102	16	AEX08669.1	*Rhodnius prolixus*	0.003	44
Prothoracicotropic hormone (PTTH)	Unigene0022074	207^*^	–	ARM65501.1	*Acyrthosiphon pisum*	7.00E-93	74
SIFamide (SIFa)	Unigene0052320	75	25	XP_025195729.1	*Melanaphis sacchari*	7.00E-49	97
Short neuropeptide F (sNPF)	Unigene0022605	96	20	XP_022169760.1	*Myzus persicae*	6.00E-63	98
Tachykinins (TK)	Unigene0033673	188	21	XP_025199482.1	*Melanaphis sacchari*	9.00E-99	87

SP, signal peptide;

* *not full length; –, no signal peptide*.

### Mature Neuropeptide Prediction

**Figure 1:**
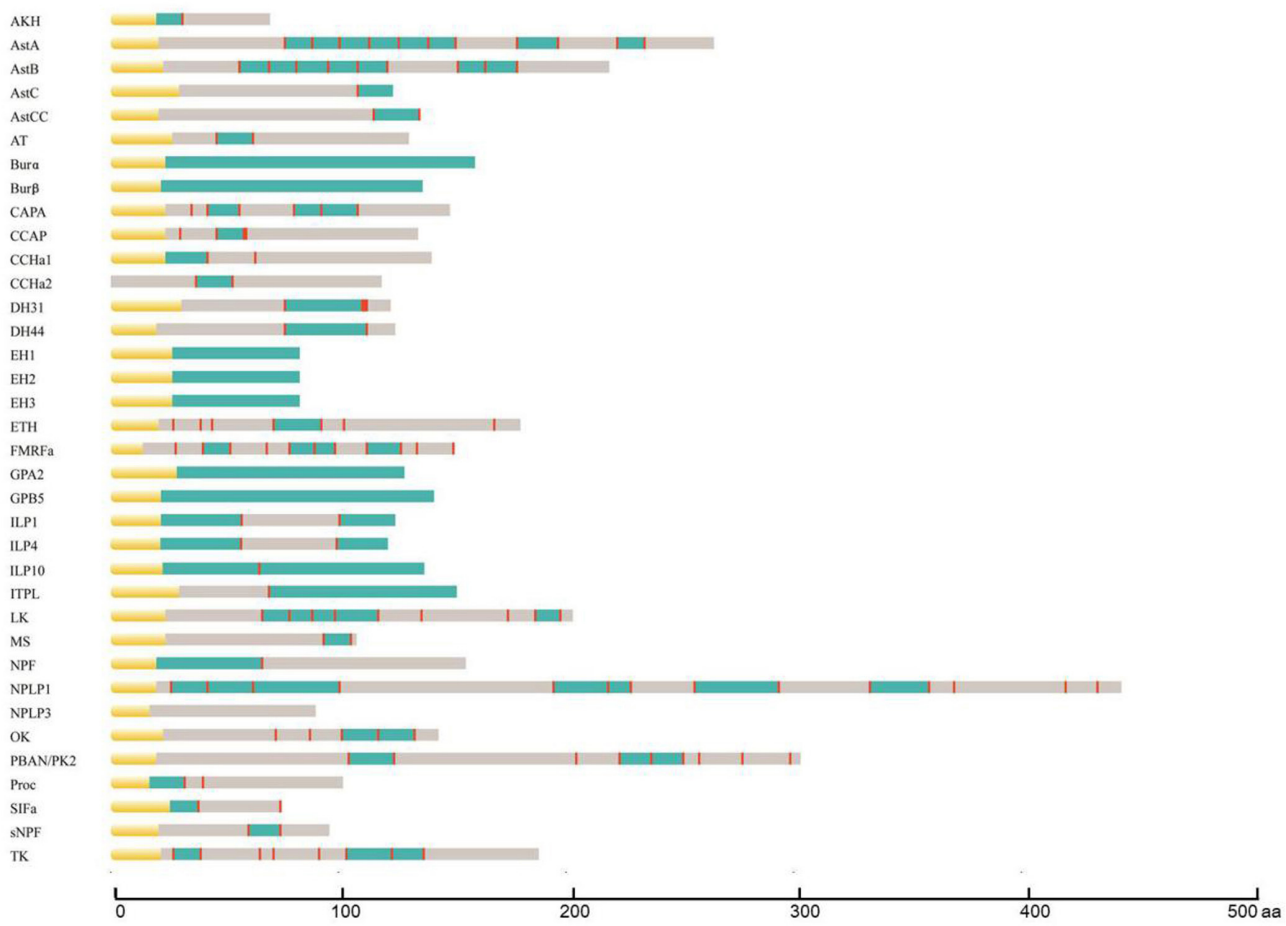
Schematic diagrams showing the organization of *A. craccivora* neuropeptide precursors. Yellow, signal peptide; blue, bioactive peptides; red, cleavage sites. Scale bars at bottom show length in amino acids (aa). Four incomplete sequences were not included; cleavage sites were not predicted in the NPLP3 precursor.

During the process of neuropeptide production, a larger inactive neuropeptide precursor is first synthesized. One or several bioactive mature peptides are then formed through a series of enzymatic digestions and post-translational modifications, which are critical to ensure the biological activity and stability of mature neuropeptides (41). The signal peptide cleavage site, sulfation site, amidation site, and other modification sites of *A. craccivora* neuropeptide precursors were predicted, and the prediction results were revised based on the homology and conserved motif of the known invertebrate neuropeptides. A total of 164 mature peptides (including protein hormones) were predicted from 40 neuropeptide precursors in *A. craccivora*. Among them, more than 60 mature peptides could be biologically active. Meanwhile, a total of 52 mature peptides were predicted to be amidated at the C-terminus, and 11 sites of 6 mature peptides were predicted as potential sites of sulfation (Figure 1; Supplementary Material 2).

Most *A. craccivora* neuropeptides have highly conserved characteristic motifs within the same family. For example, the predicted 8 mature allatostatin A (AstA) peptides contain the C-terminal signature sequence of -Y/FXFGL/Iamide; adipokinetic hormone (AKH) has the sequence motif characterized by pQX_6_Wamide. Some mature neuropeptide sequences are identical in different aphids, such as short neuropeptide F (sNPF) and SIFamide (SIFa) of *A. craccivora, A. pisum*, and *Myzus persicae*. Even the sequences of mature peptides of DH31 and DH44 that are up to tens of amino acids are identical in *A. craccivora* and *A. pisum* (Supplementary Material 2).

The mature neuropeptides of *A. craccivora* also exhibit some structural variability, which is mainly reflected by the expansion and truncation of the polypeptide sequences. The former mainly involves Proc, CCHamide (CCHa), and AstB, and the latter occurs in SIFa. The predicted Proc of *A. craccivora* is identical to that of *M. persicae* with an N-terminal extension compared to the pentapeptide proctolin (RYLPT) of most other insects (42); the effect of this expansion on Proc biological activity remains unknown. The N-terminus of *A. craccivora* CCHa1 has an addition of 4 amino acids (KQGA), and the N-terminus of CCHa2 has an additional Gly. There are two AstB peptides with a sequence motif of -WX_7_Wamide in *A. craccivora*, which has an extension of one amino acid residue in the middle of the polypeptide compared to the classical motif. This subtype of AstB has also been found in *A. pisum, M. persicae, A. gossypii, N. lugens*, and *R. prolixus* (6, 43). Therefore, it is speculated that -WX_7_Wamide may be a special AstB subtype that is prevalent in hemipteran insects. The predicted *A. craccivora* SIFa sequence (FRKPPFNGSIFamide) is consistent with that of *A. pisum* and *M. persicae*, but lacks an amino acid residue at the N-terminus compared to the classical motif.

In addition, variability is also reflected in the mutation of amino acid residues. The *A. craccivora capability* (*CAPA*) gene encodes a CAPA-pvk (periviscerokinin) peptide and a CAPA-pk (pyrokinin) peptide with the conserved characteristic motif, -PRVamide, and -PRLamide, respectively. This gene also encodes another polypeptide, EGLIAFPRIamide, which is identical to the CAPA-2 peptide of *A. pisum* and *M. persicae* (42, 44). This subtype of CAPA with -PRIamide is less common in insects. In addition to aphids, it is found only in a few insects such as *T. castaneum, A. mellifera*, and *Camponotus floridanus* (29, 45, 46).

### Neuropeptide GPCRs in *A. craccivora*

**Figure 2:**
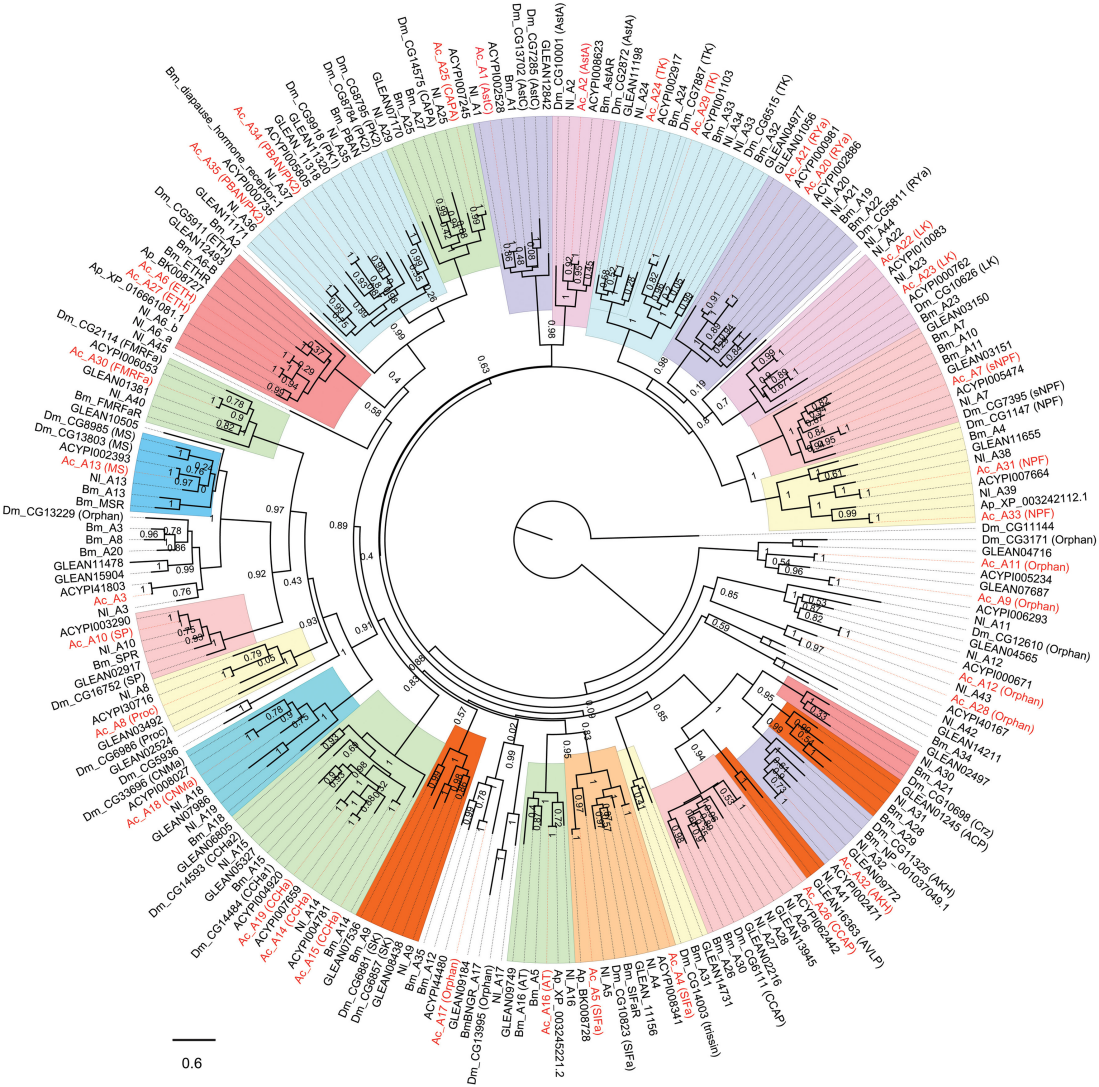
Phylogenetic tree of A-family neuropeptide GPCRs (LGRs are excluded). The maximum-likelihood tree was constructed using FastTree software with local support values calculated based on the Shimodaira-Hasegawa (SH) test. The tree is rooted by the metabotropic glutamate receptor CG11144 from *D. melanogaster*. Ac, *A. craccivora*; Dm, *D. melanogaster*; Ap, *A. pisum*; Bm, *B. mori*; Nl, *N. lugens*; ACYPIxxxxx, sequences from *A. pisum*; GLEANxxxxx, sequences from *T. castaneum*.

**Figure 3:**
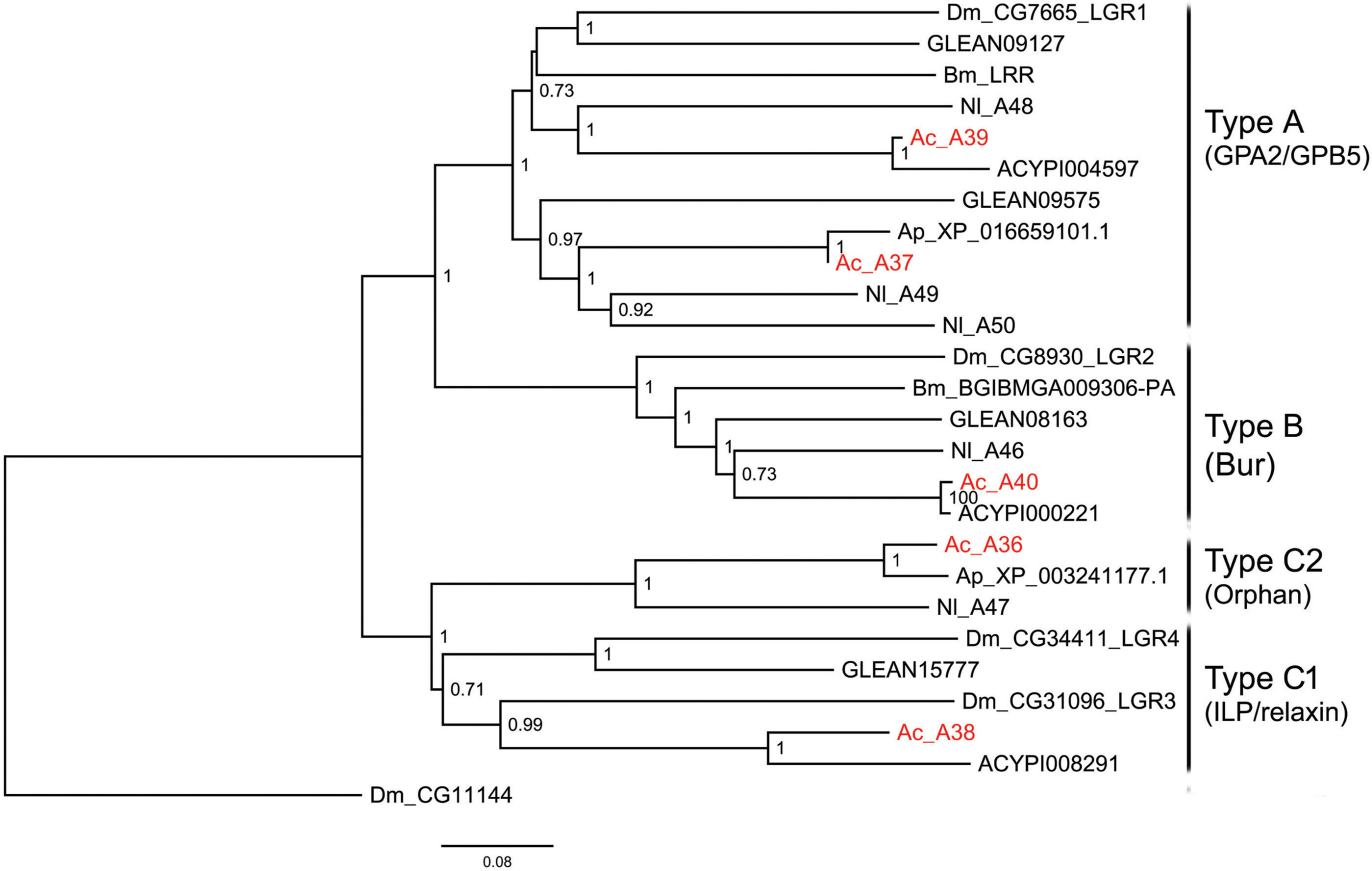
Phylogenetic tree of LGRs. The maximum-likelihood tree was constructed using FastTree software with local support values calculated based on the Shimodaira-Hasegawa (SH) test. The tree is rooted by the serotonin receptor CG1056 from *D. melanogaster*. Ac, *A. craccivora*; Dm, *D. melanogaster*; Ap, *A. pisum*; Bm, *B. mori*; Nl, *N. lugens*; ACYPIxxxxx, sequences from *A. pisum*; GLEANxxxxx, sequences from *T. castaneum*.

**Figure 4:**
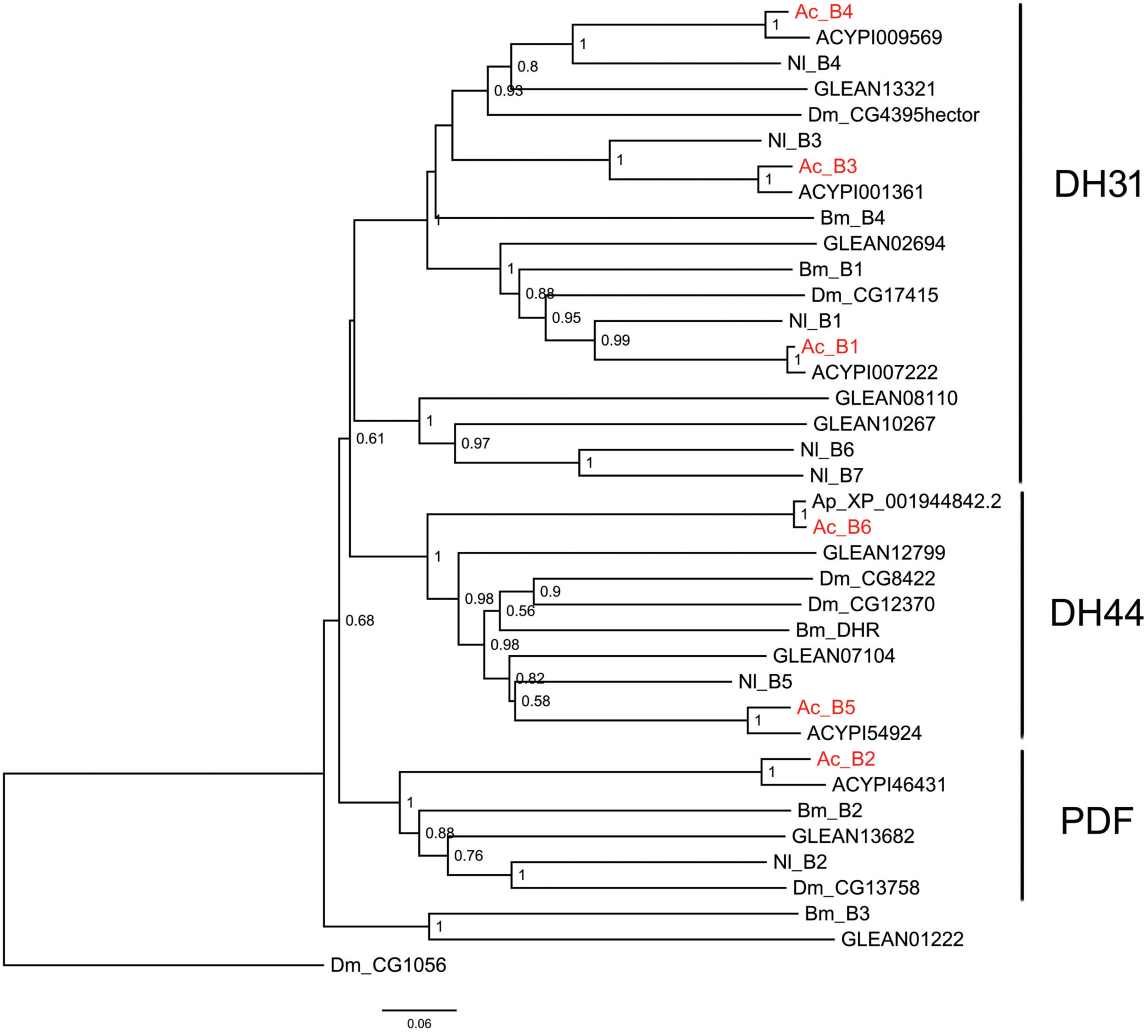
Phylogenetic tree of B-family neuropeptide GPCRs. The maximum-likelihood tree was constructed using FastTree software with local support values calculated based on the Shimodaira-Hasegawa (SH) test. The tree is rooted by the serotonin receptor CG1056 from *D. melanogaster*. Ac, *A. craccivora*; Dm, *D. melanogaster*; Ap, *A. pisum*; Bm, *B. mori*; Nl, *N. lugens*; ACYPIxxxxx, sequences from *A. pisum*; and GLEANxxxxx, sequences from *T. castaneum*.

The insect neuropeptide GPCRs belong to rhodopsin-like receptors (A-family GPCRs) or secretin-like receptors (B-family GPCRs). A total of 46 putative neuropeptide GPCRs were identified from the *A. craccivora* transcriptome data, among which 34 contained complete ORFs (Table 2; Supplementary Material 3). The encoded theoretical proteins had a length of 154–1184 amino acids. Forty of 46 GPCRs belong to the A-family (represented by 7tm_1 or PF0001 in the Pfam database), and six belong to the B-family (represented by 7tm_2 or PF0002 in the Pfam database) (Table 2). Phylogenetic analysis revealed that all *A. craccivora* neuropeptide GPCRs had unique orthologous proteins in *A. pisum* (Figures 2–4).

**Table 2:** Putative neuropeptide GPCRs identified in *A. craccivora*.

**GPCR name**	**Likely ligand**	**GPCR class**	**Unigene ID**	**ORF (aa)**	**TM**	**Homology search with known protein (Blastp)**			
						**Acc.no**	**Species**	***E*-value**	**Identity (%)**
Ac_A1	AstC	Class A	c25397_g1	443	7	XP_026810241.1	*Rhopalosiphum maidis*	0	96
Ac_A2	AstA	Class A	c19014_g1	387	7	XP_026805021.1	*Rhopalosiphum maidis*	0	95
Ac_A3	Orphan	Class A	c23632_g1	396	7	XP_026813221.1	*Rhopalosiphum maidis*	0	93
Ac_A4	SIFa	Class A	c27545_g1	441	7	XP_026810900.1	*Rhopalosiphum maidis*	0	99
Ac_A5	SIFa	Class A	c24582_g1	387	7^*^	XP_025202590.1	*Melanaphis sacchari*	0	97
Ac_A6	ETH	Class A	c25283_g1	383	7^*^	XP_022182490.1	*Myzus persicae*	2.00E-124	97
Ac_A7	sNPF	Class A	unigene0031155	456	7	XP_025199183.1	*Melanaphis sacchari*	0	98
Ac_A8	Proc	Class A	unigene0019560	406	6^*^	XP_015380053.1	*Diuraphis noxia*	0	94
Ac_A9	Orphan	Class A	c13254_g1	356	7^*^	XP_026812348.1	*Rhopalosiphum maidis*	0	97
Ac_A10	SP	Class A	c21429_g1	393	7	XP_027845906.1	*Aphis gossypii*	0	98
Ac_A11	Orphan	Class A	c44582_g1	154	4^*^	XP_026812253.1	*Rhopalosiphum maidis*	2e-106	99
Ac_A12	Orphan	Class A	c18209_g1	400	7	XP_025204296.1	*Melanaphis sacchari*	0	97
Ac_A13	MS	Class A	c26478_g1	421	7	XP_026808214.1	*Rhopalosiphum maidis*	0	94
Ac_A14	CCHa	Class A	c12924_g1	314	5^*^	XP_025190494.1	*Melanaphis sacchari*	0	92
Ac_A15	CCHa	Class A	c24117_g1	378	7	XP_026822094.1	*Rhopalosiphum maidis*	0	96
Ac_A16	AT	Class A	c27080_g1	437	7	XP_026823352.1	*Rhopalosiphum maidis*	0	94
Ac_A17	Orphan	Class A	c24908_g1	453	7	XP_026818291.1	*Rhopalosiphum maidis*	0	98
Ac_A18	CNMa	Class A	c25234_g1	365	7	XP_026809022.1	*Rhopalosiphum maidis*	0	97
Ac_A19	CCHa	Class A	unigene0035849	407	7	XP_026822545.1	*Rhopalosiphum maidis*	0	97
Ac_A20	RYa	Class A	unigene0006642	326	7	XP_026812520.1	*Rhopalosiphum maidis*	0	94
Ac_A21	RYa	Class A	c21731_g2	410	7	XP_026810999.1	*Rhopalosiphum maidis*	0	95
Ac_A22	LK	Class A	unigene0038062	393	7	XP_022173987.1	*Myzus persicae*	0	97
Ac_A23	LK	Class A	c18353_g1	438	7	XP_026804282.1	*Rhopalosiphum maidis*	0	95
Ac_A24	TK	Class A	c21909_g1	454	7	XP_026810922.1	*Rhopalosiphum maidis*	3.00E-170	98
Ac_A25	CAPA	Class A	c19757_g1	487	7	XP_026822835.1	*Rhopalosiphum maidis*	0	94
Ac_A26	CCAP	Class A	c23525_g2	335	7	XP_025205664.1	*Melanaphis sacchari*	0	99
Ac_A27	ETH	Class A	unigene0021484	248	3^*^	XP_026808728.1	*Rhopalosiphum maidis*	2.00E-169	96
Ac_A28	Orphan	Class A	c25921_g1	421	7	XP_026810563.1	*Rhopalosiphum maidis*	0	99
Ac_A29	TK	Class A	c23903_g1	477	7	XP_022172034.1	*Myzus persicae*	0	93
Ac_A30	FMRFa	Class A	c20833_g1	498	7	XP_026804884.1	*Rhopalosiphum maidis*	0	87
Ac_A31	NPF	Class A	c25336_g1	391	7	XP_026807386.1	*Rhopalosiphum maidis*	0	97
Ac_A32	AKH	Class A	c23253_g1	421	7	XP_003245941.1	*Acyrthosiphon pisum*	0	96
Ac_A33	NPF	Class A	c20484_g1	476	7^*^	XP_022177908.1	*Myzus persicae*	0	93
Ac_A34	PBAN/PK2	Class A	c23906_g1	472	7	XP_026804890.1	*Rhopalosiphum maidis*	0	95
Ac_A35	PBAN/PK2	Class A	c23752_g1	460	7	XP_026815970.1	*Rhopalosiphum maidis*	0	89
Ac_A36	Orphan	Class A	c27060_g1	1087	7^*^	XP_027853902.1	*Aphis gossypii*	0	98
Ac_A37	GPA2/GPB5	Class A	c26277_g1	476	7^*^	XP_026820801.1	*Rhopalosiphum maidis*	0	97
Ac_A38	ILP/relaxin	Class A	c17409_g1	318	6^*^	XP_026819406.1	*Rhopalosiphum maidis*	4.00E-174	79
Ac_A39	GPA2/GPB5	Class A	c22388_g1	380	7^*^	XP_025205838.1	*Melanaphis sacchari*	0	98
Ac_A40	Bur	Class A	c25922_g1	1184	7	XP_001948776.2	*Acyrthosiphon pisum*	0	98
Ac_B1	DH31	Class B	c24053_g1	409	7	XP_026809008.1	*Rhopalosiphum maidis*	0	98
Ac_B2	PDF	Class B	c17700_g1	427	7	XP_027844571.1	*Aphis gossypii*	0	99
Ac_B3	DH31	Class B	c25414_g1	474	7	XP_008184836.1	*Acyrthosiphon pisum*	0	96
Ac_B4	DH31	Class B	c24744_g1	401	7	XP_025206446.1	*Melanaphis sacchari*	0	92
Ac_B5	DH44	Class B	c27642_g1	400	7	XP_026815200.1	*Rhopalosiphum maidis*	0	95
Ac_B6	DH44	Class B	unigene0036280	473	7	XP_027838173.1	*Aphis gossypii*	0	99

* *not full length*.

In insects, neuropeptide (including protein hormones) receptors account for more than two-thirds of A-family GPCRs (12). According to the binding ligands, A-family neuropeptide GPCRs of *A. craccivora* were divided into 21 groups: receptors for RYa, LK, tachykinin (TK), SIFa, ecdysis-triggering hormone (ETH), CAPA, PK, Proc, FMRFamide (FMRFa), SP, mysupression (MS), AstA/AstC, CCHa, CNMa, allatotropin (AT), sNPF, NPF, crustacean cardioactive peptide (CCAP), AKH, Bursicon (Bur), and GPA2/GPB5 (Figures 2, 3). In addition to these GPCRs, 7 orphan receptors, A3, A9, A11, A12, A17, A28, and A36, were clustered into six orphan receptor branches (Figures 2, 3). No orthologous genes encoding the receptors for SK, Crz, AVLP, and trissin were found in *A. craccivora*. A recent study found that elevenin in *N. lugens* regulates body color via the receptor Nl_42 (47), but no ortholog of this receptor was found in *A. craccivora* (Figure 2).

Leucine-rich repeat-containing GPCRs (LGRs) are a special class of neuropeptide/protein hormone receptors in A-family GPCRs. They act as receptors for glycoprotein hormone or insulin/relaxin-related proteins and play important regulatory roles in development and reproduction of insects (48, 49). According to the structure characteristics, LGRs are divided into type A, type B, and type C. The main differences among the 3 types are the number of leucine-rich repeats (LRRs), the presence or absence of a low density lipoprotein receptor-like cysteine-rich (LDLa) motif, and type-specific hinge region (48, 49). In this study, a total of 5 LGRs (A36-A40) were identified from *A. craccivora* (Figure 3). Among them, only A40 has a complete ORF. CDD analysis indicated that A40 has 15 LRR motifs and no LDLa motif, showing typical structural characteristics of a type B LGR. A36 has an N-terminus containing 7 LDLa repeats and 8 LRR motifs, consistent with the typical characteristics of a type C LGR. The presence of conserved domains was not detected in other LGRs due to incomplete sequences. Phylogenetic analysis revealed that *A. craccivora* A37 and A39 were clustered into the branch of GPA2/GPB5 receptor. A40 is the ortholog of *D. melanogaster* LGR2, which has been confirmed to be a receptor for Bursicon and is also the first deorphanized LGR in invertebrates (50, 51). It has been confirmed that dILP8 is a ligand for LGR3 in *D. melanogaster* (52), and the A38 receptor of *A. craccivora* is clustered into this branch. Thus, it is speculated that A38 may be an insulin-like receptor. In addition, there is an orphan receptor, A36, clustered into the C2 LGR subfamily (Figure 3).

B-family GPCRs are a small class of receptors that differ structurally and functionally from other families. Their remarkable structural feature is a long N-terminal domain. The B-family can be further subdivided into three subfamilies: subfamily B1, B2, and B3 (53). Neuropeptide GPCRs belong to the B1 subfamily and contain three types of neuropeptide/protein hormone receptors: calcitonin receptor (also known as DH31 receptor), corticotropin releasing factor (CRF)-like diuretic hormone receptor (also known as DH44 receptor), and PDF receptor (19). All these three types of receptors were identified in *A. craccivora* and involved a total of 6 sequences (B1–B6) (Figure 4). Phylogenetic analysis revealed that *A. craccivora* B1, B3, and B4 were clustered in the branch of DH31 receptor; B5 and B6 were clustered in the branch of DH44 receptor; and B2 was clustered in the branch of PDF receptor (Figure 4).

### Expression Profiles of Neuropeptides and Their GPCRs at Different Developmental Stages of *A. craccivora*

**Figure 5:**
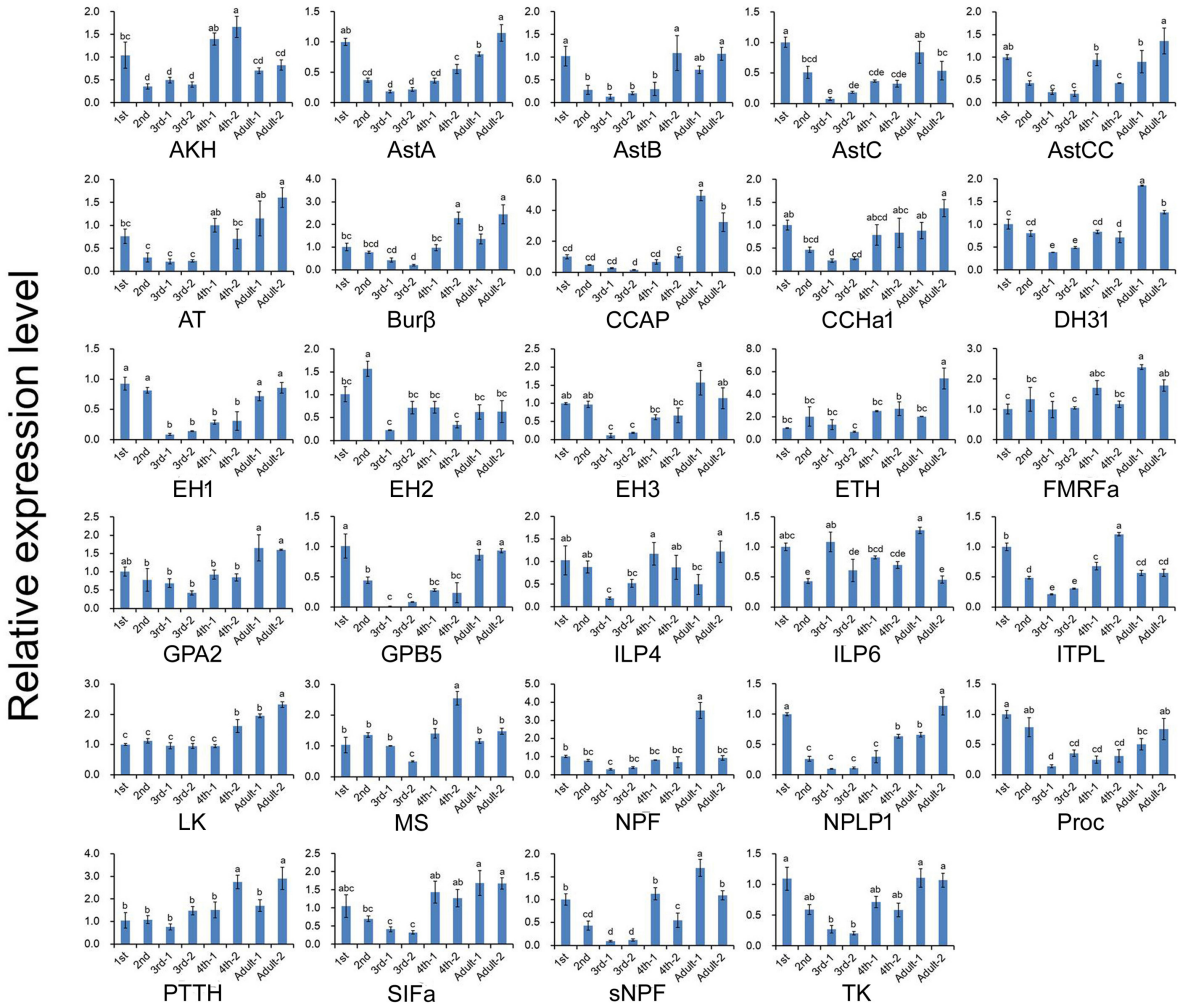
Relative expression levels of neuropeptide-encoding genes at different developmental stages of *A. craccivora*. The transcript level was measured via qRT-PCR and normalized against *RPS8*. Data are presented as the mean ± SD. The data were statistically analyzed by one-way ANOVA followed by Tukey's HSD test. Different lowercase letters indicate significant differences at the 0.05 level. “−1” indicates wingless aphids; “−2” indicates winged aphids.

**Figure 6:**
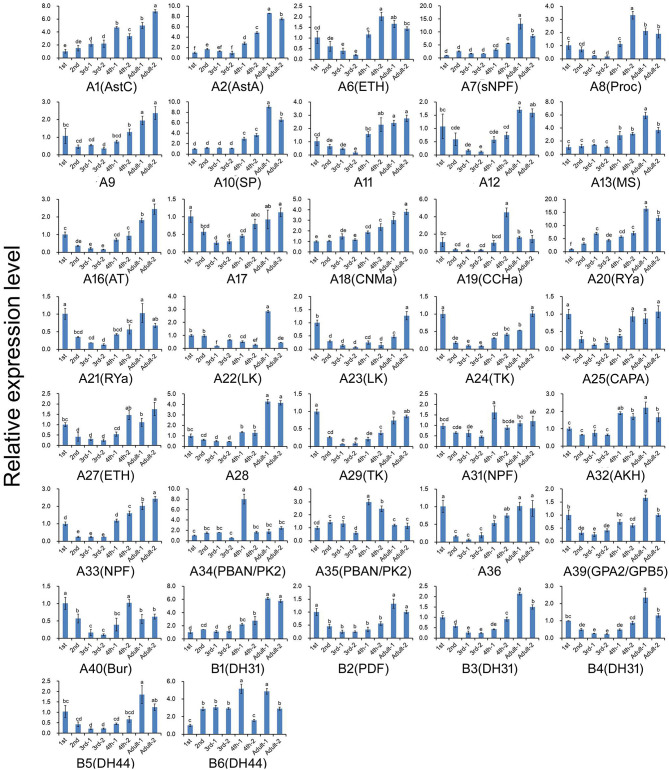
Relative expression levels of the genes encoding G protein-coupled receptors for the neuropeptides at different developmental stages of *A. craccivora*. The transcript level was measured via qRT-PCR and normalized against *RPS8*. Data are presented as the mean ± SD. The data were statistically analyzed by one-way ANOVA followed by Tukey's HSD test. Different lowercase letters indicate significant differences at the 0.05 level. Neuropeptide receptors A1–A40 are abbreviated as A1–A40, respectively; Neuropeptide receptors B1–B6 are abbreviated as B1–B6, respectively. And the receptors are marked with their predicted neuropeptide ligand partners in brackets; orphan receptors are not marked. “−1” indicates wingless aphids; “−2” indicates winged aphids.

The expression profiles of *A. craccivora* neuropeptides and their GPCRs at different developmental stages were determined by qRT-PCR. For most of the measured neuropeptide-encoding genes, the expression abundance showed a trend of decreasing first and then increasing during development, i.e., the expression level was significantly downregulated at the 2^nd^ and 3^rd^ instar stages (Figure 5). For the neuropeptide GPCR genes, most were differentially expressed at different developmental stages and in different wing morphs, and moreover, most were more abundant in the adult stage than in other stages (Figure 6). The two TK receptors, A24 and A29, had basically the same developmental-stage expression pattern, while the expression patterns of the two members of NPF receptor family, A31 (NPFR ortholog) and A33 (NPY2R ortholog), were different (Figure 6). Overall, the gene expression patterns of most neuropeptide-encoding genes and their presumed GPCR-encoding genes were not consistent (Figures 5, 6).

## Discussion

Based on RNA-seq technology, we identified a large number of genes encoding neuropeptides and their GPCRs from *A. craccivora* in this study. The types and numbers of these genes are basically similar to those found in other insects, indicating that RNA-seq can be used as a powerful tool for the excavation of these genes from insects in the absence of whole-genome data. For some neuropeptide signaling systems, *A. craccivora* seems to lack both receptor and neuropeptide precursor (SK, Crz, AVLP, trissin, and elevenin). For others, the neuropeptide was not found or is lacking, but its presumed GPCR is present (e.g., PDF, RYa, CNMa, etc.). Why were they not found? There may be three main reasons. Firstly, the sequenced samples probably did not cover all stages of the *A. craccivora* life cycle, and some genes may not have been expressed in these measured samples; in other cases, some genes are expressed at low abundance in certain tissues or at certain developmental stages and the transcripts would be further diluted in the mixed sample. Secondly, due to the lack of strong sequence conservation among some neuropeptide precursors or neuropeptide GPCRs, their clear orthologs could not be found in *A. craccivora* based on homology searches. Thirdly, the neuropeptides may truly be absent in this species, a phenomenon that is common in insects. For example, sex peptide was reported only in the *Drosophila* genus; while neuroparsin is missing in *D. melanogaste*r and other *Drosophila* species (54). Compared to *D. melanogaster, B. mori*, and *T. castaneum*, we found neither the genes coding for SK, Crz, AVLP, trissin, and elevenin in *A. craccivora* nor the genes coding for their receptors. If both neuropeptide and its receptor are truly absent, it is more likely that this signaling system is lacking as a whole based on neuropeptide-receptor co-evolution in this aphid species. Likewise, the former four signaling systems are not found in *A. pisum* (10, 12). These absent pathways are mainly involved in feeding, ecdysis and osmotic homeostasis in other insects (55–58), suggesting the absense is the result of constant adaptation to the environment during evolution of aphids. SK, Crz, and AVLP neuropeptide signaling systems were found in another hemipteran insect, *N. lugens*, but the trissin signaling system was absent in this species (6). For the neuropeptide GPCRs, two receptor genes for LK, SIFa and NPF have been identified from *A. craccivora, A. pisum*, and *N. lugens* (Figure 2), suggesting that each of these receptor genes duplicated in the evolution. These 3 types of receptors are mainly functionally involved in water balance, sexual behavior, and feeding behavior (59–62). However, we cannot conclude from these findings that the replication of these genes is a common feature of hemipterans, because they are a highly diverse group of insects.

A total of 40 genes enconding neuropeptides were identified from *A. craccivora*. Compared with another aphid species *A. pisum* (10), ITP and several ILPs were not detected from *A. craccivora*. ITP is a member of the crustacean hyperglycaemic hormone/ITP (CHH/ITP) superfamily, which stimulates the ileum to transport Cl^−^ ion and may act as an antidiuretic hormone in insects (63, 64). In addition to ITP peptide, the *ITP* gene also encodes an alternatively spliced variant without C-terminal amidation (known as ITP-like or ITPL) (64). Only the transcript encoding ITPL was found in the *A. craccivora* transcriptome, whereas the ITP-encoding transcript was not. Generally, invertebrate genomes contain multiple genes encoding ILPs including 10 ILP precursor-encoding genes in the *A. pisum* genome (10), while only 6 of this family were found in *A. craccivora*. In contrast to ITPs and ILPs, an NPLP3 ortholog is present in *A. craccivora* but not in *A. pisum*. There are 4 NPLP-encoding genes, *NPLP1-4*, in *D. melanogaster* (65); *NPLP2* and *NPLP3* were found in *A. mellifera* (25). However, only one NPLP-encoding gene, *NPLP1*, was found in *A. pisum* (10). We found *NPLP1* and *NPLP3* in *A. craccivora*, which is consistent with that previously reported in a hemipteran insect, *Diaphorina citri* (7), while *NPLP1, NPLP3*, and *NPLP4* are present in another hemipteran insect, *N. lugens* (6). Interestingly, no genes encoding PTTH orthologs were originally identified in hemipteran insects such as *A. pisum* and *R. prolixus*, probably due to insufficient conservation of PTTH sequences (6, 10). However, we used the sequences of PTTH precursors reported in other insects as a query to conduct a homology search and found a unigene in the *A. craccivora*. Moreover, a homologous sequence (ARM65502.1) was also found in *A. pisum* by the Blast program. PTTH is a polypeptide hormone that stimulates prothoracic glands to synthesize and release ecdysone and it was first cloned and characterized in *B. mori* (66). Mature *B. mori* PTTH is a homodimer. Each monomer contains 7 conserved Cys residues, of which 6 Cys form 3 internal disulfide bonds and the 7th Cys forms a disulfide bond between two monomers (66, 67). PTTH was later identified in several lepidopterans (68) and *D. melanogaster* (69). Compared with other neuropeptides, the sequence similarity of PTTH between different insects was relatively lower. However, the position of Cys residues and the folding structure of dimers were highly conservative (69–72). Tanaka et al. (6) used *B. mori* PTTH as a query to search for the analog in the *N. lugens* transcriptome and finally found a unigene whose theoretical protein has conserved structural features of PTTH. This is the first PTTH-like gene identified from hemimetabolous insects. We also found a unigene from the *A. craccivora* transcriptome that lacks the 5′-end of the ORF and may encode a partial sequence of the PTTH precursor. Sequence alignment revealed that the PTTH-like precursor of *A. craccivora* lacks the Cys residue at position 4 (Figure S2). The PTTH ortholog in *A. pisum* (ARM65502.1) also lacks the Cys residue at this position. Notably, all the 7 Cys residues of PTTH monomer are involved in the formation of disulfide bonds, which is required for PTTH to achieve biological activity (70). The effect of the absence of the fourth Cys residue on the formation of the PTTH advanced structure and whether it affects its normal biological function in aphids deserves further investigation.

We also carried out the expression profiling of genes encoding neuropeptides and their receptors in different developmental stages. Unfortunately, the low expression level of some measured genes reduced the accuracy of their qRT-PCR data. Thus, such genes were not included in the results (Figures 5, 6). The expression and functions of insect neuropeptides may change with development (73). In the present study, the tested neuropeptide genes also showed different levels of expression during development of *A. craccivora*. However, whether the differential temporal expressions of these genes have a biological role on development needs further investigation. It is worth noting that NPF was expressed in *A. craccivora* wingless adults at a significantly higher level compared with that in winged adults. NPF is homologous to the vertebrate neuropeptide Y (NPY) and its functional roles are well-known in regulation of feeding behavior in a wide range of insects (60). This neuropeptide was also suggested to play a positive role in the regulation of food intake in *A. pisum* using RNAi knockdown (74). In view of this, whether the differential expression of NPF between morphs has an impact on feeding behavior of different wing-morph adults or not, worths further research.

Nutritional conditions are key environmental factors that affect the growth and development of insects. The ILPs play a main role in systematically regulating the growth of the body in response to nutritional conditions (75). A prominent feature of insect ILPs is that multiple genes encode different ILPs. For example, 8 ILPs have been identified in *D. melanogaster* (76, 77), while 39 ILPs have been identified in *B. mori* (78). The synthesis and secretion of different ILPs in the same insect species exhibits different temporal and spatial pattern, and their functions are also different (79). In *A. pisum, ILP5* has attracted the attention of researchers due to its abundant expression. It is speculated that this gene may be involved in the rapid development of aphids (10). It has also been reported that *ILP5* is involved in embryonic development during wing differentiation in *A. pisum* (79). From the RPKM values, the expression abundance of *ILP5* gene in *A. craccivora* is much higher than other ILP-encoding genes, which is consistent with that reported in *A. pisum* (80). The *ILP5* transcript was detected from both the head as well as the mixed whole-body samples of *A. craccivora*. Besides, it was abundantly expressed in the head of wingless adult aphids. *A. pisum ILP5* gene shows the similar expression pattern: it is expressed systemically in *A. pisum* adults and third instar nymphs with the highest expression level in the head of wingless adults (including the antennae) (81). This expression characteristic of *ILP5* in aphids suggests the importance of its biological function and deserves further in-depth investigation.

In this study, the sequence similarity between neuropeptide precursors is very high among different species of aphids. Several neuropeptide genes, such as *sNPF, SIFa, AstC, AstCC*, and *Proc*, even encode identical mature active peptides in different species of aphids. For neuropeptide GPCRs, besides high sequence similarity, all of the identified *A. craccivora* neuropeptide GPCRs have a one-to-one orthologous relationship with the homologs of *A. pisum*. This result indicates that the aphid neuropeptide GPCRs are also highly conserved reflected by their types and numbers. In summary, the neuropeptide signaling systems in aphids are highly conserved and provide potential insecticide targets for the control of aphids. In addition, most neuropeptide signalings are conserved in both holometabolous and hemimetabolous insects, suggesting that they play key roles in the physiological processes of insects (3). Some neuropeptide signaling systems are lost in specific species, possibly due to functional redundancy that occurs during adaptation and evolution or other neuropeptide signalings taking over their functions (3).

## Data Availability Statement

The datasets generated for this study can be found in online repositories. The names of the repository/repositories and accession number(s) can be found in the article/Supplementary Material.

## Author Contributions

T-XL and M-JQ supervised the whole project. XL and LD performed the experiments and wrote the manuscript with equal contributions. X-JJ, QJ, and C-JQ provided technical support. All authors contributed to the article and approved the submitted version.

## Conflict of Interest

The authors declare that the research was conducted in the absence of any commercial or financial relationships that could be construed as a potential conflict of interest.
