# Supplementary material for: Identification and Characterization of Neuropeptides and Their G Protein-Coupled Receptors (GPCRs) in the Cowpea Aphid Aphis craccivora
Source: Front Endocrinol (Lausanne). 2020 Sep 17;11:640. doi: 10.3389/fendo.2020.00640 (PMC7527416; doi:10.3389/fendo.2020.00640)
Supplement: Supplementary file 1 [file Data_Sheet_1.docx]

Supplementary material 1 Nucleic acid sequences of neuropeptide genes in *A. craccivora*

>AKH Unigene0027719

TAATACAGAAAGCAATGCATTTTGAATAAATATTAAACAATAAAACTAAAATATTCTCTA

TAAAACATATCGAAAATAAACAAAAATGTGGTTGATGGATAATAATAAATTTGGTGGATA

TATATAATTATACATAATAATTGAAAACGGTAGAACTTTATATTATTATTATTATTATTA

TTACCATCTTAATAATGTGACTAAAAGTAAAAATGTAACACTGTAACTGACGAAGTATCA

GAGTATAAGTCCCGTTGTTAATGATCTATGGCTGGCGTGTGGTATAGTGGCGTTGGTCAT

CTCCTGCAATCGTGACTGTTCAAGACGTTATGGACATTCGTTCACACATTGCGATCCGCT

GCGCTTCGTTCTGCACCAGCTTGTATATGTAGATGAGCGTGTCCATGGACTTGCACTCGT

CCGATGCGGGAGCGTTCCTCTTGCCCTGGCCCCACGTCGGCGTGAAGTTCACTTGACCGA

CGACGATGCACGCGCACAACATGAACACAGCCAACAACAACAGGGACCGCATGATGGCGA

TGGACGTGGAGACGGAGTATAATATTATTATTGTTATTATTTTTTTTTACCGGTGACCGT

CGAGCTACTCGCAGACAGTCGTACAATAATATATAATATTATATTATATTATATGCGGCT

AGCTTTACTTTGACACGAACGGTGGTTTAGCGGGTGATTCAGAGTTGATATTTGACTGTT

TGCGGCGTTTAATGTTTTGTGCGGTTGCCGGACGTTTCCGTATGTATATTATTATATATT

ATAGATAGTAATATATTGTGCTGGTATTCAGTTGTGCACGGGTGGTTGCAGCGTGCGTAA

ATGACGTGATGTAACCGCGAAA

>AstA Unigene0032333

AACAAATCATAAATAAATCGTCGGTCTGTTTTCTTTATTATTGTAATTTATTTCTTAGTT

TTTTTTTTTTTTTAATAATACGTTTCTGTGAGTTGAGTGTAGATACTGAATTTTATAAAA

CATTATGCTTGACTGTAAATGTTTCATAGGTGGAGATTTAATCGAATATTAGTGGGGGTT

TTTTTTTTTTGAATAAATCATTCGTTAAAAAATGCCAACAAATACGAAAACCACTATATT

TGTTTATATGTTCCCTTTCTTATTATAGGTATAATTATATTAGGTACCAATTGGATTTTG

GTTGTTTTATACAATCATATCCAACACGTCATTATTTTAATGAAAGTTAACAAAAAATCA

GATACATTAAAATATTTCAATAACAAAAAAAAAAAAATCAAAAATATGTTGAACTGTTTA

GTTGTTTTCGAATATACTTCTGATAAGACACATTTTTTTTTATTTCCAAGACGAGAGACG

ATTTGGGAATAGTCGGAACAAAAATACCCTACACTGTATAGTTTAACCAATAACAGATCG

TTACCATATTTGACGTTATAAAATAAAAATATTTGTTAAATGTTAAAATAAAAAAAATAA

AAAAAAATCGTATCGAGAGGCTAAACCCCCCCCCAAAAAAAAATAACTAACTAATCTCCA

CCTATAATACTTTTACGGGGCTGTTCGCTTGTGTATATGTATTTGTGTGTGTGTGTTTGG

GTGAAAATTTGTTATTTTTGTTTAGTTATTGATGTCAGCGGCGGCGACGTCGTCGGCTAA

TTGTAACGAATCCGCGTACTCCGTACCCGCGGCGGCGTCATAGTCGCGGTCAGCCCTTTT

GCCCAGTCCGAATCCGTATTGTAGACGTGCCCGTCTGACGGCCGGGTGCCACATGGGCGC

CGAGTCGTCGCCTTCACCGTCGCCGTCTTCCCATTCCGCCCCCGCACCTCGTTTTCCCAG

CCCGAAAGCAAACCGCTGGCCGTGACCCATATCGTCGGCCGTCCTCTTCCTCTCCTCCGA

CAAGTCGTTGTACGTGTAGTCCGATTCCCGATCGTCCACGTTTAAAAACGACGGCTGCGA

CACCCGTTTTCCGATTCCAAAATTATATTGTGGCGTAGCTCTCCGCCCCAGGCCAAAACT

ATAGAAGGATGGCGAAGCCCGTTTTCCTAAGCCGAACTCGTACTGCTTGTGCGCAGCCCG

TTTGCCCAGACCGAATCCGTACTGCTTTGACGTCGACCGTTTGCCTAACCCGAACTCATA

TTGCCGGTAAAGACGTTTGCCTAGTCCAAATCCGTATTGTTTGTGAGCACGTTTGGCCAG

ATAGCCCAACGGAACCAGTGGATCGTTGAAATAACTCTCTGCCGGAGACGTCATATGCAT

TGACGGATGACCGTCCGCCATTGTTTGCATAATATCGGATTGTTGCTGTTGCTGCGCCTG

TTGACTTTTAATCCCCACTTTGTCTTCGGGTCCTGCCATTGCATCCGTCCATACCGCTGT

TGCGATCACTAAAATCCACATACAACAACTATGATGCATTTTATCCTGTGTATATTCTCA

GAGCTGGTGCGTGTAAAAACTACCGTGTTGCTGTGGTATGTGTTTAGGATAGAGTCGTCG

GTTAACAACAACTTTTCAAAGT

>AstB Unigene0029473

CGCGGCTGACCACAGTACATATTTTTATAGTATTTATAATAATATTATATCAGACCCATT

TCCGGCACATCTCGTCCATATACTGCACACGCGGTCGTCGTCGGTCCGATATCCGCGATC

TTTTTTTTCCGTCCCAAAAACACATTCCATAATATTTTCGCTCTCCGGCGCCACACGCGC

ACACGTTCGTGCCGCGACTTGCACCCGTACGCCTTTGAGTCGTCGTCGTCGTCGTCCGCA

CACACGGCGTTGTACGCAACAGGCTTTTGATCCGCCGCCGCCTTATACGCTAACGGCACG

CAAACGGCGGTACACGCGTGTACATTTAACACCGTTCGTCTGACTACCGGATAACCGCAG

AAAAGGATTAACTTAAGTGGCGGAATGCAGAATGTTTTAAGGAGGATAGCGGCCACATTG

GTTATTTTATGTCCAGTAATTGTTTTTTCAATACCGGAGTCTGCTATTCAAGCATCATCA

ATCAAATCAAGTCAATCTGAACAAGATAACTCGGACTACCCAAGATCTTTTGACGAAGAT

CAAGAGGTAAAACGGGCATGGCGAGATTTGCAGAACGCTGGTTGGGGCAAACGTGGTTGG

CAAAATTTAAAAACCACGTGGGGCAAACGGACACAGGACTGGCAGAACTTGCACTCGTCG

TGGGGCAAGAGGCAGGGCTGGCAAAAGTTACAAGGTGGATGGGGAAAACGCGGATGGAAA

GATATGCAGTCCGGGGGCTGGGGAAAGAGGTTTAAAGATCAGCCTAGCACTGATCAATTG

TCTCAGTTTGATGAGTATTTAGACAAATATGAAGACGAAAATCCTAATGAAGCAGAAAAA

CGATCATGGGACAATTTCCAAGGATCTTGGGGCAAGAGAGCAGCTGATTGGACAAGCTTT

AGAGGTTCATGGGGGAAAAGAAATCCCGTGGACTACATGAACGAGTATTCAGGATATGGA

GACAACGATAATTACAAAGCTTATATTTTTCCGCCAGGTTACAATAGTTATTTACCCAAC

TTCCAAGCGGAATACGAAAAATGAGACGTAAATTCTCAATTTTTGAAAGTACTACCACAA

AAAAATTATTAATTATTAGCCCTAAATTTACAGACTATATA

>AstC Unigene0013997

GTATAGATATATATTATAATATAATATTATATTATTATTAATTTATATATTTTTATTATT

ATTATTGTTATTTTTGCAACACGAGAATAATTTAATAAAATAGTCGAATTTTGGGGGTGT

TAAAACTATACGGATAAATAACTAGGTAAGGATAGCTATAATATATTATAATCGAAATAA

ATAGTTGAATATTAAAAAGCATTGTGTATTTTTATTATATTATATTAAGGGCTAATATCC

TGCCGTAAATGATAATTTTATGTTTTATAATATTATATGGACAATGCGCGCACATAGACT

TAACCGAAGCACGACACCGCGTTGAAAGCACATTGCTTCCAATAACTTCTCTTCTTTTGT

GCGCGATACGAATCTGTCCGGGCGCGGATTTTGTTCATCATTTGTTTGGCAAATAGATAG

TCAATCAGCGCAATCTCGAGGTTCTGATCATCGCCCTTAGTTTTTATAGTATAATCATTG

ATGGCAGGATCTTCAATTTCACGGAAATCATCTTTATCGCCCATTGACATTATTTCAGGA

GAGTTTTCTCCAAGTTCTCTTTGAAGTACTCTTTGATCTATGACGTTTCCTGTGGTTAGT

TTTGGCAATAACACAGTTAGGACGGCGAGTGTCAAAATTACTCCATAAGATATGAATCCC

ATTTGTGTAGCCATTTTAAAGTGTATCTTTTATGAATAGTCG

>AstCC Unigene0053841

GGTATTGACAGTACCTATTATAATTGATGAAAAAACAAGCGTATGAAGATGAGGGCGGTA

TAATATTCTCAACTGACATTATGATAAAAAATAATAATATGTATATATATAGGTAGGTAG

TTAACAGATTTTATTTCTTCTATTTAAAACAAGACACAGCGTTGAAATAACATCTCCAGT

ATAATCGTCCTTTTTGTTGATTTCGTCTCTGTAGTTCCATTCCTGGGCTTGGTCTAGGAG

CTCCGCTAATTGGAATTGATCTTTCCGGATAATATCCTTTCATATTTCCAGAATTATTTC

CATCTACAGCTTTTTGTAACGCCACCATTAGTCTATCCAGCAATAGTGCAGTCCTTTTTG

GCACAACTACCGGATATTCGTCGAATTCGACCGATACGTTCTTTTCGTTGTTCGGCGGCT

GATCTTCGGGCGTCCGTTTGTCGTATCTGAGTAATGTGAAGGGGTCCAACGGTTTTTGAA

ATGTCGAATCGCACAACGCCACCATACACAACGATATCAGAAACAAAACTATTTTATTCC

AAACAACCATACTGATGATTATTCTATTAAAATCCACCTTGTCGAATAATAATGATTTTA

TACAACTGCATTGGTGTGCAACTCGAAAATGTATGATCGGTTGCTGGATAAATATTCGTT

>AT Unigene0043447

GCACCCACGTGAGAAACGCGCACCCGAGGCTCAAGTTCGACCGGTGGATATTCCAAACTA

TGGCAGTTAACAACAATATAATGGTCCGATTGTTGGTTATAGAGATCACGTTTTTGATAT

TAGCTGTCGTCAACTCATACCCGGCGTTTGAAGATAACGAATTTAAACATAAAAACAGAG

ACAAAGGGAGATCTATAAGAGGTTTCAAAAATATGGACTTATCTACAGCTAGGGGGTTCG

GCAAACGAACCGACCATTACGTGAATTTAATGCCATCGGATTTATTTGTGGACAACAAAG

AAGATAGCTTCAATCAAAATATACCGTTGGATGTTTCTTTGGAAAAAATATTGAAGAACA

AGTACAACAATTTTATCGAAATGCTAATCGATGTCAACCACGACGGTTATATTTCCAGAG

AAGAATTGCTGCAGTCAATTGATGGTGAATCATAATAAAAAGTTATAAATAAATATAAAT

TATACATATTATTAAAGTCAGTTTCACTTACAAATAATTTGATTGTAAATGTCTTCAAAA

TTAAAAAAAAATATATATAAGTATTAATGTAATTGTCATTGTAGCGAATTTATTTCATTT

AAGTGGAAATTTACGTAGGTACTTTAGTTTTATGATTGTAATAATAACCAACCACCATTT

TATGTAGTAGTACCTATAAAACATTGACTTTTTCGAAAACTAAAATTGATACTCAAGATG

AAAAACGGGATCTATATATACATGAAATATGTGATGGCTGTTAAAATATTGTTTATGCTT

AAAAAATGCTAAAATATAAATATATATGTATTCTGGAACTA

>Burα Unigene0009955

GTATGATCTTTGAGTCGTAATGTCTACCATCAACCAGGATTTTTTCAAATATCTGATAGT

ATTGGCTATGTGCAACATGGCCTTTGCTAATAATAACGGTAACGGGGTGGTCGTAACCGC

TAGGTCTAGCGACGACTGCCAAGTAACACCTGTCATCCATGTCTTACAGTACCCAGGATG

TGTACCTAAGCCAATACCATCGTTTGCATGTACAGGACGCTGCAGCAGCTATTTACAAGT

TTCTGGGTCTAAAATATGGCAAATGGAAAGGTCTTGCATGTGCTGTCAAGAGAGTGGTGA

ACGAGAAGCTTCGGTATCACTCTTTTGTCCAAAAGCCAAACAAGGCGAGAAAAAATTTAG

GAAGGTAACTACAAAAGCTCCATTGGAATGCATGTGTCGCCCTTGCACGGGTATTGAAGA

AAGTGCGGTAATTCCTCAAGAAATGTCCAACTATGCCGCTGATGAGCCAGCAATCAATGG

TCATTTTTCTAAATCTATTTAAATTATTAATCAATAAATAAAGAAAGTGTTTATATTACT

TTCATTTTTATACAGTTTCCAAATTCTT

>Burβ Unigene0017703

CTACCTATATATTTAATAATTAATAAATAATAATTATAACAAAAGTAAAATAATACGGTA

AAATAGTTGCTACATATCATATGGATTTTAAAACATCAATTTATCCCGAAAAATCTCCGC

ATTTAAAACATTTGCAGTCTGCTGGTTCTTTTAGTTTAACTTCCAACGTGGCCAATTTAT

CACTCGTCAATCTCACGCCATCAGGATCGTAGCAATGTGTAAGAGCAATAATTCTCTCTC

TCAAAAATGTTTCACGGCAACAATAACATTCTTTTAGGAATCCACTAGGAGTAATGACAC

TAGGTTGTACTTGTGAATTACACGCTCCTTCACATTTGTTCACTGCTATGTCACCATTAC

ATGTCCTTTGAAGCCGCCCAAGCTCATCAAATTCCTCTTTAATGATATGCAATTCAGAAG

GCAAAGTTTCACATTCTTCTGGGTTATCTTCTGCAAGTACGTTATGTACGAAAAAAATCG

CGATAGAGATGATAAACAATTGAGTCGTGTACATAATTGACAATATAACTGCCAATACAG

ATTATTGAAGTAATCCAGATCAGTGACTGATCAGTTAACAGTG

>CAPA Unigene0012156

AAGACTAACAATTTGATTTCAAAATAAAATATCGTAGGTGGGTATCAAATATTCTATTTG

CAGCTGTATACTATTACACTACTAGCTAAATCACGAAATTATTTTAAGAAAAGTAAAATA

AATTTAAAGTACACAACACAATGTTACATTAGATTGTCATCTTCATCACCATAGTTTTTG

CTTTTCATTTCGATCAAAGTCTTTATGATCGTTGGAGTATCCGAATCTAAATACGACGTA

TCATAATTTTCTGGTACCACGACTGATCGTCCTAACCTAGGACCAAACCAGAGAGCTGTA

TTCTTTGAGTCACTCCTTCTGCCAATCCTGGGAAAAGCAATGAGTCCTTCTCTTTTGTGA

CTTCTCAGTTCTCGTTGGGTATTGTAAAAATTTTCCATTTGTAAAGCAGAGTCGATACTG

GACCTCCCGACTCTGGGAAACGGTATCAATCCTGCAACTGATTCCCTTCTATCTCTGTTA

CTGTCTCGTTTATACTCGTCACTGTATTCTGAACCATGGCGCAGAGCATGAGTGAAGAAA

AATGTCAGTGTTAACAGCAACGCTGCAGCTATTTGCGTTTGTAAATTTTTCATCGCCCGG

TAAATGTTATTTGAACGGAAAATAGAACGGACGAATACGCAATGTGATTTTTGGTTTTAT

GTCTTTCGTGTCTGGGTGATTTTCAGACAATTTCGTACGAACACCTCACGCAAGCTCCTC

TACCGTAGCGCTTTCGTCTGTCGTCAAAGA

>CCAP Unigene0044769

GATCAATGTGCATCTGACTTGCCACAGGCCATTCATCGACAGCCATGAATCCATCAATAT

TGACCCTCGTCTGGATGAGTATACTAGTATCTCTAGTCCAAACTGTGTTTGCCGATGACG

TCATTATGCAAAAAAGGTATTTCGACAATGATAATCCGGTAGCTGAACCAATCAGAAGAA

AAAAACCTTTTTGCAACGCATTTACAGGTTGTGGTCGTAAGAGATCGGATGAATCAATGG

CCACCTTGGTCGAACTTCGTTCAGAACCGGCTGTCGAAGAGATTAGCAGGCAGATAATGT

CTGAGGCTAAGCTCTGGGAGGCGATTCAAGAAGCTCGACTTGAATTATTACGCCAACAAA

GACAAAATAAAGCAGAGAGAATGGAAATGAAACCTTTTCCCGTGGGTTTAAGAAGAAAAC

GTCGATCTTTTGCCGCCGGTGACAAATGCTAAGATATTCATCAGCTCTGTGAAATTTCTT

TTATTATAATTATAATAATTATTATAATTTCCTTATAGTAAATTATTTAAGTAAATAAGT

CAGATAATACAAAACATATACCTACATTATTTTGTGAAAAATATTAAACCAAATATAAAA

GTTTAAAGGGAATATAATTCTCATTGCAATTATAAGCTCTCCCTAGATTTACAATCAAAA

TATAGATAAATTATTATTAATCAAATAAATAATCTTTGTTAAAATTATCAATAAAC

>CCHa1 Unigene0034430

CCGTAGGTAAACTTACCTCTCAGAGTGTGTATGAGCAACAGGTGAGGAAAATACATCAAG

ACCACAGAATACAAAATGCACAAGTCTTTCGTTAAAATATATGTTTTCGTGTTGATTATT

TGGGCTGTGGAAAAATCTGATTGTAAACAAGGAGCAGCATGTTTAAATTACGGTCATTCG

TGCTGGGGAGCTCACGGAAAACGTAACGTGGATGGGACAAACGATCTAGACACTTTGTTA

AGATATCGAATGGCTTTGTTTAAGAAATCTGTACACAAAGACTCATCTATGGATCCAAAT

CCAGAACAATCACAAGAAGATATACCAAACTATTATAATATTTTCAAACATTATTCGAAA

ATAAATTCAATGAAAACAAATAACGATGACACCATTGATACCTGGAGCGTCGAACCGAGC

AATCACTTACCAAACGGTAGGTCATACTACGAAGATCAAGTCTTAGATCCCAGGATTGAA

TATAAGATAATGAAAATATAGGCGAAGAACTATCACAATTTATTCTATAACATTAATAGC

TAATACTTGGAGAAACCGGAAAAAATTAATATTTCAAATAATATGTAACAACAATACCTT

TTAAATGTCAAAATAATGAGGTTATTGAAATTTTGGTCACATATTTTGATTTAAATACAC

TAA

>CCHa2 Unigene0056057

TTTTTATTTTTCTGTGGAGGGATTTTTAAGTCTAATCGATATGTCTGACGACAATAATGA

GAATATTATATGTGTCCGAATCAAGAAAACAGAATTGTTTCAATCTCTCGATTGGTTCCG

TCTTCACATGTTGCTGTCGTTGCTAGGAAGATTATTTTTGCGCTCATTGTACATCTGCAC

CATTTTTTGCCAAAATGGTGTAAAGTGCGGTGACGACGGCTTTATTTTAAAATACTTATT

GATCGCGTCGTCGATCTGATTCTCTTCTTCATTCGTTGGTAGCCAATCTCTAGCCGGTTG

TTGCATGGGCATTTGAAAAGATCGTTTACCGTGAGCGCCGTAACAGGAATGGCCAAACAT

GGCACACCCACGTTTAGCTGCTCCATTATCCACTGTAAATATGACTACAATCGCTAGTAA

AACCATACAAAAATAGCACGTTGTTGCGATTTGTGGTCTCAAAGGCATGGTTATACTATA

CATAAGCACAAACACTGATAGTCAATCGTCAAAATAAGCTTTTGCCCCGAAAGTGACCCG

>DH31 Unigene0016990

ACAAGTATCACGGTCGTTGGAGCAACAGCCACTGTACCACGTATACCTCCTACTGCAGCG

GACTTCGCTTTCAGTCGGGATATCATCGTATGTTCACAGGAAACATGATGGTGGGAGCTT

CGGTGGCGTGTGGCTTGTTGATCGTGATACTTATGTGCACTATCCCAGCATCATTATCTG

CACCTTATCCATTGCTACAAGGGCAAAATAACGCGTATTTGTCATCAGAAAATGACGGAG

ACCCTGAAGTCATGTTGGAATTATTGGCCCGAATAGGCCAAAATATCATGAGAGCAAATG

AATTGGAAAATTCTAAGCGTGGGCTTGATTTGGGTTTAAGCCGAGGCTATTCAGGCACAC

AAGCAGCAAAACATTTAATGGGCATGGCAGCAGCTAATTTTGCTGGTGGTCCAGGACGCC

GACGTCGCAGTGATATGCTTCCTAAGCTTCTTACACCTTAAACTTTTAGTTTGTTATAAA

TTATATTTACAAACTATTAAATTCAAATATATACTAAATATTATATAAATATATGAATTA

TATATATATAATAATATAAATAAATATATATATATATATATATATAGACATATTACAAGT

CAATGATTATTTTAAGCATAATCCATCCATTGTTTTGT

>DH44 Unigene0030947

AAGTTATTATTCTCGTTTATCTGCGCCGCAACAAGTTCGTGTCTTCGCCGTCTTTGGGGC

GCAGCAACAAGTCTTCAGATTGCACAAGTCATCGTCACCGACATCACCTCTGTTGACGCC

GAGACACGCTGCAGTCATCTTCCTGTACCGTATTTGTAATTTATCATAGAATTACAATTT

AACCTGGTGGTTTATAAGAACTTTGACATTTTAAGCCATCGTCAGTGCTAAGCCAAAAAT

CATAAATACACAATGAGGATTTTGGCGATCGCTTGGATTTTGGTGGTGGTCACGTGGTGC

TGCGACAGCGCCGTTATCTACGAACCAGTGGTTCAAAATACCCGTTACTACGAGCCTAGA

ATTCAGGACTTGGAGCTTCTCGATAAAAACTTTTTTGATATGGCTTCGATCGAAAAGAGG

AACGGTGCAATGCAAGGCGAGTCACCGAGAAGTCGTCCTAGTTTGTCCATCGTCAACTCT

CTAGATGTGCTCCGACAAAAATTAATGTATGAGGTGGCACGGCGACACGTAGATGAGAAC

CAAAAAGTGCTGTCACAAAATCACCAGATTTTGAAGAACCTTGGAAAGCGGTCATTGTTT

CCTTTTCTCGAAGTACCACGGAGGTTTTAAAGGAAACTATTTGGAATTCGATGGTTTAAC

AGCGTTATTATTTATGAATATTTTATTTAAAATGCAATATAATTATTTTATTTGTTGACT

TTACCTAACATCGTAGACAGCCAAATCATTGTAATTTTATTTTCAATTTATATATTATAA

CATGTATTTCCAGTATATAAAAAATATATCTAGTATAAATATATTATTTCGTATATATTA

TACTTTAAAAAAACTCAATCCACGGGCATTATTCACCATAATATTATTATACATACTTG

>EH 1 c17200_g1

GTTCAGACTTACGAGGGTCTACGACTAGCACACAAGCTTTTCCGATCTCAAAAGAAACAA

ACTTAAGTTCTATAAGACTTAAAAGATGACCACTTCGTCCAAGAAAATCGCCTTCCTGGC

CGCCGCACTGTTATTAACCGCGATCGTCGGTTACACGGCTGCGGACATGGCGAACGTGGC

GATATGCATCAGAAACTGTGCGCAATGCAAGAAGATGTTGGGCGACTATTTCGAGGGGTC

GCTGTGTGCGGACACCTGCGTCAAATTCAAAGGCAAGATGATACCGGATTGTGAAAATAT

CGAATCCATTTCACCGTTCCTCAACAAGCTCGAGTGATCTGCGCCGCGATCCCGGAATTC

GAGTAAAGTAGTTAAACAATGAACATTCATGTGTATTGTATAGTCGATAGTATGATATTA

TTCATTTATAACGTGTACGTGGTTTTTGTTTAATTCGAGTAATAAATTACATTCGTTACG

AGTAAAAAAAAA

>EH2 c17200_g2

GTATTGTCTAAAGGGTTTACGACACTGCAGGAAGAAGTTTCAGCGCTGCAGTAAAATATT

ACAGGAGAACTTCAACGATGAACACTACACTCAAGAAAATCGTCTTTTTGGCCACCGCGT

TGGCGCTGATCGCCGTCATCGGTTACGCGGCGGCGGACATGGAAGACGTGGGCATGTGCA

TCAGGAACTGCGCGCAGTGCAAGAAAATGTTGGGTGCCTACTTCGAGGGGCCGCTGTGTG

CAGATGCCTGCGTCAAGTTCAAGGGTAAGATGATACCGGATTGCGAAAACATCGATTCCG

TGGCACCGTTCCTCAACAAGCTCGAGTGATCTACATCGCCAGCACACTATAAGACCGATT

TTATTATTTACTCACGTTGAATTATATTATACTCAACCTATATATGCGATTGCGTGCAGC

CGCATAATATATACATATGTAGATACTAGTTACCGACTTTGTGAACAGAATGTGTAATTA

TAAATTACATGGTTTAATGACCTTACATGATCATTTAAAATGAAATTATAATCCACCAAG

CGACTGTACGATATTGCGTGTAATCAAACGACAATTAATAAAACAGACCGAAT

>EH3 Unigene0032823

ACGTATAAGTCGATTACAACATCGTACGATTTGTTAGTGTGGATTATGTTTTTATTTCGA

ACAAATACATAAAATAATTTCACAATAATTCATTAGCACTATCGACTAAGAACGAAGTAT

GGCTGCAGCCTGTATGTGTAGCGTATATGGTGTATGTTATTATTAAGATGAACTGTTTTT

GAAAATACATTTTATCATATAAATATAATCGGTCCAAGGGTCACTGGTGTGAAATCACTC

AAGCTTGTTGAGGAACGGCGCCACTGATTCGATGTTCTCACAGTCCGGTATCATCTTGCC

TTTGAACTTGACACATGCGTCCGCGCACAGCGGTCCTTCGAAGTAAGCGCCCAACATCTT

CTTGCACTGCGCGCAGTTCCTGATGCACATACCAACGTCCTCCATGTCCGCCGCCGTATA

ACCGACGATCGCGATCAATACAAGCGCGGCGACCAGGAAGACGGTTTTCTTGGCTGAAGT

GTTCATCGTCTAAGTTTTCGAGATGAAGTCGTTGCTTTGCTTCTTTCTGTGCTGCTGTGC

AGTAGACCCTCTGCAGGAAGATCGGACGATACACTGACGAAAACGGCAACGGTCCCGTTG

GTTTTATACCAATGAAAATGCTTAATCGAGTTTTTTTTTTGTACAGCCTCACGATTGTAA

TACGCGTTATTTTGGCGCCG

>ETH Unigene0053622

GACATCAACAGTCGTGAAGTCTACACATCATCACTGCAAGTCGACATTTTCGAGTATTTC

TTCGTTGAACCGGAAGTCGATATACAGAATAAAAATGAGTGGAAACTTGACAATTATTGG

TTTGGTGTGTTTGCAAATACTGAGAGTAATGTCGATCAATGAATACCCCGATAAGAAAGT

ACAAAATATTTGGTTGACGGACATGGACGATAGACAGGCCGCGTCCAGAATCGAACGCAG

TGATCAGTTCGAAACGGCCAGTGACGTCATAATGAAGGACGCGAGTGTATACCCAAAGAT

TGTCCGTCGTGGATTCGCGGGAGAGGAATTTTTTTTGAAGGCGTCAAAGTCGGTGCCCAG

GATCGGTCGTAGGAATAACGATATCCAGGAATCGCCCAAAAGATCGTTGAGCAAAGATCA

AGTGAACATGGTGGAATACTGGCCGTACTTACAGCCGAACGACATCAACGACTTGACCAG

GAAACACGACTTCGATTTGCCGTACAACTGCCAACAACTGGACGCCAAAACCATTTTTTT

GGTGGACATGTATAACTTCGTGTGCAACGATCAGTTCTATTGTTGTGCACCAGCTAAGCG

CACCATAGCCAACTCCCCAAACGCGAATTCCTTGTGAAAACGCGGAGCACATATTACAAC

AATATAATAATGCTAATAATAAAATGTAGTCAGCCTTAGATTTATGGGGAATATGTTTTT

TTTTCATAATTATAATTATTATTCGTCACTTATTTTGAGTGATTTTATTTACAGCTGATT

GAAAGATAATCAACTACCGGTACATTATAATTAAACGCGTGATCATTGTTTTGGCACGCT

TTAATTAATGTATTAAATT

>FMRFa Unigene0034006

GCACGCGTCGGGACAACCGAGTCGTACTCCTCGTCGTCGTCGTCGTAGTCATCATCGTCG

TCGTCGAACATCGTTGCATCTAACATAAAACGCAGTCGTTTCGGCTTTTACGCGCCCGAC

CCGCAACCGGAACGGAGATTTGAGTTTTCCCGGCGGCCCTCGCCCCGCAGTCGATCGGCC

ATCACACGAGTGAATTTTTTACCGAGACCGTCATCGTGTTATTTTCGACCGATCCACGTC

GCACTAGCCTGTTGCTGCCCCGCGGACCGTCGACCATAACAATACCATCAAACATTATAT

TTTTATACACAGTACAGATTTATACAGTAATTCGTCGTCATCGCCGTTTCTCGATCGCTT

TATTTAATCAGAATATTCTATATCCAGCGCGTTCTATAGATATCGTGTTATATATTATTT

AATCGTATAACGTATACGTATATACGTGAAGATCATTCGTGGTGCAATAATGCTTTTGTG

TCTGTTGCCGGTGACGTTGACGTTGGCCGCGTTGGTCACCGACGGAGTGGCCGACGCGGC

CGCCGCTGACAAGCGGTTCGCCCTGCGGCCCGTGGACCCGTTGACCAGGCGCAGCGCCAT

GGAAAAGAACTTTATGCGGTTCGGGCGGGCTTTCGACTGCAGTTGGACGGCACCGTCGGC

ATCGGCAGTCAAGCGCAGGGACCCGTCGTCGGCAGTCGGACGCCGCGTCGACTCCAACTT

CATCAGGTTCGGCCGCCGCGACTCCAACTTCATCCGGTTCGGCCGGGGCGAGGTGTACAC

GCCCGGCGACAACAAAATACCCAGACGCCACTACGACGTGGACGTGGACGGGTTGGAAGT

CCGGTTCGGCCGGTCCGGCGGGAACATCGACCGTAGTCCGTTCGGCGCTGCGCTACCGCC

GCCACCTTACGACGACCGCCGCTGAACGTTACGCGATATTATAATATTATTATATAATAC

ACAATACATAATATATACATACCTATACTATATATACTTACTATATATTTAAAGTATTGC

TTAAAAGAATAA

>GPA2 Unigene0030917

TAATATCATGAATCGTAATAATAAGTAGTAAAACTAATAATCCAGTTCTATAATGTGCAT

GTAACTAAAATACTATTATATAATATTCAGACTAGACGTTGAGTGCCGATTTGTACAACC

GAAATATAATTATTATTATATTATTAAGTACAGTATTAATGGTATTAATCATTTATGAGT

TATAAATTACGATTGATGTAACTATAGAGTAATATAGTATATACTATAGTTTACTCGCAT

GCAATAGTAATAATAGCGTTTGCTGATGTTTAAAAATTCTAGTCAAAAATATTTTGTCTC

CATGATGTAGTGAAATGTCCAGTAAGAAAATAAAAATCACTCCTTCTTGCAGTGATAACA

CGAACAGCTCAACGCTGATTTGAAGACCAATTTTCTCACGCCCTCTATACAACGGACGTT

GACTTCGACGTTCTCAGTATCCATGATGTTGCAGCATTGGCCTACGGACGTGATACGTTG

GTGCGGGTTGATCATCACAGTGTCAGCTGGAGACGGCACCGCCCACGATTCGCAATATCC

TCTGCACGCGTTGGTCGTGATAGGAAATTCCACACAGTTGGGTATGCTTATCTTCCTAGT

GTGACCAACTTTGTGGCAACCTGGTTTCTGCCAAGTGTTTGAACCAGTCGCCAACGGAGA

TGGCAGGACAGCCAAGAAGAACATCAAAATCATGACAGCGTTTTTCCACCACCGAAACTT

GTCCATATCACTAAATTTAGTCTTCAGACTCGGTCTTTCGCGTGTCCGGCGACCACTTGC

CGACCGACGGTTTCGATATTAATGAACGAGTGGCACGTGCTTGCAGACGACGACGACGAC

GACTGTACCAGCGACACACTGACTGGCGTTCGTTCCCCCCG

>GPB5 Unigene0022160

GCCGATCATCTCTCGCGTGGTCGTCGTCGTCGTCAACAGTGTTTACGACCGCCGTCGCGA

ATTCCTTCTTCGCAGTCGTCGCCGCCGCCGTCAAATGGCAATTTGATTTTTCGCCCAAAA

ACATATTATTATATATTGTCGGCGTTTTACAATACCTAATAATAATTATTTATTATTAAA

TTAAATTAAATTACCGTTAATAAAATATAATATATATATATATATATAGTCGCACTACAC

GCAGTGTATCAATGGTTTGCGTAACGGTGTTGTTTGCCGTATCATCGATGATCGTATGGT

CTTCGGTGGCGGGTTACAATATGTTAGACTGTAATCGACAACTGTCCACTTTTAGAGTGT

CCAATACCGACGTGAACGGAAGGACGTGTTCCGATGAGATCAACGTGATGTCATGCTGGG

GAAGATGTGACTCAAATGAGGTATCAGACTGGCGGTTCCCATATAAACGTTCGCACCATC

CGGTGTGCATACACGCCGGTCAGGTGCTCACCGAGTTCGTGTTGAAGGACTGCGACGAAG

GCGTTCTGCCGGGTACGGAACTATACGTTTTCCCGCAGGCCACGACATGCAAGTGTCACA

CGTGCAAGTCTTCAGAAGCGTCTTGTGAGGGGTACCGGTACAGAGATTTCCAGTTCATTT

CCACCGACGAAGAAGTTTGAGGAAATGTTTATGACTTGAAAAAATAATGTGACGGAATTA

CAATGACAGCAAACCGAATACGACATCATATAATATATATGTATATTATTATTATTATTA

TTATTATTATTATGCGCTTCCGTTTTTCTTTTTCCCATAATATCCTTCATTTCTATTATC

TGCTCGTCTATTATAATGTCGTCATTATCG

>ILP1 c15478_g1

CATCCTTCTGCAGACCATATCCATACTAATTTAAATATTCATTCAAGAATACAATGAAGA

TCAGTGTAAATTTGAGTATACTCCTCTTAACATTAATCATCAAAATTGTAATAGCCAATG

CTAATCTACAACTACCCCCACAACAATATTGTGGTTCTAGTTTGGCAAATATAATGCAAA

TTGTATGTAAAAACAAATATAATGGACCATCACATGGAAAAAAACGAAATGAGATTGATT

CAGATTTATTGGATTACAAAGACTTAGAAGACTATAATGCAGTTGACTACCCATACCAAT

CAAGACAAGAAGCAATGTTATTTATGCCCACACGTATAGTACGCTCTTCTAAAAGAACTA

TCATCGATGAATGCTGCCGCAGACCCTGTTTAATATCTGAACTAAAAGGTTATTGTGCTA

ATCAAGATTAATAGATTTAAGTGTTTTTAGATGTTAAATAAAGTTTAAATATTATTATAT

TAAAAAAAAAA

>ILP4 c25074_g2

CCGAAATATTAAAGACTTTAAAGTCATTAAATCAAAATTTTAATCTCACATTTATGCTAT

ATTTGAAAAAGTCTAAATTAATGCATTTAAATTGATCTTTCCGAATATGTCAATTAAATG

GTTGCTTTTCATTTATCATAGTGTGTGTAGAAGTGTAGTGCCATAGAGTTGGAGAGATCC

TGAATTGTGCTGTAAATTTTAACCTTATTGGATCTTCCCTTAGAACCCCTACCTACGTAA

TATTAAAACAGAGAAAATATATAAGACCAACAGAGGTTGATAATCGCCAGTTCACTAGAT

ACCTTTCAGCGAACCATATCTACACTTTTTAAATTTTCATTCAAGCGAGAATACAATGAA

GATAAGTCTGTATTTGAGTGTACTCCTTTTGGCATTAGTCATCAAAATCGTAACAGCTAA

TGTTAGACTGCAGCGTTCACCTCAACAATATTGTGGCTCTAGATTGGCAGACATCATGAG

AGTTCTGTGTAAAAACAAGTACAATGGACCAAATGGACAAAAAAGAAACCCAATTGAATC

AGATGTATGGGATTACAAAGACGTAGAAGACTACAATGCAATTGACTATCCATACCAGCC

AAAGAAAGAAGCAATGCCATACATGCCCTCACGTTTCCAACGCACTTTTAAAAGAAGTAT

CATCGACGAATGTTGTCACAGACCCTGCTATTTATCTGAACTAAAAACTTATTGTGCAAA

TTAATAATAATAATAATAATAATATGGTACCTGCAGTATAATAATAATATAATACTTACT

GCAGTTTGTAGTAGCCCACACGCGCGCGCGCGCGTCGCAAACTCCTCGAACACTATTAAT

AAATAATAATAACGATAATAATATATATTATACAATAATGAAATCTATCGTTTCTCGCCT

TTTTTTCCCTCTCGTCTGTTATTATTATTATTATTTATTTAT

>ILP5 c20960_g2

AAGCCGACAACGCCGGTCATGTGCAGAGCAGTCAGTCAGTTAGTTAATGGCTCAAGACCG

GAGTACATCTACTCAGACCGACCGCGTTGTCCGATACCTCGGCAATTCTAGATCTCGGCT

AGAATAATTACCCACGAATTACAGATTTCTATTATCTAGTTCCTGCCTGTGTGGTGGGAT

CAGGAATTCATAACACGTATATAAAAGGCGCTCACTCGCAATAATTATCGTCAGTCGTCA

CTGTTTCATTTCGGTAATACCGATCGTTGTTGAGTGTGATTAGCGATGAACTCAGCTGTG

GTATTGGTGATAGTCATGTTGCTGGCCAGCCACGTTAGCAGCTCACCGATCATAAATTTT

TCGTGGGACACGCCGAGGCATTTTTGTGGATCACAACTCGCCAACGTATTGGCGTTGATC

TGCAGCAATGGATACAACTTTCATCCGGCGAGTGATGATGTTACCGTCCCAAGCCGCCGT

CGAAAAAGAGTAATAGTCGAAGAATGCTGCGAGAACACTTGCACGCCAAAACACCTAAAA

TCCTACTGCTGGGAAAATCGAAGGCGGTGATCGTCCAGGACCCGGTACCAGGACGAGTGA

AAACTGTAACACCATATTTTGACAAAATGCCATCGGTCATCGTTACTAAAACCACGCAAC

ACTGAAACTCGGGAATAACACCGCGCCTGCAGCTCGTCTTTCATTTTGACTTACATCGTC

AAGCACATTGAAATCTCTAACACTGTTTGGTTCTACATTATACGTACACAACATGTCGTA

TATAACGTTTCATGTACAGTTTTATTTTTCTTTGTAACAATGCGCCGCGCAAAACCGAAG

AATTAAAAACAAAATATTGACTAAAGTCCTGCTGTGTTAATTTTAATTTAATTTTTATAA

GTATAATAAGTCGTTAGTTCCTGTGTTAAGTTTGCACGATTTTAGTTCACGATATACGAT

TGTCGGTGAATATAATATAAATAACGATTTTTAAAAAATTATTATAAAAACAAGTCTGTG

ATTTTACGGCGATTTATATATTTATGCTCC

>ILP7 c10146_g1

AGCTGGTGTATCCCAATCAATAATACATCGCCGAAAAATGGGAATACCACTTATAACAGA

GGAGTGCTGTTATAATCCATGTACCAGGCGAACATTAAAAGAATATTGTGCACCAAGTCA

ACAATAGAATAAAAATGAGTCAAATACTCTTTAAAGTTCAAAATTATTCATTAATTTAAC

AACAATAGTTTTGTACACAAATAATACATCAAATATCAAAAAATGTTTTAAAATAATTAC

ATATTTAAATTTTGTAAAATAAATTATCTG

>ILP8 c15326_g1

ATCAGGAATACAATTTGCTGTTGGCTCACCCATGTCTAACAGGTATATGAATGAACCTAA

CCAATTTTGTGGCCAAGCATTAGCCGATGAACTAGCCATATTATGCAAGGGTAGATATAA

TGAACCCAAAGGTAGAGCAAATCAAAAAGGCAAAAGAGGTATTGTCAACGAATGCTGTTT

CCAACCGTGTACAAGAAATTACATGAAGACAAATTATTGTCCACCTGAAACCGAAGTACC

GAGAATGAATTTCCCGATGTTGGGCCCACAGAAGAAAGTGGATTATCTGGGATACTATTT

TCAACTAATTAGGCATGTATTAGGACTAAACATGGAGTAACATGAGGAATGAATACATCA

ACAAATAATTAATTTATCTAACTTGTATGTATTAACATTATAAATTAACCGTATTAAAAT

>ILP10 c15424_g1

CAGCGTTAGTAGTACTACTCATACTCTTTAGTCTCTCCTAACGAATATACCAAGCCATAA

AATCAGTAATATTGTTCACGGAGGATTCTCTGCACAAATAAAAATGAAGCTATACATTAT

TATAACTACTCTTTTATCCATTGAATCAGGAATACAATTTGCTGTTGGAACATCCCTTTA

TGACCAATATATGAATGGGCTTCATACATTTTGTGGCCAAACATTAACTAACGAACTATC

CTTATTATGCAAGGGTAAATACAATAATCCCCAAGGTACTACAAATCAAAGAGAAAAAAG

ATCTGTTGCCGATGAATGCTGTTCTCGACCCTGTTCAAGAAACTACATGAAGATAAATTT

TTGTCAAGAACCCGAAGAAATGAAAACGGATTCGGTGTTGTTGGCTCCACTGGAGGATGT

GGATTACATAGGATACTATTTTGAACTTCTTGGTCGTATATTAGCACCTAACTACCTAAG

AATCAGG

>ITPL Unigene0031227

GAGTTGTCATCTTATATTTCATTTTTAAATTAAATAAATACAAAAAAAAAAAAAAAATAC

AGTTTTTCTTTTTATATAAAATAAAAAATATTACACATTTCTTATTAAAAGTTATTAAAA

TTTGTTTTCTTTTATATATAATAATATAATTTATAATACAACTCTTAGAAATTAAAAACA

AATAAGTTTTATAAACAAAAATTAAAAACAAAATCTTATAATCTATTTAATAAATTAGAA

AAATTCATCTAGGGAAAGTAAACATATTTCTTCGATATTATTTTGTATGTAATGCTGTTG

CTTTCTCTGAATAGACTAGAATAGGTTTTTTTGACGGCCAATGTCTGGTTTTTAAATAAA

TAACCAAAAATAAATAAAATATATGTGGGGAGTACATACACCATATTTTATTATATATTT

TATTTCTTCGATATTATTTTGTATGTAATGCTGTTGCTTTCTCTGAATAGACTAGAATAG

GTTTTTTGACGGCCAATGTCTGGTTTTTAAATAAATAACCAAAAATAAATAAAATATATG

TGGGGAGTACATACACCATATTTTATTATATATTTTATTTCTTCCCCAAGAACTCGACCA

TCTGGTTGTATTGAGTCTCCTCCGTTAACATTTGAAGAGACTCCAAACAAGCCTTAAAGT

ATACCGTGTTGAAGCACGTAGATCTAAGCCCCAACCCCGACAGGATCAGCCCCGTGCAAC

TGTTTAATCCACTTCTGTATCTTTTCCACTTCATCCTCAAGCAGGAGCACGTCCAGACAT

CCTTTGAAAAAATCTGTCGTGAAGCAATTTTTCCTGCACAGTGAGTGGAGTTGTGGTTCC

CGGAACAGGTTGTAACAGTCCTCGCACACCCTGTCCAGTCGGGCGAATATCGTCTTGTCG

TACACGCCTTTGCACTGGATGTCGAAGAACGACCGTTTGCTGAGCGGGTGATCGATGCCC

GACAACGAGGGCGACGACGAGCCGGCCACGCCGTTGCTCGACGTCATCCGGTGGTGGCCG

CCTGAGTGGTGGTGTAGGTGGTGAGCGGCCGGCACGGCGACCACGGACACAGCCATTATC

GCCATCAGCGTGGCGACCAGTACCAGTGCGGGTTCGTAGCCCATCACACTGTATTTCGAC

ATTGAGTTACGAGAAACAACGGCCGTGGCTGAGGCGGCTGAGCGGGCGATGCTGCCCGTT

TGCGGTGACGTTGCGGCGGCTGTCTGACGGTGGCCCAGAGGACCTCCGGCTTTCGGCTGC

GGCGTCGGCGGTTAACCGAGCGGGACGACGGGCGAAACGTGTCCGGTCGCGGTGTGAAGT

TTATACAGAACCGTCCGAGGGTGCAACGGTAGCGCACTGGGCCCGGTTCAGGTTTTCGAG

TGGTTGAGTACGAATATGTATTCGACGAAACTGTCTCCCGACGGTTTGTACGCCGGTATC

ATTTTAGTGGCGCGGAGACCATATTTCCTCTCGCACCGTGCAAAACGAACTTGGTACCTC

CGGATCAGGCGGGCGGGCCTCGGAGGAGACACACAAAAAAAGAAAACGCACACACTTATG

GAGCGGCGACTCGGGGATAGAGGGTCGAAGAAAGTCTCAAAAAAAAAAAAAAAAAAAACG

GACGCGGTTCGAAAAAAAATTCCGAAAACGCGAGTGTTTGAAAAAAAAATTTATTAAAAC

GACACTCGTCGAGCGGACGACTTTTTAAAATAGTGCGTGTGTTTAATATTACAAAATCAC

AACAATCGTATACGTATCGAGCAATATATGTGTGTGTGTGTGTATTTGTGTGTGTTTAAT

TGACGGCGACTCGTTTTAAACTAAGAACGAGACTGACGAGCGTTGCACGTTTATGCGCCA

AACTGCGATCCACTACTCCATCGCGGCGAGTCGCCGCCGGACACGGTCCCCTCTCTCTGA

GCGCCGG

>LK Unigene0012286

TAACTGTAGCCGTAGGGATTCCAACGCAATAATACAGCATGGTAAAAATTGGTTTACCCT

GGTTGCTGCTGGTTCTAATGAAACTCACCAACCGAGAAATCAAGTCAGATGAGATTTTAT

CCTTACAAGAAGTGTTTGATATATGCCAAGTAGAACCTAGCACCGAAATATGCGATCTAC

TGGAAAAATCCTCGAGTCTAGTTGATCTAAACAAATTGGATTTACCAGAAAAGCGCCGAC

AGAAGACAGTATTCTCCAGTTGGGGCGGAAAAAGACAAAGCACTTATCCATATGGGGGAA

AACGTCCAGCGTTTTCCAGTTGGGGAGGAAAACGAGCTTCGGATAAACACGGTCGACCTA

AACAAACATTTTCAAGTTGGGGAGGAAAAAGAAGTGAATTAGATGGTTATGATAATGGAG

AAATACTTGAACCCCAAATGGATAAAAGGGATTTTAATGGGATTAAACAAGATAAAAATA

ATTATAGAAATAAAATGACCAAAGGAATTCATGCATTGTTTACTATTTTCTCCGACTGGT

CAAGGGATCCAGAAGAAAAGAAAGGCATTCGATACGCTGGTATTAAAAGTATGAGGAGAA

GTTCCGATTTTTTTCCATGGGGTGGAAAACGATCTACCGGTGATAAATAAATATGTTTTA

CTTTATAGTTGATGATAGGTGCATTCAATTTTGTCAAATTTTACTATAAAAACAAAAATA

ATTTAATCCTTATCTTACTTAAGTGACGATAAGCTTACTTAGGTGGTATTAAATTGGTAG

AAACGTATATTATGTATTATCAAAATAAACATATTT

>MS Unigene0026279

GACAATCTTCAAGTGTAAGGATTAAACCTGTCCGACATCGGCCTCGACCTCAAAAGACAA

AAATTACTGCCGATTGGCCGGCCGGAAATATTTGGGGTATGCGCGTCCCAATAAAATAAG

CGAACAAGCGCTCAACAAGTGCCGCGGAGTGTGCGTGAGTAGGTGTTAGTTCCGGAGCTA

TAAGAGACTGCATTCGGATATCCCGTCGTCTTTTACCATCGCGCACTTCGACTACTCGCG

TTCGGTACAAGCTCGATCGTTGTTGATCAACACTAAACCAAAGAAAGAAAAAATGTCTAC

GTACAGGACGATGCTGACTAGCGCACTTGTGCTACTAGTTGTGGCTAGCGTAAGCGAGTG

CCGGATAAACAACCTACCCACGCGGTGTATGCCAGGGCTGCTGGAAGACGCACCGCCAAA

GGTGCGAGAAGCGTGTTTGACTTTGTCCACCATCCGTACACTTTCAAACGCCATCGAAAC

ATTTATCCAAGATAAACAATATCCGGTGCCTATGCCTTACACCAGAATGTCTGATGGATT

GGTTGATACAGCAATGCAAAATGATAAGCGCCAAGATCTGGACCACGTATTCCTCAGATT

CGGTCGCAGAAGACGTTAAATGCGACGCCTTTTTAAAATATGCTTAAGATAACTTCCTTC

TTCTTCTCTTAGATCCTAAACAACTTTATTATATTTGTGTGTCATATATAATAAAAAATA

TATAGTTATTGCAAATAAAAACGTAGACTACTATCTACACCATAAAAAAGAAAACTATAC

GTTAAGGTTAACATTTCGATCATTTGTTTGTATTTATATTTTATTTATTACTCGCGTAAA

TTGTTTTGATTTACATAGCATACCCAGCAATGTAGAATTTTAGTACCTATATCAATAAGA

TCAAAATTGTTGTGAACACATTTTTTAAGACTTAATTTATTTTTATTTTTAATGTATTAT

TTGTTTACCTACCTCGTTAGGATTGAATATCTGCTCATGTTTACTTAAAATAACATACTA

GTCTGCTGTATTTTATTTATTTGTTTATTTATTTTAGTAAGAAAACATCTTAAAATGTTA

TATATATATTATTATTCTATAATTAATAATATTAATAGACCTTATTTGGTACACTATATA

GATTGAATTACTGTTAACCATAATTATTATTACGATTATTATTAT

>NPF Unigene0058658

CAGCGTAGAGACTTTCGATAGGTACTTATTGACAACAATTCAATACGTACTTACCGTTTT

AACAATAATAACTACATAGGTACCTATTCAAAGTCTACTGAAATCCGATATTTTTCAGGT

TTTAAAATTACATCCAACGTTTTATCCATGCATCAAAATAATAACTCTAATATGATCAAA

GTAATGGTTTACATTGGATTTGTCGTCTATGTCATGGCCTTTACCACAACGTGTTGCTGT

AATCCCGTAACATCTTCTGAGGTGGAAAGCATAGCTCGACCAACAAGGCCTAAAACTTTC

GGTTCTCCAGATGAATTACGTTCGTATCTAGACCAGTTAGGACAGTACTTGGCAGTAGTT

TCTCGTCCAAGGTTTGGAAAAAGGAAACCGACGTTTCCAATCATCCCGTCCGCTATCACA

ACACCGAGATTACAACAGTACATCGATCATCAACACATGCTTAACAATATTTTAAATCGA

GAAGACGACGAGACTTATAAATTGCAATACAAACCGGCTACTATAAGAAATTCAAAAGAC

TTGTATGACATGCTGTTTACGACACGTCATGAAAACGGAAATAATGACCGCCATCAATAC

TATTTAATGCAACAAGATTTAACGAATGCAGACATTGGTTTAGATTCAAATTAATTATCA

TTAAACTGTTTAAACTATATTAGTAGTATACTACTTATCTTATCTTATATATAAGATATA

ATGTATTTGTATTTCTTAAAAACTTTAGTCATGATGTACATACAGTATTGTACTAGCTAT

TAATAATATAAGAACTTTAAAATTTTAATTCATCACTATAAATAAAATCCGTTAAAAGCG

TTGGTATAGGTATCTGATGCCTATTATAGACGTTAA

>NPLP1 Unigene0027013

GTCCCGCCCGCAGATAAATGAGCGGGATTCGGCCGCGGAGACGTCACTAAGTATTCGCGC

CGCAAATCGCCGGTGGTACACGGGCACGGCCGCAGCATCACCGTCGTCGACGTCGTCTCG

AGCCGCACGCTATACGCAAGCACGCCGCACAAGACAGCAGCCGGCGCCCGATGAAAGCCG

CAACGCTCCTGGCCGCCTTGGCCTGCCTGCTCGAGGTCTCCCATGCATTCCCGTTCAATT

TCAACAAGAGGAATTTAGATTCTCTAGCCAGAACGGGACAGTTACCAGAGTACAAACGAT

CGCTAGCTTCATTGGCTAAAAGTGGACAATTACCCGAAAAATTGGTTCAAGAAAAACGGA

TGTTGGACGTGTTGGAGCAAGCAAGTGATGCAAACTCAAAGGCCATTCAAGAGCAAAGAA

ATCGGGTAGTAGAGGAAATCAAAAATTTGGCACAAAGTGGGATAGCGAAAAGACAATCAG

CCTCGGAGCCTGATTTCCGCGATGAATTTAAGTCGGACTTGAGAAGCATATTGGATCAAT

TTTTGGAAAAGTTTGGAACTACATACGATCCAGAAAAGGTAGAGCTGTTTTTGACCAACA

TGGCCGACGAAGTGTTCGAAAATCATGGTGTAGTTTCGTTAGACCACATCCGATTCTTGA

TCGAGACTGGTTACTTCCAACCGGAAATGCGCGAAGAAGATGATGTAAAACCAGCAGACG

GAGATTCGTCGCCAGACGACTATATCACTAAACGATTTATGGCTTCGCTAGCCCGGACTG

ATAACATGCCGTTTTGGTACAACAGTCCGAGGTACGTCGCCAAGCGGTATATTTCTTCAT

TGCTTAGACAAGGGAGATTGCCTTATGGCTTTCAACCCACCACGGAGTCATCGACGAAAA

ACCAAGACTGGAGCTTACAAAATGGCCAAAAACCCAAGAGGATGAGTGAAGGGACGTTTC

AAAACAGCGAAGAGTTTATACCCGTGATGCAAGGTAGCAAAAGCTTGCAGAGTATTATCG

ACGACTTGACACAAGAGCCTATGCAGAAGAGATACTTGGGTGCGTTATCACGCAGCGGTT

GGATGCCCAGAGGATTCGCTTCGACGTCGTCCCGGTCACCGGCTTCGTATCACTCGTTCG

GCAGCACCGGACAGAGTGGCTACGGAAAGAGGCACTTGGCCGCGCTTGCCCGACTCGGTT

GGCTGCCGTCCTTTAGAAACCCTCATTCGTTTTACATGCGAAACGGCAGAGGGTCTTCGT

TCCAGTGCGACATAAACAAGAGGACTTTGGCCGCCGCCGCCGCAGCCACCAACTCCGACG

GGAACGTCGAGAATACTGAGCGGCCGTTTGAAGAGCTCGGGTGGGACCACCAGCAGCCGT

CGGTTGCAACAGAACAGGCGCAAACAGACGACGGGATGAAGAACAAGAGGTATCTGCTAC

TGCCGGCCGTGGACAACATATTGTTGCGCAGGCAGTACCCTCATTTAGCGACAAATAAAA

TTTAATTTAATACTATAAGTTACACGCTTCGCCGACGTATACAATATTAAACGATAACGT

AATGTTTTAACAAATAATACATAATAATATTACTATAACATATTAATATAATATAACGTC

ATATTTACCTCGTCGTATCAAACACAATATCCACCACCCGTCCGGTACGGGGCCGGCGGC

GCACCGTTGTAGTACACAATAAATTATCGTCGCGATAAAAATAGTATTATGATAATGTGC

ACGATCGGACTTACCGGACGGGTAAGTACAATAATCGACAAAACCTTTTTATTGGAATTT

CTCAAATTATAAGACAATAATAACTATGTGTGTTTCTTAAAAATTAAATTATCGTACTTA

AAAATAATAATAATAATATGTAATGTCTGTGTGTTGACATATATTATTTAGCTTTGCCCC

TCGTTAAGAAA

>NPLP3 Unigene0044545

ACAGCGTAATTATGTGTGGACAATAATTGAAATTTTCGAAGGAGACCAGATGTGATGATG

TTGTTGATATCATTTTGATTTAATTGTCATGAGGATGCGCTGTGAATCAGAGATGCGTTT

AAATGAAGTAGGCTGGTGCTGGTGCTGCTGCAAGTGCTGGTGCTGCTGCGTACGAGTAAG

CAGCTGGGTAAGCGTAGCTGGCAGAATATGCGAGTGGAGCGGCGGCGAACGTGTTGTGGT

TTCTGACGAAGCTCTGGCTGCTTGATGCGATTGGCAGGGCAGCGGGCAAAGCACTGTAAG

CTACGGGTGCAGAGCTCAAGTATCCGGGTTGTGGTTGTGGTGCTGCGAGAGCGACGGCCA

TCAAGGCAAAGAATACGCAGAGCTTGAACATGTTGGCTGATTGGGCTGTTGGTTATTCGT

A

>OK Unigene0031395

GTTGACGATAGTGTCCAACTGCTGTCTTTTCACGAGGAACACAAGTCTGTGACTGGCACG

TACACGTATAATATAATATATTATATTATATTGCACATCCATCTGCACACTTTTATTATA

CATATAGCCGGCTTTCGACTGTGGTCCAAATTTACACGTACAGCACTCGAGGTGTTCACC

TATACAGACACTCGTCGTGAACCGACGACGAACACGTACGGCGATTTTATCGTTCAAATC

ATCTGTTGATTATTCCATCAACTGCCTTGCACACACTCAAAACATTTTCCCGATATAATT

ATTTATATCGTTATACCGTAATAATATATACTTGCGTATATATTATTCATTTTTCACTCG

ACGTCCCCAAACAGTCCTCAACGACGAAATGGCCCACTGCAGTACTCTGATCGTCATCAT

CGCCTCATCGCTTTGCGTACACACCATATTGGCCTACCCGACTTCGATCGAACGCGTTTC

CGGGGACAACAATTACCAGCCTCTTCGGAACTCTGCATCGATCGATCGCTTCATCGAAGG

AGAAAATATTCTTAGAGACTTGGAGATGTTGAGAGACCGCGTTGAATACTTCGCTAGGCA

AACCCGTCATATCAATTCGTTGGACGGCATAGGTTTTGGTCAGAACAAACGATTTGACTC

ACTCAGTGGCGTATCTTTCGGAGGCCAAAAAAGAAACTTTGACGAAATCGACCGCACCAA

TTTCGATCGATTCGTCAAGAAGAACTTTGACGAGATCGATCGCACTAATTTTAATAGCTT

CTTGAAACGCCCCAGCAAGATGCCAGCGGCCAATTTGGAATAGACGCCGAATCTGCAGCA

AGCGCGAACACACACGCACACCCAAACACACTTACATAATGATAACAATAAAAATAATAT

AATACATTTTATTCACTAATGTTCATATAGATCGACATTATACCGACATAGCTAAAGAAT

ATTATTATGTATAATTTAGGTATTATAAAAGAAAAACTCATATTGTCATTAGGTATCTAT

AATACTAGATTAGTTATTATATTATATTCCACTGCATAGCATTACGATATTACTTATAAT

TATTATTAATAAACAGATGTAGCCAATCACAATAGTATGTCATTATGTTACCTATAATAA

CGGTCATTTGAAGTCCGAATCACCTCATCTATTTCGTTGTTTGGAAAAATGTGCGCGTGC

CATTTTACCGGGTATATACCTTAAACCTATAATATGTACTGTAGAAATGGAATTGAATCC

GATAGTAAGCATTTTAAAAAGTAAATTTTATTTTAATTTTTGACGTGAATATGTATTAGT

CGTCTAATAAAAAAAATATTATACTTAAAATAACTATAACTAAACAATACATTATATTAA

ATTTTTCTATTTTTTTTTTTTCATAGTTCTGTTTGTAAAACTCATCAACTATAATATAGT

CATTACTATTAATTTATAACAGATAA

>PBAN/PK2 Unigene0026434

GAACTGTCGTATAAAAGTCTGAGTCGCTGTCCACAACGGTTTATTCATCCCAACGCATTC

AAAATAACTACAGGCATAATAAACACAATGTGCACCGTCTGCTTTTTGTGGTTTATTGCG

TGTTCTCTTTTGATGACAAATGCCAGTATTTTAAATGACTTACAAGAAGCACAAAAGTTT

ATGGAACAGTTGGACTACGATTATTCGGCAGTGGTTGCCGATGCTGCAGCAGCAGCGGCG

CAACAGAGGCCTATCTCCGACCTGCTGTGGTACGACTATGGCGGTAGCGTCGGGCGAGGA

GAAAGCGGCGGCGGTGGAGGAACAAGCGGCGCCAGCTATTACGGCACCGGATCTCCGGCC

ACTGGTGTGGCCACGGCCGCGTTTTTCGGGGCCGACAAGAGGGGCGGCACGACCCAAGCG

AGCAACGGCGGCATATGGTTTGGGCCGCGGTTGGGCCGCAGGAAACGTCGCGGTGGATCG

CCGTTTGGCGGCGGCGGCGTCGCGCAGTCGGTGGACGGGAACGCGATCGCGCCGGCCGCT

TCATCGTTGCAGGATTCGTTGGCCGCGGGTTCGACGTCGGTGGCGGCAGGACAGGCCGCC

GTCTCAAATTTGATCAACAATGTGCCGTGGGTGCTGGTACCGATCATCGATAATTCCTTG

TACAATCAGATTCAGATGAAGCAGAACTCGAGGAGCGGCAGGTCGTCGGAAGAGGACGAC

GACGACGACGCGGCTTCGCGGTCGCGGCATACGGCCAGGTCGCCGCCTTACTCGCCACCT

TTCTCGCCGCGTCTCGGTCGACAGGCAATCATGAATCAGCCTCAGGTCCCTCGGCTTGGA

CGCGAAACGTTGCTGTACCGGCGGGACGCCAGAAACGCGTTGTACCCGCAATCGAACGCG

ACGGTGCTCAGACAACAGCGACAGCAACAGCTGCAGCAGGCCTCGTCGCTGCAGGCGGCC

ACTGAATCGGCGGCGGCGCGAAGGCAGGCCGTAGTGTAGACGACTTCCGCGGGCTTCATA

ACGTGCTTATTATTGAACCGAACGATGGGACAAGATCGATATTCACACGTTCATTATATA

TGTTATATTATATAATATTATGATTTAGTGTAGTCGGTATTTTTAGTAGTGTAAAATAAA

AACGATAACGATAACGATT

>Proc Unigene0027851

GCCCTCACTATAAAAATCTGCACCCGAAGCTCGCGACTCGAACTCTTCCGATCGCACGGT

CAGTCGGCGCTCGTGGTTGTACCCATAGAAGACAATAAAAATATACAGCCTACGACAAAT

ATATACATATATTATTATTATCGTTATTATTACATTAATATACTACGATTTGGTTTTCTC

TCGTTGATCAAAGACGATGGCAAGTAAATTTTCCGTGTTATTTCTCGTCGGGTTCGTGGC

CGCTGTGGTCGTCGCCCCGTACATGATGACAGAGGCGAGATACTTACCCACCCGGGGAAA

CGACGACCGGTTGACCAGATTAAAAGAACTGCTGACGGACTTATTGAATTCAGGTGCGCA

GCCCAACTTGGACATGGAAAGACCATACGTGGAATTGGACGGTGATTTTAGCAGACTAAG

ACCTAGAGAGTACAACATACCTGAAAAGTCAATAATGGAACTATTCAACCCGACAGTACC

ACACCATCAGAGGCCTAGGTCTTAAAGATGCAAAACTAATTTTTAAATTAAATATATACA

TATATACATTATACATATACTAACTGTCTTAAGTCCGTTTGTTTTTTCCATTTAAATTAT

TTTATCGCTTTTTCAAGCCAAATAAATATTATTATAATTACCTATACTATTTTTCGTCAT

AAAATAAAAATGTAGACTGTAGGTATTACTGCGTGAACAATCTA

>PTTH Unigene0022074

GCATGTTGTCTTGGGCAGTATCTCGATTTGGACATTATACGCGTTGGTGTCATGCGTGTC

AGTCTCATGTCTGTCGATGGCCAAAAAAGTAAGATGGGAACTGGTGTACGACCCGATGCC

GAACGAAGATACGTTCGATCGGTTCGCCGCCGCCGACCACCAAGCGGCCGTCAACATGAT

ACAGCAACGGGACGAACAGCAACGGCAGCGGGACGAGCAGCAACAGCAGCTGTGGCGGCA

CAGGGACGAGCAACAGAAACGGCACCACCACCGCCAGCTACAGCAACAGAAGCGGGACGA

ATGGCAACAGCGTCGCAGCAGCAGCAATAGCGAGTCAAGGTTCGTCGATAGCGTGTCAGT

GGCCGCGTCCGAGGTTTGCAACTGTACGCAAAGGACGACGATCCACCGGCTGACCCAAAA

CCACTACCCCAAGGAACTCCTGTCGGTCACGTGCAGCGGAAACTGGTGCAAGACGGCCTC

GTACCTAGTGCCGGTGTTGTTGAAATCGAACACTCAGCCAGTGGAAAACCAAGAAGACTT

GCCGGACGAGCTTCAGCAGAACGTCAATCACTGGAAGTTTGACCCCGTAAGCATCCCGGT

TGCCTGTTATTGTTCAATTAAATAAACGATATTTTTGCCTTTGACCTATATTATACGTAC

ATTGTAATAGACCAACACGTGTAATACAATATTTTCACCGTTTCTTTTTATCCTGCATCT

CTCACCTCCTACTACTACTTCAACCTCCGCCCGGTGCTCTGTTTATATAATTCTACAATA

GACAAAGGTGGACGAATAAATGAA

>SIFa Unigene0052320

GAGTTCTCGACAAGACAACATATTCGCTCTTCAGAACTAAACACTTAAAGAAACGCCGCC

TAACTTCAACATGAACTTCAAGTGTACAGTAGCCGTTTTCCTGTTGATGGTAGTGTTGAT

GTTTGCCACGGACTCCACAAACGGGTTCAGAAAACCGCCTTTCAATGGTAGCATCTTCGG

AAAGCGAACAATTTCTTATCCGGAATATGAAAATCCTGGAAAAACTATTTACACCATGTG

CGAGATCGCGTCGGATGCGTGCCAGAATTGGTTCCCTGCAACCGTAGAAAAGAAATGAAG

AGCCCGCGCCATCATCTAGTAGAAAACAAGACGTCAACTTGCGACGTTTCAAATTCAGTA

GGAATTCATAGAAAATATGTAGTTGAATGAACCGTGTTTTTTGAATTTTTTT

>sNPF Unigene0022605

ATATATTATGACTGTTATTTAATACTTTGACATATATTTAAGAGAAATATTAATTTGTGG

AGTAAACAAATATATTTTTTTAGGGGAAATGTAAAATTTACATTAGAACTAAAGTGGAAT

ACAATCAACTACGCTAAAAAATCTGTAAATACTAAAATGTATACATAAGGTACAATTTGG

GTAGAAAAGCACTGTGGTGGATGTAAAATATAAACAAATTAAGGGTTTTACAATGAAGCG

AATGATTTTAAATAAGAAGAGTATAATATATAACTTCTGATGAAAGGCGCATTTAATTCA

CATCTGAATCGTTGTGGTATTCTGCATTCAAGGCGTTCTGATAGAGTGCTGGGTCGCTTC

GGCGTCCAAACCTTAACCTCAGCGACGGAGACCTCTGGTTTTTACGGACCATGCGATGTT

GATTGGGATCGATCAAAGAATTTCCCATGGAATCTATTAGATCCCTTTGCAACAAAATTT

CGTACAGATCTTTGGCATTCTCGTAGTCCATGTAAGATGGAGCAGCTGATACTATTGTAG

AGACTAGAAGAAGTGTACAGACCACGGCTGCGATGGATTTCATCTTTGTTTCAGTTGGTT

TTGATCCTGACTTACTGTGGATGTGGGAGACTTTGACGTAGTCGTGCGATGATCGTCGCG

ATCGGCAGCAGTGGAATGATACAATATTATAATACCGGTGGAGGTCCGCCCGATCGCCT

>TK Unigene0033673

TAATAGAATGTTTTTTATTTATTAACGTTATAGATATTAACTACTAAATTAGTCATCTTA

AACAAATTTAAATAGCAAAACTCATATTATAATATAGTAGATATTAATGTCATGCTACGG

TATTATTATTAAAAAAAAATAAAATCGAATTCAACGCATTTCTCTTTCTTCGTCGAATTT

AGCTTCCACCAAATTTCGCTCTCTGTCGGAGAGTTCCGACTTCAGTTCGTCGATGATCCT

GTACAGGACATATGCCGGTGACAATTGGCCGTCCTCGTCGACGGCGTCCTCGTGGTCTAG

CCACTTCTTGCCGCGGACGCCGTAGAACGCGTCCACGGACGGAGCCTTCTTGCCTCGCAT

ACCGAAGAACCCGACCGCGGAACCCTTGTTGTTGCCGTAGTAGTCCTTCTTGCCCCGCAT

GCCCATGAATCCCATGCTGGCCCTCTTGAAGCCCTCCGCTGGACCGCCGCCGTACACTGT

GGCCCTGCCGTCGAACATCGGACGCCGGAATACCATGGCCCGTTTGTCCAGGTCGACCGC

GGCGGAAGTCTCGTCGGACGTGCCGCCGCCACCGCCTCCACCGCCCTGGTCCCGGTCTTT

TTTGCCCCGCATGCCCATGAACCCCATGCTGGCGCGCCGATCGACCGAAGGGTCGGCCAA

TACGGCCGCCGCCAGTGCGGCCAGCGCCACGAGGCCCACGTTGATTTTGTGTGGCATTTA

TCGGGCTCTGCTGCACGCCTGGTTGACTAGTGTCCGGCAAGGCGTATATGTTATCCCGCA

AAGAGTTAAATAATTATTAAATCACAAGCAG
