# Supplementary material for: Identification and Characterization of Neuropeptides and Their G Protein-Coupled Receptors (GPCRs) in the Cowpea Aphid Aphis craccivora
Source: Front Endocrinol (Lausanne). 2020 Sep 17;11:640. doi: 10.3389/fendo.2020.00640 (PMC7527416; doi:10.3389/fendo.2020.00640)
Supplement: Supplementary file 2 [file Data_Sheet_2.docx]

Supplementary material 2 Amino acid sequences of precursors of the predicted neuropeptides and protein hormones from *A. craccivora* and prediction of their mature active peptides. “*” represents incomplete N-terminus or C-terminus; the signal sequences (predicted with Signal P) are underlined; and the mature neuropeptides are indicated in bold. Mono and dibasic cleavage sites are marked in green color, amidation signals in yellow, cysteine residues responsible for the formation of predicted disulfide bridges in blue, sulfation sites in purple and pyroglutamination sites in grey.

>Adipokinetic hormone (AKH) precursor Unigene0027719

MRSLLLLAVFMLCACIVVG**QVNFTPTWGQ**GKRNAPASDECKSMDTLIYIYKLVQNEAQRIAMCERMSITS

>Allatostatin A (AstA) precursor Unigene0032333

MHHSCCMWILVIATAVWTDAMAGPEDKVGIKSQQAQQQQQSDIMQTMADGHPSMHMTSPAESYFNDPLVPLGYLAKR**AHKQYGFGL**GKR**LYRQYEFGL**GKR**STSKQYGFGL**GKR**AAHKQYEFGL**GKR**ASPSFYSFGL**GRR**ATPQYNFGI**GKRVSQPSFLNVDDRESDYTYNDLSEERKR**TADDMGHGQRFAFGL**GKRGAGAEWEDGDGEGDDSAPMWHPAVRR**ARLQYGFGL**GKRADRDYDAAAGTEYADSLQLADDVAAADINN

>Allatostatin B (AstB) precursor Unigene0029473

MQNVLRRIAATLVILCPVIVFSIPESAIQASSIKSSQSEQDNSDYPRSFDEDQEVKR**AWRDLQNAGW**GKR**GWQNLKTTW**GKR**TQDWQNLHSSW**GKR**QGWQKLQGGW**GKR**GWKDMQSGGW**GKRFKDQPSTDQLSQFDEYLDKYEDENPNEAEKR**SWDNFQGSW**GKR**AADWTSFRGSW**GKRNPVDYMNEYSGYGDNDNYKAYIFPPGYNSYLPNFQAEYEK

>Allatostatin C (AstC) precursor Unigene0013997

MATQMGFISYGVILTLAVLTVLLPKLTTGNVIDQRVLQRELGENSPEIMSMGDKDDFREIEDPAINDYTIKTKGDDQNLEIALIDYLFAKQMMNKIRARTDSYRAQKKR**SYWKQCAFNAVSCF**G

>Allatostatin double C (AstCC) precursor Unigene0053841

MVVWNKIVLFLISLCMVALCDSTFQKPLDPFTLLRYDKRTPEDQPPNNEKNVSVEFDEYPVVVPKRTALLLDRLMVALQKAVDGNNSGNMKGYYPERSIPISGAPRPSPGMELQRR**NQQKGRLYWRCYFNAVSCF**K

>Allatotropin (AT) precursor Unigene0043447

MAVNNNIMVRLLVIEITFLILAVVNSYPAFEDNEFKHKNRDKGRSIR**GFKNMDLSTARGF**GKRTDHYVNLMPSDLFVDNKEDSFNQNIPLDVSLEKILKNKYNNFIEMLIDVNHDGYISREELLQSIDGES

>Bursicon alpha subunit (Burα) precursor Unigene0009955

MSTINQDFFKYLIVLAMCNMAFA**NNNGNGVVVTARSSDDCQVTPVIHVLQYPGCVPKPIPSFACTGRCSSYLQVSGSKIWQMERSCMCCQESGEREASVSLFCPKAKQGEKKFRKVTTKAPLECMCRPCTGIEESAVIPQEMSNYAADEPAINGHFSKSI**

>Bursicon beta subunit (Burβ) precursor Unigene0017703

MYTTQLFIISIAIFFVHNVLA**EDNPEECETLPSELHIIKEEFDELGRLQRTCNGDIAVNKCEGACNSQVQPSVITPSGFLKECYCCRETFLRERIIALTHCYDPDGVRLTSDKLATLEVKLKEPADCKCFKCGDFSG**

>Capability (CAPA) precursor Unigene0012156

MKNLQTQIAAALLLTLTFFFTHALRHGSEYSDEYKRDSNRDRR**ESVAGLIPFPRV**GRSSIDSALQMENFYNTQRELRSHKR**EGLIAFPRI**GRR**SDSKNTALWFGPRL**GRSVVVPENYDTSYLDSDTPTIIKTLIEMKSKNYGDEDDNLM

>Crustacean cardioactive peptide (CCAP) precursor Unigene0044769

MNPSILTLVWMSILVSLVQTVFADDVIMQKRYFDNDNPVAEPIRRKK**PFCNAFTGC**GRKRSDESMATLVELRSEPAVEEISRQIMSEAKLWEAIQEARLELLRQQRQNKAERMEMKPFPVGLRRKRRSFAAGDKC

>CCHamide 1 (CCHa1) precursor Unigene0034430

MHKSFVKIYVFVLIIWAVEKSDC**KQGAACLNYGHSCWGAH**GKRNVDGTNDLDTLLRYRMALFKKSVHKDSSMDPNPEQSQEDIPNYYNIFKHYSKINSMKTNNDDTIDTWSVEPSNHLPNGRSYYEDQVLDPRIEYKIMKI

>CCHamide 2 (CCHa2) precursor Unigene0056057

MYSITMPLRPQIATTCYFCMVLLAIVVIFTVDNGAAKR**GCAMFGHSCYGAH**GKRSFQMPMQQPARDWLPTNEEENQIDDAINKYFKIKPSSPHFTPFWQKMVQMYNERKNNLPSNDSNM

>Diuretic hormone 31 (DH31) precursor Unigene0016990

MFTGNMMVGASVACGLLIVILMCTIPASLSAPYPLLQGQNNAYLSSENDGDPEVMLELLARIGQNIMRANELENSKR**GLDLGLSRGYSGTQAAKHLMGMAAANFAGGP**GRRRRSDMLPKLLTP

>Diuretic hormone 44 (DH44) precursor Unigene0030947

MRILAIAWILVVVTWCCDSAVIYEPVVQNTRYYEPRIQDLELLDKNFFDMASIEKR**NGAMQGESPRSRPSLSIVNSLDVLRQKLMYEVARRHVDENQKVLSQNHQILKNL**GKRSLFPFLEVPRRF

>Eclosion hormone 1 (EH1) precursor c17200_g1

MTTSSKKIAFLAAALLLTAIVGYTAA**DMANVAICIRNCAQCKKMLGDYFEGSLCADTCVKFKGKMIPDCENIESISPFLNKLE**

>Eclosion hormone 2 (EH2) precursor c17200_g2

MNTTLKKIVFLATALALIAVIGYAAA**DMEDVGMCIRNCAQCKKMLGAYFEGPLCADACVKFKGKMIPDCENIDSVAPFLNKLE**

>Eclosion hormone 3(EH3) precursor Unigene0032823

MNTSAKKTVFLVAALVLIAIVGYTAA**DMEDVGMCIRNCAQCKKMLGAYFEGPLCADACVKFKGKMIPDCENIESVAPFLNKLE**

>Ecdysis triggering hormone (ETH) precursor Unigene0053622

MSGNLTIIGLVCLQILRVMSINEYPDKKVQNIWLTDMDDRQAASRIERSDQFETASDVIMKDASVYPKIVRR**GFAGEEFFLKASKSVPRI**GRRNNDIQESPKRSLSKDQVNMVEYWPYLQPNDINDLTRKHDFDLPYNCQQLDAKTIFLVDMYNFVCNDQFYCCAPAKRTIANSPNANSL

>FMRFamide precursor Unigene0034006

MLLCLLPVTLTLAALVTDGVADAAAADKRFALRPVDPLTRR**SAMEKNFMRF**GRAFDCSWTAPSASAVKRRDPSSAVGRR**VDSNFIRF**GRR**DSNFIRF**GRGEVYTPGDNKIPRR**HYDVDVDGLEVRF**GRSGGNIDRSPFGAALPPPPYDDRR

>Glycoprotein hormone alpha 2 (GPA2) precursor Unigene0030917

MDKFRWWKNAVMILMFFLAVLPSPLATG**SNTWQKPGCHKVGHTRKISIPNCVEFPITTNACRGYCESWAVPSPADTVMINPHQRITSVGQCCNIMDTENVEVNVRCIEGVRKLVFKSALSCSCYHCKKE**

>Glycoprotein hormone beta 5 (GPB5) precursor Unigene0022160

MVCVTVLFAVSSMIVWSSVAG**YNMLDCNRQLSTFRVSNTDVNGRTCSDEINVMSCWGRCDSNEVSDWRFPYKRSHHPVCIHAGQVLTEFVLKDCDEGVLPGTELYVFPQATTCKCHTCKSSEASCEGYRYRDFQFISTDEEV**

>Insulin-like peptide 1 (ILP1) precursor c15478_g1

MKISVNLSILLLTLIIKIVIA**NANLQLPPQQYCGSSLANIMQIVCKNKYNGPSHGK**KRNEIDSDLLDYKDLEDYNAVDYPYQSRQEAMLFMPTRIVRSSKR**TIIDECCRRPCLISELKGYCANQD**

>Insulin-like peptide 4 (ILP4) precursor c25074_g2

MKISLYLSVLLLALVIKIVTA**NVRLQRSPQQYCGSRLADIMRVLCKNKYNGPNGQ**KRNPIESDVWDYKDVEDYNAIDYPYQPKKEAMPYMPSRFQRTFKR**SIIDECCHRPCYLSELKTYCAN**

>Insulin-like peptide 5 (ILP5) precursor c20960_g2 (incomplete)

MNSAVVLVIVMLLASHVSSSPIINFSWDTPRHFCGSQLANVLALICSNGYNFHPASDDVTVPSRRRKRVIVEECCENTCTPKHLKSYCWENRRR*

>Insulin-like peptide 7 (ILP7) precursor c10146_g1 (incomplete)

*AGVSQSIIHRRKMGIPLITEECCYNPCTRRTLKEYCAPSQQ*

>Insulin-like peptide 8 (ILP8) precursor c15326_g1 (incomplete)

*SGIQFAVGSPMSNRYMNEPNQFCGQALADELAILCKGRYNEPKGRANQKGKRGIVNECCFQPCTRNYMKTNYCPPETEVPRMNFPMLGPQKKVDYLGYYFQLIRHVLGLNME

>Insulin-like peptide 10 (ILP10) precursor c15424_g1

MKLYIIITTLLSIESGIQFAVG**TSLYDQYMNGLHTFCGQTLTNELSLLCKGKYNNPQGTTNQ**REKR**SVADECCSRPCSRNYMKINFCQEPEEMKTDSVLLAPLEDVDYIGYYFELLGRILAPNYLRIRRSVSSTTHTL**

>Ion transport peptide-like (ITPL) precursor Unigene0031227

MSKYSVMGYEPALVLVATLMAIMAVSVVAVPAAHHLHHHSGGHHRMTSSNGVAGSSSPSLSGIDHPLSKR**SFFDIQCKGVYDKTIFARLDRVCEDCYNLFREPQLHSLCRKNCFTTDFFKGCLDVLLLEDEVEKIQKWIKQLHGADPVGVGA**

>Leucokinin (LK) precursor Unigene0012286

MVKIGLPWLLLVLMKLTNREIKSDEILSLQEVFDICQVEPSTEICDLLEKSSSLVDLNKLDLPEKRR**QKTVFSSWG**GKR**QSTYPYG**GKR**PAFSSWG**GKR**ASDKHGRPKQTFSSWG**GKRSELDGYDNGEILEPQMDKRDFNGIKQDKNNYRNKMTKGIHALFTIFSDWSRDPEEKKGIRYAGIKSMRR**SSDFFPWG**GKRSTGDK

>Myosuppressin (MS) precursor Unigene0026279

MSTYRTMLTSALVLLVVASVSECRINNLPTRCMPGLLEDAPPKVREACLTLSTIRTLSNAIETFIQDKQYPVPMPYTRMSDGLVDTAMQNDKR**QDLDHVFLRF**GRRRR

>Neuropeptide F (NPF) precursor Unigene0058658

MVYIGFVVYVMAFTTTCCC**NPVTSSEVESIARPTRPKTFGSPDELRSYLDQLGQYLAVVSRPRF**GKRKPTFPIIPSAITTPRLQQYIDHQHMLNNILNREDDETYKLQYKPATIRNSKDLYDMLFTTRHENGNNDRHQYYLMQQDLTNADIGLDSN

>Neuropeptide-like protein 1 (NPLP1) precursor Unigene0027013

MKAATLLAALACLLEVSHAFPFNFNKR**NLDSLARTGQLPEY**KR**SLASLAKSGQLPEKLVQE**KR**MLDVLEQASDANSKAIQEQRNRVVEEIKNLAQSGIA**KRQSASEPDFRDEFKSDLRSILDQFLEKFGTTYDPEKVELFLTNMADEVFENHGVVSLDHIRFLIETGYFQPEMREEDDVKPADGDSSPDDYITKR**FMASLARTDNMPFWYNSPRYVA**KR**YISSLLRQ**GRLPYGFQPTTESSTKNQDWSLQNGQKPKR**MSEGTFQNSEEFIPVMQGSKSLQSIIDDLTQEPMQ**KRYLGALSRSGWMPRGFASTSSRSPASYHSFGSTGQSGYGKR**HLAALARLGWLPSFRNPHSFYMRN**GRGSSFQCDINKRTLAAAAAATNSDGNVENTERPFEELGWDHQQPSVATEQAQTDDGMKNKRYLLLPAVDNILLRRQYPHLATNKI

>Neuropeptide like precursor 3 (NPLP3) precursor Unigene0044545

MFKLCVFFALMAVALAAPQPQPGYLSSAPVAYSALPAALPIASSSQSFVRNHNTFAAAPLAYSASYAYPAAYSYAAAPALAAAPAPAYFI

>Orcokinin (OK) precursor Unigene0031395

MAHCSTLIVIIASSLCVHTILAYPTSIERVSGDNNYQPLRNSASIDRFIEGENILRDLEMLRDRVEYFARQTRHINSLDGIGFGQNKRFDSLSGVSFGGQKR**NFDEIDRTNFDRFV**KK**NFDEIDRTNFNSFL**KRPSKMPAANLE

>PBAN/PK2 precursor Unigene0026434

MCTVCFLWFIACSLLMTNASILNDLQEAQKFMEQLDYDYSAVVADAAAAAAQQRPISDLLWYDYGGSVGRGESGGGGGTSGASYYGTGSPATGVATAAFFGADKR**GGTTQASNGGIWFGPRL**GRRKRRGGSPFGGGGVAQSVDGNAIAPAASSLQDSLAAGSTSVAAGQAAVSNLINNVPWVLVPIIDNSLYNQIQMKQNSRSGRSSEEDDDDDAASRSRHTAR**SPPYSPPFSPRL**GR**QAIMNQPQVPRL**GRETLLYRRDARNALYPQSNATVLRQQRQQQLQQASSLQAATESAAARRQAVV

>Proctolin (Proc) precursor Unigene0027851

MASKFSVLFLVGFVAA**VVVAPYMMTEARYLPT**RGNDDRLTRLKELLTDLLNSGAQPNLDMERPYVELDGDFSRLRPREYNIPEKSIMELFNPTVPHHQRPRS

>SIFamide (SIFa) precursor Unigene0052320

MNFKCTVAVFLLMVVLMFATDSTNG**FRKPPFNGSIF**GKRTISYPEYENPGKTIYTMCEIASDACQNWFPATVEKK

>Short neuropeptide F (sNPF) precursor Unigene0022605

MKSIAAVVCTLLLVSTIVSAAPSYMDYENAKDLYEILLQRDLIDSMGNSLIDPNQHRMVRK**NQRSPSLRLRF**GRRSDPALYQNALNAEYHNDSDVN

>Tachykinin (TK) precursor Unigene0033673

MPHKINVGLVALAALAAAVLADPSVDRR**ASMGFMGMR**GKKDRDQGGGGGGGGTSDETSAAVDLDKRAMVFRRPMFDGRATVYGGGPAEGFKRASMGFMGMRGKK**DYYGNNKGSAVGFFGMR**GKK**APSVDAFYGVR**GKKWLDHEDAVDEDGQLSPAYVLYRIIDELKSELSDRERNLVEAKFDEEREMR

>Prothoracicotropic hormone (PTTH) precursor Unigene0022074 (incomplete)

*HVVLGSISIWTLYALVSCVSVSCLSMAKKVRWELVYDPMPNEDTFDRFAAADHQAAVNMIQQRDEQQRQRDEQQQQLWRHRDEQQKRHHHRQLQQQKRDEWQQRR**SSSNSESRFVDSVSVAASEVCNCTQRTTIHRLTQNHYPKELLSVTCSGNWCKTASYLVPVLLKSNTQPVENQEDLPDELQQNVNHWKFDPVSIPVACYCSIK**
