# Supplementary material for: Identification and Characterization of Neuropeptides and Their G Protein-Coupled Receptors (GPCRs) in the Cowpea Aphid Aphis craccivora
Source: Front Endocrinol (Lausanne). 2020 Sep 17;11:640. doi: 10.3389/fendo.2020.00640 (PMC7527416; doi:10.3389/fendo.2020.00640)
Supplement: Supplementary file 3 [file Data_Sheet_3.docx]

Supplementary material 3 Nucleic acid and amino acid sequences of neuropeptide GPCR genes in *A. craccivora*

>Ac_A1 c25397_g1_1 ATGGACTACCCGATAGCCATGCCGCTGACGACCGTCTGGCCAACGGGCGCCGGTACGTAC

AACGGCACCGACATGGGCCCGTACGGCAACGGCACCGGCGACAGGGACGCGCTGTACTGC

GACGACACTTCCGGGCACGAGTCGTCGCTGTACCTGATATCCAAGATCCTGTACGTGATC

GTGTGCGTGATCGGTCTGGTGGGCAACACGCTGGTCATATACGTGGTGATCCGGTTCTCC

AAGATGCAGACGGTGACCAACATGTACATCGTCAACCTGGCCATAGCCGACGAGTGTTTC

CTGATCGGTATACCGTTCCTGATCGTCACCATGTCCATGGAGTTCTGGCCGTTCGGCAAC

GTCATGTGCAAGGTGTACATGACCACCACCAGCGTCAACCAGTTCACCAGCAGCATATTC

CTGATGATCATGAGCGCCGACCGGTACATAGCCATATGCCATCCGATATCGTCGTCCAAG

GTGCGCACAGCGTACGTGTCCAAGATCGTGTCGGTGACCGCGTGGACGTTCAGCATCATA

CTGATGATACCGGTAATCATGTACGCCAACACGATGGACAAGGGCAACGTCAAGAGCTGC

AACATCATATGGCCGGAGAACGAGCTGTTCAGCGGGCAGACAGCGTTCACGCTCTACTCG

TTCGTGCTCGGGTTCGCCATTCCGCTCATGCTCATATTCGTCTTCTACATACTGGTCATT

AGGAAACTGCAAACGGTGGGACCGGTGAACAAGTCGAAGGAGAAGAAGAAATCGCACCGG

AAGGTTACCAAACTGGTGTTGACGGTGATCACTGTGTACGTGCTGTGCTGGCTGCCGTAC

TGGATCACGCAGGTGGCGCTGATATTCACGCCGCCCAAACAGTGCCAGTCCAAGATCGTC

ATCACGATATTCCTGTTGGCCGGTTGCCTGAGCTACAGCAACAGCGCCATGAACCCGATC

CTGTACGCGTTCCTCAGCGACAACTTCAAGAAGAGCTTCCTCAAGGCGTGCACGTGCGCT

GCGGGCAAGGACGTGAACGCGGGCCTGCACCAAGAGAACAGCACGTTCCCGCGCCGGAAC

CGCGGCGGCTCGGAACGCGGCGGCGGCCGGACAGGACGGGCCACCACCATACTTTGCCAG

GCGGTCAGCGGCGGAACGGGCGGCGGCGGCGCGGGCGGTTGCTGCAAAGACGACGACGGC

GGCTGTTGCGGCGAGACGGGACCGCTGGTCGGCCGCGCCGAACTCGTCAGCAAGGAGAAC

ACGTCCACAGCGCTCACCATGACGTCCCGGTCCACGTGCAACGAGAACAACGTGCAACCC

GCCCAGTTG*

>Ac_A2 c19014_g1 ATGAACCCGACTGCTGGCATGCCCACCGCACTCGAGGGAACCGCGATGTACAGGTACGAG

ATGTGCTGGAACATATCGGCAGGCAACTTTCCGTTGTGTGTCAACAGCACTGAATTTAAT

GACTTCAACGAAACCGACGACGAGATCCTCCAGGTGCAGCGCATCGTTTCGCTGGCCGTG

CCCATACTGTTCGGCATCATCGTAGTGGTCGGGCTGCTGGGCAACCTGCTCGTGGTGATC

GTGGTGATGGCCAACCAGCAGATGCGGAGCACCACCAACCTGCTGATCATCAACCTGGCG

CTGGCCGACCTGCTGTTCATCGTGTTCTGCGTGCCGTTCACCGCCGTCGATTACATGCTG

CCGTACTGGCCGTTCGGTGACGTCTGGTGCAAAATGGTCCAGTACCTGATCGTGGTTACC

GCGTACGCGAGCGTCTACACGCTGGTGCTCATGTCGCTGGATCGGTTCCTGGCCGTCGTC

CACCCGATCGCCTCCATATACGTGCGCACCGAGCGCAACGCGTCCTCCGCCATACTCGTC

ACGTGGGTGCTGATCGTGCTGCTCGCGCTACCGGTGCTCGCCAGACACGGCGAGGTCAAG

TACACGTTCTCCAGCATCGAGCACACGGCGTGCATATTCCTCGACCGCGACCAGAAGACC

CGACCGGACGGTTACAACAAGCCGGCGTACCAGATATTCTTCTTTCTCACGTCGTACGCA

ATACCACTGGCAATCATATGCATTCTCTACGTACTGATGCTGATGAGACTGTGGAAAGGA

GTGAACCCTGGCGGGCAGCCACCGTCGGCTGAGAGCCGGAGAGGTAAGAAGCGAGTGACC

CGAATGGTCGTCGTCGTGGTCGCCATATTCGCTTTCTGCTGGTTTCCGATACAGGTTATA

TTGGTGCTAAAAAGCATTAATTATTATGAAATCACGGCTATCAGTGTTATTGTTCAGATA

GTCAGCCATTGTCTTGCGTACACCAACTCATGTCTGAATCCAATTTTATACGCCTTCTTG

TCAGAGAATTTCCGGAAAGCATTTAGAAAAGTGATAATTCCATGGAGACCGGAAGTAAAT

GTCCAAGGAAGATTTGCAAATGGAGATGCCCGGTCGATGGCTGTCACTAGGACGACTAGG

ACGACTAACAACGACATACTT*

>Ac_A3 c23632_g1 ATGCTGTGCACAGACTTGTCGTCCGACCGGCAGAGTTATAACTACACGTGGATAGCGGAG

CTGCGCAAACTGAACCTGACGCAGGAACACTTGCAGTACCTGCTCAACATCACCACGTCG

TACGATGACGACGACTTGGACGTCGGTTGCTACAACTGCGGCGGCGCCGTGAAGAGCTAC

TCGCTGCTGTTCCGGGAGGTGCACGGCTACGTGAGCCTGTTCCTGTGCGTGTTCGGCGCA

CTGGCCAACGCGCTCAACATCGCGGTGCTGACGCGCAAGGACCTGGCCGGTTCGCCGATC

AACCGGATACTGTGCGGCCTGGCGCTGGCCGACCTGGCCCTGATGGTCGAGTACACGCCG

TTCGCGTGCTACATGTACCTGTCCACCGCCAAGAAGGAGGAGTTCTCGCACGTGGGCGCT

GTGTACGTGTTATTGCACACGTACGTGTCTCAGGTGCTGCACACCACGTCCATAGCGCTC

ACTCTCGTCCTGGCCATGTGGAGATATGTAGTCGTAAAATTGCCCAACTCTATGCACGCT

ATATGCTCCGACCGGCGATGTACCATAGCCATCAAATTATCGTATCTATTACCGTTCATC

ATTTGCAGTCCAACGTTTCTAGTGTTCGAAATATTGGAAACGAGAGTGGTCGAAAACGGC

ACTGTGGCAACGTTATATCATTTGGGCTTGAGCACAATAGCAAGAGTGAACCATGAACTA

TTATACATGATACATCTGTGGACATATGCTGTGATCATCAAACTACTGCCTTGCCTAATA

CTAACCGTCGTTACCATATCTCTGATAAACGCGTTGAGTGAAGCATCTGAAAGGAAAGCA

AAGCGACTTCCGACCCAGCAACAAATGGCTCGCATAAGGAATATGAAACTCAAGAAAAGG

ATGGACCGAACAAGCCGTATAATGATTGCAGTATTGTTATTATTTCTTGCGACAGAATTT

CCACAAGGAATACTGGGTCTACTAAGTGGCATATTAGGGAGGGGTTTTTTTCGAACTTGT

TACAATTTATTCGGGGAGCTTATGGATATGTTGGCTCTCTTGAATGCATCATTAAACTTT

GTATTTTATTGCTGCATGAGCAAACAGTTTCGTGTAGCGTTTGGACAATTATTCAAAACA

CAGCCTAGCATTATGTTTAAGCCCAGCAATATTTTGGAGACATTCGTA*

>Ac_A4 c27545_g1 ATGGTGATCGAACCGGGCCTGATGGACATGCTGGGCAACTCGCTGTTCGACATGCTTGCA

CCAGTGTCGTCCACCGAGTCCTCGGCCGTAGGGTCCACCGTCTCCGCGCACGGATCCGGC

GACGGTTCCGGTGGTCGTGTCGACGGTTCAGGTTCGGCTGTTGATGACGACGAAGACGAC

GGTTCAGGAATGGGCGGCGGTTCCGGGGCCGGAGGTTCCGGTGGCATCGGCCCCGGCGAG

CTCTGGTACAGGCACAGCCCCGCCATGACGGCCGTCTACTGTTTCGCGTATACCATGGTA

TTCTTGGTCGGATTGGTCGGTAACCTGCTGGTGGTGTCCGTCGTATGCCGGTCACCCAGG

ATGCGGAACGTCACCAACTATTTCATCGTCAACCTGGCCGTGGCCGACATTTTGGTGCTT

GTCTTCTGTCTGCCAGCCACACTCTTGTCCAACATCTATGTCCCGTGGATATTGGGATCA

TGGATGTGTAAGATCGTGCCGTATGTTCAAGGTGTTTCAGTGGCAGCGTCTGTTTACAGT

TTAATCGCCGTTTCAGTTGACAGATTCCTGGCCATCTGGTACCCGCTCAAGTGTCAGATC

ACCACTCGACGCGCTCGGTACATCATCGCCATCATCTGGCTGGCGTCGACCACGATTACC

ATACCGTGGGCACTGTTCTTCGACATGGTGGCCATATTCAAGGACGCCCCAAACCTGGAA

CTGTGCCTGGAGGTGTGGCCGGACTACCTGGACGGCAACCTGTACTTCCTGCTGGGCAAC

CTGGGCCTGTGCTACGTGGTGCCCACGGTGGCCATATCGCTGTGCTACGTGATGATCTGG

GTGAAGGTGTGGCGGCGGACCATACCCACCGACAACAAGTGCGCTCGCATGGAGCGCATC

CAGCAACAGAGCAAGGTGAAGGTGGTGAAGATGCTGGCCATGGTGGTGGTGCTGTTCGTC

GCGTCCTGGCTGCCGCTGTACGCGATATTCGCCCGGATCAAGCTGGGCGGCCGGCTGGTG

CCGTGGGAGGAGGACTTCCTTCCGGTGGCCACGCCGATCGCGCAGTGGCTGGGCGCGTCC

AACAGCTGCATCAATCCGGTGCTGTACGCGTTCTTCAACCGGAAGTTCCGGCGCGGCTTC

ACCGCCGTGCTCCAGAGCCGCCGGTGCTGCGGCACCCTGCGGTACAACGAGAACCTGCAG

CACTCGGCGTCGGCCAGCGGCAACGGCGGCGGCAAGGCGTCGTCGTACTACATAACCAAT

CATCACGCGTACACCAAACGGCAGTCCAGCCAGGAGACGAACGTGTCGTACATATTCAAC

GTC*

>Ac_A5 c24582_g1 ATGGAGTTTCCGAGCAGGGACGCCAACCAGAGCGGTGAAGCGTTCTGGGATTACGATGGA

CCGGACAATTGGCCATGGCAAAAGTACCGGATGAGGTACTCGCCCGAGGTGACGGTGCTG

TTTTGCATCGCGTACACAGCCGTGTTCGTTGTGGGCTTTGTGGGCAACATGTCCGTGGTG

CTGGTGGTGTACAAGAACGTGAGGATGCAATCGTCGCCCACCAACATATTCATCGTCAAC

TTGGCCATAGCCGACCTGTTAGTCATCGTCGTCTGCGTGCCGTTCACCCTCATCGGCAGC

ATCACCACCGAGTGGCGTTTGGGACTGGTCATATGTAAGCTTGTGCCATACTTTCAAGGG

GTGTCCGTAAACGCATCAATAAACACGTTGATGGCCATCTCCGTGGAAAGATGCTTATCT

ATTTGTTACCCTATGAACCCTGTTGGCAAAGGCGTCTGTAAACGGGTCGTGGCCATCATA

TGGATCATTTCGCTCACAATCACTATGCCATGGGCCATATACTTCGATCTGCAGCCTATG

GAAGAAGGCAGCGATAATCAGATCTGCCTGGAGTCGTGGCCGACCGTGGAAAGCGGCAAC

CTGTACTTCGTGTTGGCCAACCTGGTGCTTTGCTACGTGTTGCCGCTGACGGTGATCGCG

GTATGCTACATGTTCATCTGGCAAAAGGTGAGCCGGCGAAAGGTGCCCGGCGAGCCCGTG

CACAACGGCGCCAACATGGTGCAACGGTCCAAGATGAAGGTGATCACCATGATCATGTAC

GTGGTGGTGCTGTTCGCGGTATCGTGGCTGCCGCTTTACGTGGCGTTCTCGCTGATCAAG

TTCTGGCCGCTGCCACCGGCCGTCGAGAGCTACACGGTGGCCTCGCTTCCGGTCGCGCAG

TGGCTGGGCGCCGCCAACTCGTCCATCAACCCGCTGCTGTACGCAATATTCAACCACAGG

TTCCGGGACGGTTACCGGGCCCTGTTGTCCGGCAAGATTTGCCAAGCGTTCGACTACAGC

AATTCGGTGCGGTATCTGCGCGGCGGCGGCCGAGCCGGTACCGCGGCCGCGTTCAAGCGC

AACAACAACGACTACGACAACGGCGGCGGCCGCGACCGCAACCGCAAGACCATCGGTGCC

ATATACGTGCATGCCACCCGT

>Ac_A6 c25283_g1

ATGATTTCCGCCCTGGACGTGGTCGCGTTCGGCAACGACAGCTCGCCGCTGACGGTCACC

GGCAATGACACGGCGGGCGGCGCTGGGCCCTCGTCCAACGACACGGCCGCCAACGGCGTC

ATTCAGTTCCTCGGCGACGACCTGTCGTTCCCCGATTACATACGCACCACGTGCATGGTG

GTGTGTGTCATCATTCTGGGCGTCGGAGTGGTCGGCAACATGATGGTGCCGATAGTCATA

CTCAAGTCCAAGGACATGCGCAACTCCACCAACATATTTCTGATGAACCTGAGTATCGCC

GACCTCATGGTGCTGCTCATATGCACGCCCACCGTATTCGTGGAGGTAAATTCCAGACCG

GAAACGTGGGTGCTCGGCGAGGAATTATGTAAAGCAGTGCCTTTCGTCGAGCTCACGGTG

GCCCATGCTTCCGTACTGACCATACTGGCCATCAGCTTCGAGCGATATTACGCCATTTGC

GAACCATTGCGTGCCGGTTACGTATGCACCAAGACCAGAGCAATGATCATCTGTCTGCTG

GCCTGGGGACTGGCTGCACTGTTCACTAGTTCGCAGTCCGATCACATGATCTCGGAATA

CACGCAAATGGACTACATCGACGGCACCAAGGTGCCGGTGTGCCTGACCAAGGCCAACAC

GTTCTGGCCGATAGCGTTCTTCGTGACCATCATCGGCGTGTTCTTCGTGGTACCGCTCTT

CGTGCTGGTCGTCCTGTACACGGTGATCGCGGTGCACCTGATGGCCGACCCGGGCACCAG

CTGCACGGACAGCGCGTGCAATCAGCGCGCCCGCCGACAGGTGGTGCTCATGCTGGCCAC

CGTCGTCTTGTCGTTCTTCGTGTGCCTGCTCCCGTTCCGCGTTTTCACCATGTGGATCAT

ACTGGTGCCGGAACACACGTTCCTGGACCTGGGCGTCAAGCACTACTACATCATATTGTA

CTCGTCCCGGGTCATGGTCTACCTGAACTCGGCCGTCAATCCGATCCTGTACAACCTGAT

GTCGTCAAAGTTCCGGCGGGGCTTCTGCAAGCTTTGCCGGTCCCAGTGCGGCGGCGGCGG TAGCGTCGACGACTGCTACGACAGCTACGACGGCGGCGGAGCTGGC

GGTTGCGTCATCGGGTGCGGCGGG

>Ac_A7 Unigene0031155 ATGGCCAACGGCAGCGGCGGTGGCGGGTTCGAGGCGCTTCAAAACGTCGCCCCCAACGTG

TTTCCGCCCATATACATATCGCCCGGGCACGGGTCGAACGGCACGAACGCCGTTAACTAT

AGTGAAGCCATCGAACGAGAATACGTCGAGGACTACGTGTTTCAGTCCGCTTTCTCGTTC

GTCTATCTGCTCATATTCACGTTGGGCGTGTTCGGCAACGTGCTCGTCGTGTACGTCGTG

TGGGCCAACAAGCACATGCGCACCGTCACCAACATATTCATCGTCAACCTGGCCGTATCC

GACATTATGCTCTGCGGGCTGGCCGTGCCATTTACACCGCTCTACACCTTCACCGGCCAT

TGGATGTTTGGTGAGATATTCTGCCATATAGTTCCATATGCCCAGGGCACCAGCGTGTAC

ACTTCCACGTTGACGCTGACGTCTATCGCCATCGATCGGTTCTTCGTCATCATCTATCCG

TTCCAACCAAGGATGACCATATGGACCACGGCACAAATAATCGCAACCATATGGATATTC

TCTTTGGTAGCCACACTACCATACGGCATTTATATGGCCAACAAGGAGATATATGGGAAA

GATTTTTGCGAGGAGACGTGGCCGCAAGAGACGTTTAGAAAGATTTTTGGTGCTATTACT

GCCATCCTACAGTTTGTTCTGCCATTCCTAATCATAGCATTTTGCTATATCCGAGTTTGG

TTAAAACTTAACGACCGAGCCAGATGCAAGCCGGGTACGTCGACGAAAAATGCAAGGCGG

GAAGAAGTGGAGCGTGAAAGAAAAAGCCGGACCAACAGGATGTTGATAGCCATGGTGACT

ATATTCGGTGTGTCTTGGCTACCGCTGACCGCCATCAATCTGCTTAACGATTTCTATCTG

CAGACAGCCACGTGGAAACACTATTACTTATTTTTCTTCTCCGCTCATGCCGTTGCCATG

AGCTCCACTTGCTACAATCCATTCCTATACGCGTGGCTAAACGAGAACTTCCGGAAAGAG

TTCAAACAGGTGTTGCCTTGCTGGCGCAACGGTTCCGGGTACGGTACAACCGGTGCTGAC

TTGGGACAGGGGCGCCGAGGCCGAGTCGGTGGTTACCGGTCCGAGCGAACGTGCAACGGC

AACGACACGTGCCAGGAAACGCTGTTGCCAACGTCCATTGTGCTGCCGTCCGGTCGAACC

ACCGCCACTACAGATTGCACCGGACTCGATCTCGTGGATGGATTGATGATGGGCGATCAA

GAGGACAATCAGGATGCAGTTGAAGTGATGTTGGTCGCGTACACACGCGATGACGGTATT

TCTGGTCGTTCTGGTAATGTACAGCAGAACTCCATTAAAACTCAAGTA*

>Ac_A8 Unigene0019560 GACAATTGTGCAGCACTAATCATTCAAGATCAGAAGTACTGTTACTTCCGGAAAGCAGTG

CACTTTTGGGCGCAACAGGCACTGCTGCCAGCGGTTGTTGCCGTGGGCGTGGTGGGTAAC

ATGCTATCCGTGGTCGTACTTACTAGAGAACCGATGAAAAGCTCTACCAGCACGTATCTG

ACTGCCCTGGCTGTATCCGATTTGTTTTATTTGTTATTTGTGTTCACCATATCATTTGAG

AACTATCCATGGATCGTCGAAGCCGATTACTACATTTATTGGAAGTGGTATCCTTACGGG

CTGTGGCTTACTGACGCTGCTAGCAACACGTCGGTGCTGTTGACCGTGTCGTTCACCGTC

GAGCGGTACATAGCCGTGTGCCATCCTCTGCGCGGCCGCATGCTGTGCACCGAGTCCCGG

GCCAAGCGCGTCATCCTGATCGTGGCCCTGTTCTGCATCGCGTGCACGGCCACCACGCCG

TACGAGTGGCACATAGCCATCAACGCGGCCACCGGAAAGTTCCAGAAGAGCAGCACGGAA

CTGGGCCGGAACGACATCTACAAGAAAGCGTACAACTGGTTTTGCATCGTCACGTTCATC

TGTGTGCCTCTGCTGGTGCTCGCCGTGCTCAATTGGTTCCTGATCAACGCGGTCAATCAG

AGCCGCCGGAACCGGACGCGGCTCACCTGCCAGGGGAACATGGTGTGGAACAGGCAACGG

CAGGAGAACAAAATGACCATGACGCTGATCGCCGTGGTGATCATGTTCTGCGTGTGTCAG

ACGCCCACGGCCGTGATGATGCTTACCGCGTCCGTGTACGAGCCGCCCGAGAAGACGCCC

GCGTACTACGTGAACCGCGGCCTGCACACCATATTCAACTTCCTGATGGTGGTGAACGCC

GCTTCCAACTTCATGCTGTACTGCGCGATGAGCCGCAAGTACCGTCGCACGCTCATGATC

ACGTTCATGCCGTTCCTGGCGGCCCGGCACGCGCGCAACGCTACGCTCCGGTCGTCGGTC

AGCTACCCGCGATCCGGCACCATAGTCCGGCGGAACACCGAGGTGACGCAGATGTCCGAC

ATCACCGGCGTGTCGTCGTCCGCCGCCGCCGCCGGGAAACGCGAACTGCTCGTGCGATCC

GGCACCAACCACGGCAGACACGCCGCCGCCGCCAACAACAACAACAAGCAACAGCAGCTG

ACCGCCACGGCCGTCCTC*

>Ac_A9 c13254_g1

ATGTCAAGCTCAAACCAATCAGTAGGATTCGAACCATGGCTAAAAGAGAACCCAAAAATT

ATGTACGCTGCGGCGTTCATGACGTTGCTTATTATGTGTATTGGAATTATAGGTAATAGT

TTAACTATATTAGTAATACTAAAAAGTCCACGGATTCGAAATGTAGCTTCTACATTTATC

ATCAGCCTAGGTGCAGCTGATTTGTTATTTTGCATAGCTGTATTACCATTCAATGCTTCA

AGATTTTTAAATATTAATTGGGTTCAATACCCAGAATTATGTTCAATTGTACCATTTTTA

CAGTATGGAAATATTGGAGTATCACTGCTCTTTATTACTATGATCACCATAAATAGGTAT

ATAATGATCGTTCATTCAAGCTTGTATAGTATAGTTTATCGTCCGATATGGATAACTTCT

ATGATAATGCTTTGTCTAATTATTTCATTTGGCATGCTGATACCTACCCTTTTAAGTAAA

TGGGGTAAATTTGAATATGATCCAAAGTTGGGTACGTGTTCAATAGTATCTGATGAATTT

GGACAATCTTCGAAATCTGCTCTTTTGGTCACTGGATTCATCATACCATGTATTGTAATT

GTTTGCTGTTACACTGGTATATTTTTGGTTGTTCGTAATTCAGAAAAACGAATGCGAAGG

CATCAAACATCCACTCAAAGTGATCCAACAGCAACAAATATGCAGTCCATAAGAAGAAAA

CTTAGTGAATGGAGGATAACTAAAATGGTGTTGGCTATATTTTTGTCTTTTGTACTTTGT

TATTTGCCAATTACAATCACAAAAACCTTAGATCCAGGTGTTCAATACCCAGCTCTTCTC

TTGATTGCATATATTATGATTTACGCATCAGCATGCTTCAATCCAATTATATACGTCATA

ATGAATAAGCAGTATAGAAAAGCATTTAAATCAGTTCTAAATTATGGATGTTGTGAAAAA

GCTACCAAACGAGTACCTGTTTTGAGATCCATATCGAAACGCCTACAGCCACACGGTAAC

TTGACATCATCAACTGTCATGCAAACATTGCAAACAATAAGCCCGGGT

>Ac_A10 c21429_g1

ATGGACGAGGAAAACTACACGGCCGTGGTGCCCACTTACCTGTTCGATTTCAACGGCACT

GCGAACGGCGGAAACGACACCGAAGGCTACCTGAACGTGACCACAGAGATGCCTGTACGG

TACGCGCGACCCATGTACGGGTTCGTCATGCCGTTCCTGTTGCTGGTAACCATCGTGGCC

AATACGCTTATCGTGGTCGTGTTGTCCAAACGGCACATGCGCACGCCCACCAACGTGGTG

CTCATGTCCATGGCCCTGTCCGACATGTTCACGCTCCTATTCCCGGCCCCGTGGCTGTTC

TACATGTACACGCTGGGAAATCACTACAAGCCACTGTCGCCGGTCGAGTCATGCTACGCA

TGGTACGCCATGAACGAGGTCATACCCACTCTGTTCCACACCGCGTCCATCTGGCTCACC

CTTGCCCTGGCCGTTCAGAGGTATATATACGTGTGTCATGCACCAGTGGCGAGGACATGG

TGTACAATGCCGCGAGTTCTTAAGTGCGTCGCCTGGATATCAGTAATGGCATCATTACAT

CAATCGACCAGATTTGTTGACAGAACATACGAACCAATTAAAATATCATGGAGAGGACAA

GACTCGGTTGTCGTGTGTAGAATGAAGCACGCATATTGGGTTGAACATTGGGTCACACTA

GACGTATATTTCACGCTGTACTATGCGTTCCGTGTGATCTTCGTACACACTGGCCCTTGC

ATATCTCTCGTTGTATTAAATCTTCTACTGTTCAGAGCAATGAGAGATGCGCAATTAAAA

AGAGACAAATTATTCAAAGAGAACAGAAAGAACGAATGCAAACGTCTAAGAGATTCTAAT

TGCACAACATTGATGCTCATTGTCGTCGTGACGGTTTTCTTAATGACTGAAATTCCGTTG

GCTGTAGTGACGGTGCTTCACATAATATCAAGCAGTATCAAAGAAATACTTGACTATTCG

GTGGCCAATCTGTTGGTGTTGTTCACCAATTTCTTCATAATCGTCAGTTATCCTATCAAC

TTCGCCATTTATTGCGGAATGTCCAGGCAATTCCGGGAAACGTTCAAAGAACTATTTATC

AGAGGCTCGGTGCAGATAAACCGTAAACATGGGGCCGGCGGCAGCTCCCGTTACTCATTA

GTCAACGGACCCAGGACATGTACCAACGAATCGCTTTTG*

>Ac_A11 c44582_g1

TGCTGTGTAATTTATATGATGGTTGGCGTTCCTGGAAATCTTATCACGATCATCGCCTTG

TTTAGATGCAAAAAGGTGCGTAACGCTACAGCCGTATTCATCATCAACCTCAGCGTATCC

GATTTGAGTTTTTGTTGCTTTAACTTACCTCTGGCTGCCTCTACGTTCTGGTATAGATCG

TGGATACATGGTCAATTGCTTTGCCGTCTCTTCCCGCTAGTCAGATACGGGTTGTTGGCC

GTATCACTATTTACCATTCTGGCTATAACCATAAATCGATATATCATGATTGGACATCCA

TCGCTCTACCCAAAGATGTATAAGAAATTCTATTTGGGCGTTATGGTTACGGTGACTTGG

GTAGGTGGTTTTGGGCTACTGATTCCTACGTGGCTTGGAAAATGGGGACAATTTGGTTTA

GATGTGACCGTTGGCTCGTGCTCCATACTACCAGATTCTGTC

>A12 c18209_g1 ATGGACCAGCTGCAGTCCGCCAACCAGTTCGACGATCTGCCGCACAAATGGGTGGACGTG

GTGGCCGACTACTTGCGGGGCTTCCGGAACGACACGACCGACTTCAACCGCCCGCAGCTC

CGGTCGTCCGTCCGGCACACGTATCCGGCGTTCGTTTGCGGATATAGCGCGCTCATCATG

GCCGGCGCCCTGTGCAACGCGTACGTGCTGGCCATCGTGGCCCGGAAGCGGTTGTACGCT

ACCGACCCGGTGTATGTGTACGTGGCCAACCTGGCCGTCACCGGCATCGTCGAGTGCGTT

TCCGTGCTGCCCATATCGCTGATGGTGTTGCTGGTCCAAAACTGGATATTCGGCCGGTTC

CTGTGCTTTTTCCTTCCAATGCTCCAGGATGTTCCTACTCATGTAATAATGTTGACCTTT

CTTCTAATGGCTATTGATCGTTACAAACATCTCAAACATCCAAATAAAATGAGACTGCCG

CCTTTAGCCTGTACATTTGGATGTTGGATAGTGGCATTTTGCATTGTATTGCCATATCCC

GTTTACACTGCTTATTTAGATTTGGGTGCATACATAAAAGTACAATTTGAAGGAGTTGGA

ATTTGTGCAGTAAACATGGCCGATGATATGCAAGACTATCTTCGGAGTTTATTTGTCTTG

ACATACTTGGTTCCGTTAGTGTCTATGGGCTACCTTTACTCAAAAATGTCAGAAATTTTG

CGCGACTTGATGAATCTTCCCGCTGTATTTTATTCTCAAGATTTAACACCAAGAAGCTGT

GAAATTCCATCACGTTTGAACGAAGTACAGTCGACGGTCAGCGACTCTGATGACGAGGAA

ATCGATGGATACAAAGAGTCAAAGACGCAAAAATATTTAATATCAATGATCATTTCGTAT

GCTGTGTGTTTATGTCCGCTAATGATTTTGAGATTGGCAAAACTTGAAGTATCTGAAACG

TACGAGAACAGTAGACACTTCGATTTAACGTTCATGATATGCGTTTGGCTGGCGTTCGTA

CCAACAGTTACCACCCCGTTGCTGTTTGTAGCATGGAATTCGGACAGTAGCACCAAGGAT

CGGATCAGGAGTTATTTTAAAAGGACAAAGGTCACGGCGGAACAAGCGACCAGGACTGTG

TCGACGGAGGGTCTCGCAGCCCGCAATTCGATTTATACTGTGCAGGAGAGCATTCCGGCT

*

>A13 c26478_g1

ATGGAAAACGGTAACGGGACTTCCGGAGCCGATGGCGGTGCAGCTCACATCTCGCCCTAT

TGCGGTGATATGCTGGTCGACTTGCACGACGTGTACGTCCACTACCACGGTTACGCCAGC

CTTCTGGTGTGCGCATTTGGTTCGGTTGCGAACGTGCTTAACATCGCCGTGCTGACACGC

AAGGAAATGGTCTCGCCCACCAACGCCATACTCACTGGGCTAGCCGTGGCTGACCTGCTA

GTCATGGTCGAGTACGTACCGTTTGCTTACCACATGTATCTCCGACCGACCAACTACCCG

CGCGCCGACCGCTTCTCATACAACTGGTCCCTGTTCGTCTTGCTTCACTCGGACTTTTCG

CAGGCTTTTCACACTATATCAATATGGCTGACAGTCACTCTGGCCGTATGGAGGTACGTG

GCAGTGGTACATCCACAGCTCAACCGGATATGGTGCCGGATGGAGACCACTCTATCGACG

ATCGCGTTGGGCTACGTGGTGTGTCCGATCATCTGCATACCCAGCTACCTTTCGTTCAAC

CTGTTCTCCCGGGTGGAGACATTGGACGCGAACGGCAACCGTCCGACGGCTGTGCTACAG

ACAGCGCTGCGGCGGGCCAATAACGGCACCGACGTTCACAACGCCCACGTCATCGGTGGA

GGCAGCGGCGGAAGTGGGGCAGTCGGTGGTGGAAGTAGCGCTGGAGCTGTAAATGGCGGC

AGCGGACCGCTACGCAACGTCACGCTTTACTACGTCAACGTCAGCGACCTGGCCACGTCC

ACGTACCTGGCTGACATAAATTTTTGGGTGTACAGCGTCGTCATCAAGATCATACCGTGC

GTGGCGCTCACGGTGCTCAGCCTGCGGCTGATATGTGCCTTGCTGGAGGCCAAACGCCGC

CGGGCCAAGTTGACGGGCAGCGGCCGCAAGTCAGCCGACAAAGAGCGGCAGACGGATAGG

ACCACCCGGATGCTGTTGGCCGTGCTCATGCTGTTCCTTATCACCGAGTTCCCGCAGGGC

ATACTCGGCCTGCTCACACTGTTACTTGGCAAGCGGTTCTTCCAAGACTGCTACCAGAAC

ATGGGCGAAGTGATGGACATGTTGGCGCTGGTCAATTCGGCCATAAACTTTATACTGTAC

TGCGTGATGAGCCGTCAGTTCCGCAACACGTTCAGCCTGCTCTTCTTGCCGTCCTGGATC

TCCAAGGTTGAGTCGCAGGCTTTGTCGCACGGTAACCCGACCACCACGCAAGTCACGCAA

GTC*

>Ac_A14 c12924_g1

GTGATCGTCAGCTGCGTGCCGTTTACGTCTACCGTGTACACATTCCCGACGTGGCCTTAC

GGACTGGCCGTTTGCAAGGTATCGGAGACGGCCAAAGACGTTTCGATCGGCGTGTCGGTG

TTCACGTTGACCGCGCTCAGCGCCGATCGGTATTTCGCCATCGTGAACCCAATGCGAAAG

TTGCACGCGTCCATCGGCGGACGGTTGGCCACCCGGTTCACGCTTACCGTGGCCGCGGCC

ATCTGGGCGGTGGCCGTGGTTTGCGCCGTGCCCGCCGCCCGGTTCTCGTACGTCCGCCAG

TTCCGCGTGCACAACGTCACCCTGTTCGAAGCGTGTTACCCGTTCTCCGAGCACCTCGGG

CCCGCCTATCCCAAGGTCATGGTGACGGTCAAGTTCCTCGTCTACTATGCCGTGCCGCTC

GCTGTCATCGCGTGCTTCTACGTGCTCATCGCTCGGTACCTGTTGCACACCACCAACAAC

ATGCCCGGCGAACTACAGGGACAAATACGTCAGGTACGAGCTCGAAAAAAAGTCGCTAAA

GCTGTGCTGGCCTTCGTTTTGATGTTCGCCATCTGTTATTTACCTCATCACGTTTTTATG

CTGTGGTTTTACAACTACCCCAAGTCAACGGACGAGTACAACACTTTCTGGCACGTATTA

CGCATTGTTGGATTTTGTTTGAGTTTCATAAACTCTTGTATCAACCCTATTGCTTTGTAT

TTGGTGAGCGGCACGTTTCGCAAGCATTTCGACAAACAGTTATTCTGGTGGTGCATGGCG

TCTTCAGCGGTCACCACGGAATCCAACCTGTTCGTGATAAAAAAGAACGGAGCCACGAGT

CGAGATACAAAAATCACAGAGTTCATAATGTTGCCGTCGATGCATTCAACTGCAAACAAA

AATAAAAGTAATAAGACTTTTACTATGATAGCGAATACCGAA

>A15 c24117_g1

ATGGCAGCTTCAATAGCCATTTCCGGCAACTATACGGACGTTAACGACTCGGACAACGTTTACGAG

CCCTATTCGAACCGACCGGAAACGTACATCGTTCCCGTGGTGTTCGCCATGATATTCGTC

GTGGGCGTTTTGGGCAACGGCACTCTGGTGTTGGTGTTCATCAGGCACCGGAGCATGCGC

AACGTGCCCAACACGTACATACTGAGCCTGGCGCTGGGAGACCTGTTGGTCATCATCACG

TGCGTGCCGTTCACGTCCACCGTGTACACGGTCGAGTCGTGGCCGTACGGTGAGCTTATA

TGCAAGCTCAGTGAAGCCACCAAGGACGTATCCATCGGCGTGTCCGTGTTCACGCTGACC

GCGCTCAGCGCCGAGAGGTACTGCGCCATCGTGAACCCCATCCGACGGCACGTGTCGTCA

AAGCCGTTCACTCTGATGACGGCCGTAGCCATATGGATACTGGCCGTGGTTCTGGCCACG

CCGTCGGCAACGTTCTCGCACCTGGCAACCGAGTCGATACCCAACACGAACGTGACCATC

GAGTACTGTTACCCGTTCCCGATAGAGCTGGGCAACGGTTACGCACGGGGTATGGTCATG

TTCAAGCTGCTCGCGTACTACGTGGTGCCGCTGTGCGTGATCGGTTGCTTTTACCTGCTG

ATGGCCCACCACCTGATGGTATCCACGCGCAACATGCCCGGCGAGCTGCAGCACGCCGGC

CAGTCGGGCCAGATCCGGGCCCGGAAGAAGGTGGCCAAGATGGTGCTGTCGTTTGTCGTC

ATATTCATGGTCAGCTTCCTGCCGTACCACGTGTTCATGGTGTGGTTCCACTTCAATTCG

AACTCGCGAGACGAGTACGACGACTACTGGCACGCGTTCCGCATCGTCGGCTTCTGCCTG

AGCTTCATCAACTCGTGCGTGAACCCCGTGGCGCTGTACTTCATCAGCGGCGTGTTCCGC

AAACACTTCAACCGGTACCTGTTCTGCTGTTGCCCGTTCGCCCGGTCCGGTCCGGCCACC

GTCGAGTCCACCATCCAGGACATCAACCTGACGCACGTCAACAGCACGTCGTGCAGGCGT

CACAACTCCGTGGTCACCAGCCACGCCACCACACTCGGTCACGCCACC*

>A16 c27080_g1

ATGCATTCATTTACACTCGGATCAAACAAAATGTGGGTGAAATGGACGTTGATTACTATG

ATTGCGTTTTCGACGACAGGCGTAACCGAAGGACACATTAACATGACAACTCGACGAGAA

TCAACTATTCCACCTACTGCGATAGAAAACGACACCGAGTACGACGAAATGGCCAACTAT

ACGGATGAGGAATATCGCAATTACCTAGTCGAATACGTTACACCCCGAAAGTCCGAATGG

GTATTTATAGTCATGCACTCTATGGTTTTTGTTGTCGGACTTATTGGAAACGCTTTAGTA

TGCGTAGCCGTTTACAGGAATCGAACAATGCGAACGGTAACGAATTATTTTATCGTTAAC

TTGGCGGTGGCTGACTTTTTAGTGATCCTAATCTGCTTGCCACCAACTGTGGTGTGGGAT

GTCACAGAAACATGGTTCATGGGCACTATAGCTTGCAAATTGGTTTTGTACTTTCAGACT

GTCTCCGTAACCGTGTCAGTAATGACGCTGACGTCAATTTCTATCGACCGTTGGTACGCC

ATCTGTCATCCTCTAAAGTTTAAATCTACCACCAGCCGTGCCAGAACAGCCATTATTATC

ATATGGATCGTGGGACTCGCCTCAGATATCCCAGAGCTGTTGGTGTTGGAAGCCATTGAA

AAGACAAAACGAGTTTCGTCTATTTATCTGACCCAGTGCGAAGCTCTTTGGAGCCAAGAG

TCTGAAACTATCTACCAAATCGCTAAGACCATCATACTGTACATCTTGCCACTATTGCTC

ATGTCGGTTGCCTACTATCAGATTGTACGGGTGCTATGGAAATCCGACAACATACCAGGA

CACACAGAAACCGTACAAATGTTCAATGCCAATGCTTACAATGGTTTCAACCGGACGGCA

ACGATGGGGTGTACCAGCACAATGGCTCAAATAAAAGCCAGGAGGAAAGCGGCGAAAATG

TTGGTGGCAGTTGTGGTCATGTTCGCACTGTGCTTCTTTCTCGTGCACCTGATGAACTTG

TTGAGATTTACGGTGGGCATTCAACAATCGCAGGCAACAGCTCTAGTTTCAAACTTAAGT

CATTGGCTGTGCTATGCTAACAGCGCAGTGAACCCGCTGATATACAATTTTATGAGTGGC

AAATACCGTAACGAATTCAAACGGTTGTTCCTATGCTGGGGATCGCACAGGCAGAATCGT

CTGCGCCGTGCAGCGCACACTTCCCGAAGTGGCACGTACATTTGCCGATTCACGATGACC

ACAATGAAGACAGACAACGTGAGCTTTGGACTGTCGCCTGAAGACATTCAA*

>Ac_A17 c24908_g1

ATGGATGACTTGGTCAATAATCCGGAGTATTTCCTTAAACACTGGACGAACAATTTAACG

AGACACATTAGCGTACCGTCCGCACACGAAATGTTCAACGACACGACGGTGCGTAATGTC

AGCAGTTTATGGACGAAAAATGCGGCTGGGATGGAACAGGCGGCGGTGATCGATGAGCTG

ATCGCCGAGTTTTCGTCGTCGCAAATGAAATTCACCGAGACCAGAAGTATCATGTTGATC

GGCCTTTACGTGCCGTTGTTCCTGGTCGCGGCCATAGCGAATTCCGTGGTGATCGTCGTG

GTTATCAAATATCACTACATGCGGAGTGTCACTAACTACTTTTTGGTGAACTTGTCCATC

GCCGATCTTCTAGTGACATTCATATGTATGCCAATGGCCGTTGGACAATCGGTGACTGGC

TTATGGCTATATGGCGAGACTATGTGCAAATTAACATCGTATTTACAAGGCGTTTCAGTT

GGTGCAAGCGTTTTTACTATAGCTGCAATGAGTATCGACAGATATTTAGCAATCGAACAT

TCGATGTCATTCAGAAAGGTACTCAATCGAAAATCGACCATCTATGTCATACTTGCGTTA

TGGCTTGTATCCATGACTATATTCGGTCCAGTTTTATGGGTCCGTCAAACAGAATCTGTG

GAGTTAGGAGATGATCCAATATTAATTGATGCAGTCCATAGATACGGTTTGGCATGGTGT

ATAGAAGACTGGGGAAATGCACATGCAAAATCAACGTTATCTAAACACGTCTATGGTATT

CTATGTTTTGTGCTGGTGTATGCTACACCTGGATTTTTAGTGACTGGAGCCTACACATTG

ATGGGTCGAAGACTATGGGCAGTACGACCACCCTTTGATGACCAACAGGGCATGATAAGC

GTCCAACAGGTCAGAATGGTACGGGAGAGACGTAGAGTAGCAAGAATACTCTTTGTCTTA

GCAGTAATATTTGCACTCTGTTGGCTACCATACAACTTACTAACACTTTTTTTAGATTTA

GACATCACGCTTGATAAATTTGGTCTAGATCAGGAGTATTTAATGAAGTGGTATCCTTTT

ACACTACTATTGGGCCATGCTAATTCAGCAATCAACCCATTGTTATATTGTTTTATGACA

AGGAATTTTCGAAGAACAATTAAAGGTTTCGTATGTAATACTGGAATCGCTAAACCGAGA

AGAAGAAATCGATGCAAAAAGGGGTTGACCGAGAAAAGCACAACCAGTGGATACGGCTCG

TTCCGCAACCCACGTCGGCTGTGTTTCACCCTGGCGCAACTACGCCAAAACAATACAGTC

CAAACACGAACTGCAACGATATCAAACCTTGCTGTTCTG*

>Ac_A18 c25234_g1

ATGTCGACTCATACCAATGAAGATTTAGAAGACGAAATAAGCCTAACTGACAGGTTTGTA

AAATACTATCCGTTAGCTGTTGTGTGTCTTGGAAGCTTGGGAAATTGTTTATCAGTACTA

GTGTTTTTTGGAACAAAATTGCGAAAACAATCGTCATCGTATTATTTATCTTCGTTAGCA

ATCAGCGACACACTATTTTTATTAATACAGCTGATGCCTGTGCTATCCAAAGTTGGAATC

GGAATATACCATATGCATGGTTTTTGTCAGTTTTTTGTCTACTTAGCACAGATATGTAGT

TTCATTAGCGTGTGGCTAGTTGTAGTGTTTACATCAGAACGTTTTATAGCTGTTCGATAT

CCATTACATAGATCGGTTATATGCACAGTATACAGAGCTAAAATCGTACTATTCATATTA

ATCACGTTTGCATTAATAGTACATATACCTTATTTAGTAATTTCAACGCCTAACAGCACA

GTCTTAGAAAATAATTCAACAACAACAGAATGTAGCTTGAACTTTTCCTGGTACGAATTA

TATAAATGGCTAAACTATGCAGACGTTATGATGAATATGCTGATACCATTTTTCCTGATT

GTGATATTCAATAGCATGATTTGTCAATCTGTGTGTCGGTTGGCCAGAATAAGACGAACA

ATGACACTTCATCCATCTAGAAGAAGACAATCTACGTCACAAAACTCACAACACACATCT

CAAATAAAAGTTACAGAAATGCTTTTAGTCGTGTCTACAGTGTTCTTATGTCTTAATCTG

CCATCGTACGTATTCCGAGTATGGATGGTATGGGACAATACATCAACAAAATACAAAACT

ATACAAGTCATTGCAAATCAAATGTACAACACACATTTTGGAATAAATTTCGTGCTGTAC

TGTGTAAGTGGCCAAAATTTTCGAAGAGCTTTGGTTGAGCTTTGGAATAAAAGAAGACAT

CCCAATAAAAGATTAAGGGAAACTCAGGTTACAACAGTATTATCAGAATTCTCAAAAAGT

GGGGTTGGATCAAAACAAACTACTGTCAACGGAACTTGGAAGGAAGTACATGAACTTATA

CCAATTGCACACAGT*

>Ac_A19 Unigene0035849

ATGGACGGCGGTAGCGGTGTCGTCAACAACACGTCCAATAGCACGCTACTCGTAGACGAA

TATGACTACTCGGAGAGACCGGAAACGTACATCGTTCCGGTACTGTTCGCATTCATATTT

TTCGTGGGTACCGTTGGAAACGGATCTCTGGTGTTGATATTCATCCGGCACAGGAACATG

ATCAACGTACCGAATATATACATACTCAGCCTTGCGTTGGGCGATCTACTGGTGCTGATG

AGCTGCATACCATTCACGTCCACTGTCTACACGGTGCCATCGTGGCCGTTCGGGTTGACC

ATCTGCAAGGTGTCCGAGACCACAAAGGACATATCGATCGGCGTGACCGTGTTCACGTTA

ACTGCCCTGAGCGCCGACCGGTTTTTCGCCATCGTGGACCCGATGCGCAAGCTACACGCG

TCCGTGGGCGGCCGCCGGGCCACCAAGTTCACCGTCACTGTGGCCGTGACCATATGGTGC

CTGGCGGTGGCGTGCGCCGTGCCCGCAGCCACCAACTCGTACGTCAGGCAGTTCCAGCAG

AACAACGTGACGCTGTTCGAGGCGTGCTATCCGTACGCCGAGGAGCTGGGGCCCACCTAT

CCACGGCTGGTCGTTGTCATTCGGTTCCTGGTCTATTACGTGGTGCCCCTGTCTGCCATT

GCGTGTTTCTACGCCATGATGGCCCGGCACCTGATCCACAGCACCAGGAACATGCCCGGC

GAGGTCCAGGGACAGATGCGTCAAGTCCGAGCTCGGAAGAAAGTTGCCAAGACCGTGCTG

GCTTTCGTTCTGGTGTTCGCCGTGTGTTTCCTGCCTTATCACGTGTTCATGCTGTGGTTT

TACCTAAACCCCAGGTCCCAGGACGAGTACAATATATTCTGGCACGTGTTGCGTATCGCC

GGCTTCTGCCTGTGCTACAGCAATTCGTGCATCAACCCCATCGCCCTCTATCTGGTCAGC

GGTACGTTCCGCAAACACTTCGACCGACAGCTGTTCTGGTGGCTCGTCAAACCACCAGGC

GGTCAAATCACCGAGTCCAAGAACGGCTACATGCGCCGAAAGAACGGCACCCGAGAGAAG

GACCGAACCATCAACAACGATTCCACCACCATAGCCAATGTGCAGTTGAGCACTTTTACC

AGGCGGACCGCGGACACGAACCACACAAACACCACGACGGTGCTCATTTGTACCGGTCTC

AACGACGCTAACGCTATCATA*

>Ac_A20 Unigene0006642

ATGATTTTGATCGGGTTCCAGACGACCGTTTACCTAATGTATACTGCAGTGTTTGTCGTG

TCCTTGGTCGGCAACGGTCTGGTATGCTATGTGGTCTTGTCCTCAACTCAAATGCAATCG

GCTACCAACATGTTCATCGTGAATATGGCAGTCGGTGATCTAGTCATGACTTTGTTTTGC

GTTCCGTTCCCGTTTGTCACCACGTTCTTATTGGAATATTGGCCACTTGAGAACTATGTG

TGCCAAATCTTTACCTTCGGAAAAATTGTTGCCGTGATGGTCGGTGCATATACGTTGGTA

GCCATCAGTGTCAACAGGTATATAGCAATTATGTGGCCACTGAAACAAAGAACTAGAAAA

CACCAGGCTAAGTACATAATAGCATTAGTATGGACAATGTCTGTCATTACATCGTTTCCT

TTTTTACTTGCTACTTCATTGGATCAGAAATTTGACATTTACTTTTGCACTGACAAGTGG

TCGAGTGAATTTATTCGGCGATTCTTTAATGCAGCATTATTTTTACTGCAATGCTGTATC

CCATTCGCCGTCTTATTGTTTACTAATATTCACATTGGGGTTGTCGTGTGGGGTAAACTA

CCTCCTGGTGAAGCACAAAACTCCAGAGATATTAAAATGGCCAAGTCAATAAGAAAAATG

ATAAAAATGATGGCAACAGTGGTCATTGCATTCATCGTTTGTTGGCTACCATATGATATC

CTTTTGGTGTTGAGGGCGTACGGGATGTCGCTGCGCGCGTGGAGCAACCAGCCGTACGTG

TGGTTCGCGTTCCACTGGCTAGCCATATCGCACACGTGTTACAACCCGTTGATCTACTTC

TGCATGAACACCCGATACAGGGCCGGTTTCGTGTCGGCCCTGCGCAACGTGCCCGGACTG

GGGTACACGCGGGCCCAGCAACACGGTGACGAGCTGCCCGAGACCGGACGGCCGCGAGTC

GCGTCCGTGGTTTCGGTG*

>Ac_A21 c21731_g2

ATGTCTGAGAGCGACGACGAGCCGTCGGCCACAACGTGGAACGTCACCGCCACTGTGGCCGCGCGG

TGGAACGGCACCGAAGCCGTGGACGAGCTGTACTGTGGCGCCGACGGGTCGGCCGCGTTC

CAAACGCTCGTCTACCTGACGTACACGATCGTGTTCGTCGTATCGCTGGTCGGCAACGGT

CTGGTGTGCTACGTGGTCGTGTTCTCTGCGCAGATGCACTCGGTCACCAATTTGTTCATC

ATGAACATGGCCGTCGGCGACCTGCTCATGACGCTCTTCTGCGTCCCGTTCTCGTTCGTC

GCCACGCTGCTGCTCCAGTACTGGCCGTTCGGCAGCGACCTGTGCCACACCGTCAGCTTC

GCCCAGGCGGTCGCCGTGCTGGTAAGTGCATACACGTTGGTAGCCATTAGCGTTGACAGA

TATATTGCTATCATGTGGCCATTGAAACCAAGAGCCAGCAGACATCAGGCCAAATACATA

ATAGCATTAGTTTGGACTGTGGCTGTAATCACAGCGTTTCCAATACTTCTAGTTACCACA

TTGGAGCAGCCATCGAGTTGGCATCAGGAATGCGGACTTTATATTTGCAACGAGAAATGG

TCAAGTGAGAACGTCCGGCATTACTATAATGTAGCATTGTTAGTTCTTCAATATTGCATA

CCATTCGCTGTTCTACTATTTACTTATGTTAACATCGGGGTAGTTGTATGGGGCAAAAGG

ACTCCGGGTGAGGCTCAGAACTCCAGAGACGTTAGAATGGCTAAGTCGAAAAGAAAAATG

ATAAAAATGATGGTGACTGTGGTAATAGCGTTCACAGTTTGTTGGCTGCCATATAATATA

CTTCTGATCCTGTGGGACCACGAACCTTCGCTGAGCACGTGGAGCAGTCTGCCGTACGTC

TGGTTCCTGTTCCACTGGCTGGCCATGTCGCACACGTGCTACAACCCGCTGATCTACTGT

TGGATGAACACCCGGTACCGGACGGGTTTCGCGGCCGTTCTCCGCAACGTTCCCGGGTTC

GGGCGGTGTTTGGGCGGCTATTTGAGGGCAACACAAAACCAGTCGCACCGGTACAACAGC

CACAACGATCCCAGCCAGGCGGACGGCCTGCACCGGATCAACACGACGTCGTCGTTCGTG

TCGGTCAAGTCGCGGCTCAAGTCGTTCAACGGACGGCCCGCGGCGTACGGCCGGAACCGT

CAGAACTGGCACGAGGAGAGGCTC*

>Ac_A22 Unigene0038062

ATGGCCGCCGCTGAAATCGCAACCAAGATCGCGGTAAATTTCACAGAAAATCTCACAGCC

AATATCACGGCAAATGTTACCTCAAATGTCACAGTCAACGACACGTTACTACATGACCAA

ACGACCTCCGATCTTTATAAAGTGCCGGCCCTATTGGTCGTCGTCCTATCCGTGCTTTAC

GGTTCGATATCAGTGATAGCTGTGGCCGGAAACGGGTTGGTCATATGGGCCATCGTCACC

AGCAAGCGGATGCGGTCGGTCACCAACCACTACTTGGCAAACTTGGCGTTCGCTGATATA

CTCATCGCACTCTTCGCCATACCATTTGAGTTTCAAGCTGCCCTGCTGCAACGATGGAAC

CTACCGTCGTTCATGTGTGCTTTTTGTCCATTCATACACGTCCTTTCCATCACCGTTAGC

GTATTCACACTTACCGCCATAGCTGTGGATCGACGCCAAGCTATTCTCAATCCGTTCGCT

GCAAGGACATCAAAGACCCAATGTCTGTGCGTGATCGCATTGATTTGGATCGCCGGCCTT

GTGCTAAGTTCACCGATGGCGTATGCACAACGAGTGGTTTTTGTGTCCGAGGACTGGCCG

TTCTGTCTTAACGTCAATCTGTCTAACAACGTGATGCTCGTGTATAGAGCGCTACTAGTG

GTCGTACAATACGTCATACCGTTGTCAATCATGACGTGGGCCTATTCCGGGATAGGATTC

GCATTGTGGGGTTCGTCGGCTCCCGGAAACGCGCAGTCACAGAGAGACTTGAATCTGATG

AGAAATAAAAAGCGGGTAATAAAAATGCTTATCATCGTCGTCGCACTATTCACATTATGC

TGGTTACCCCTGCAAACGTATAATATATTACAGCATATATTTCCTCAAATCAACGAGTAT

CCTTATATCAACATAATTTGGTTCTGTTTCGATTGGTTCGCCATGAGTAATAGCTGTTAC

AATCCATTTATTTATTCAATATATAACGAAAAGTTCAAACAAGAGTTCAAAATGCGGTTA

GACTTCATGGCGGGAAAAAGGCGGCTGACCAGGGATCTGAGCGCGTTTTCCAGCGGCCGT

TTCGAGTGGCGCACCAATCACGTGAACACTCACGAACGAAATCTGAAGCATAGCTCGATA

GTCACCAACGACACGCCACTGATACTCACGCCCGACGCG*

>Ac_A23 c18353_g1

ATGGATGAAAATAATTACAAAATTGTATGCAATGAATCGCTGGATGAAAATATAAACATA

AAGAGTAATAATTCGATAGCAATAGATAGATGCAATGATTATAGTAATTATACTAATCAC

ACGGATTTGTATCTAGAAACTACATTTCTATATGATGTGCCCGTAAGCATAATTGTCCTT

CTGTCAATGTTTTATGGCACGATTTCTGTCATGGCAGTGGTTGGAAATGCTTTAGTAATA

TGGATTGTGACGTCCAGTAAACGGATGCACAACGTAACCAATTTGTACATAGCAAACTTG

GCATTAGCCGATATCGTTATCGGTCTTTTTTCAATACCATTTCAATTTCAGGCTGCTCTG

TTGCAACGATGGAATTTACCTGCTATAATGTGTCCATTTTGTCCGTTCGTGCAAGTACTT

TCAGTTAACGTGAGCATTTTTACCCTTACCGCAATTGCAGTTGACCGGCACAGAGCTATT

CTCAGTCCGCTCAGCGCAATACCGTCGAAATTCCGAGCAAAAATGTCAATAGCCACCATT

TGGGCGATAGCTTTTGTGCTAGCGACGCCAATGGCTATTGCTCTCAGGGTGCAATTTATC

GAATACGGAGATAGAGACGGCCGGAAGTTGGTTAAACCGTTTTGCTACAACGTCCGGCTT

CCAGAGCGCTCTATGCTATTCTACAGAATAACACTTCTGTTTGTCCAGTACCTAGTTCCC

GTTGTCATTATAACTGTTGTATATATGCGAATGGCGTTACGGTTGTGGGGCTCACATGCA

CCAGGCAATGCACAGGACAGTAGAGACGCAAATCTAATGCGGAACAAAAAAAAAGTCATA

AAGATGCTAGTTATTGTGGTTGGATTGTTTGTTTTATGTTGGTTACCATTACAAACATAT

AACGTACTTCAAGATATATTTCCATCAATAAATCAGTTTCGCTATATAAACATTGTGTTC

TTCTGTTGTGACTGGCTAGCAATGAGCAACAGCTGTTACAACCCATTTATCTATGGAATA

TATAACGAAAAATTCAAACGTGAGTTCCGCATCAAGTTGAGGCACTTCCGACGGGGTCGT

TTTCGTGGCAAATCAAACGCGAATCATGGTAGGTTTGCGTCGATGCGGTCCACCACGTTG

TCCGAGTGGAAAAGAGGATATTCAACACGGTGTACAGATCGAACGACGGCGAACGGCGTG

TGCGTGGATAGCCCACCGCCGAGAAGGGACGAGCTGGAAATGTTCGTGTGCAAATCGGGC

AAGATCACGTTGATAAAGTGCGGCAGTCGTTCAGACCTTGAAGAGCTGTGCCTG*

>Ac_A24 c21909_g1 ATGAACGGATCGGATTTTGGCGGGCCCAACTTGACCGATAACGGAACTGACGGTGTGGTG

TGGCCTGACAGCAATCAGTTCGAGTTGCCATGGTGGCATCAGTTGGCGTGGACGGTACTG

TTTGTGCCGATGATCATGGTGGCCACGGGTGGCAACCTTATCGTCATATGGATTGTCATG

ACCAACAAACGGATGCGGAACGTGACAAACTACTTCCTGGTGAACCTGTCCATCGCGGAC

GCCATGGTGTCCACGCTCAACGTGTCAGTAAACTTTAGTTACATGCTGACCAGTAACTGG

ACGTTTGGCACCGCCTACTGCAAGATCAGCCAGTTCGTTGCCGTGCTGTCCATATGCGCC

AGCGTGTTCACGCTGATGGCGATTTCCATCGACAGGTACATAGCAATAATTCACCCTCTA

AGGCCACGTTTGGGCCGGAAGACGACACTTATGATAGCCACCAGCATTTGGGTGGTAGGT

ACAATACTGTCCATACCAAACCTGATATTTTTTACCACGCACACGGAGCTGTTTCCAAAC

GGTGATCAGCGGGTCATATGCTACGCTGAGTGGCCAGACGGTATCACTACGAATAGCTTT

CAGGAATACGTTTATAATGTTTCGTTTATGATAATTACATATTTCATTCCTATTGGACTG

ATGGGTTTCACCTACGCTATGATCGGTCACGAGCTATGGGGAAGTCAGAGCATCGGAGAA

TGTACACAGAGACAGTTGGAACATATCAAGTCCAAGAGAAGAGTTGTGAAAATGATGATC

GTTGTAGTAACAATTTTCGCCATCTGTTGGTTGCCGTATCACATTTACTTCATTGTCACT

TCTCATATGCCGGAATTAACTACCTCGCCGTATATACAAGACATATATTTGGCATTTTAC

TGGCTAGCAATGTCCAATTCTATGCACAACCCTATCGTGTACTGTTGGATGAACTCCAGA

TTTCGTCAAGGATTCAAGCAGTTCTTTTCTTTCGTACCATGCATCAATGTAAGAACCGGC

AGTTTGATCCGACGGGAAGTTGTCACCAGCAGGTACAGCTATAGCGGATCCCCGGACGCC

CACTATCGGATAGTGCGCAACGGCACCGTTTGCATACCCTTGAACCATCTTTGCAACGAC

AACGGAAGAGGTCACCTGAAAGTGCCGGCGGCTCATTGGAGGCAGGCGAAAAAACACGGC

ATCGACAGCAGTGTTGGCGAAATGAGCAGCACCAGCTTTAGCAACGCTGTGACGGGAGTA

ACAATAGCCATGGCGAGTACGTCGACCTGCAACGAAAACAGTATCAACTGCAACGAGGAC

AGCTTGAAAACCTGTCTAGGAAAAAATGCAAATTCCGCATCC*

>Ac_A25 c19757_g1

ATGAACAATACGACGAGTGGATCATACATGGTCCAGCTCGACGACACCGCTAACGAGACA

GTAGAGGGTTATTTGCTCCGAACTCGAGGGCCCAAACACCTTTCGCTGAACATCGTGTTA

CCAATCACCATCATCTATGTATTCATATTCGTCACCGGAGTGATTGGCAATATCGCCGTT

TGCGTAGTGATCGTCCGCAACAACTTTATGCACACGGCCACCAACTACTATCTATTTAGC

TTAGCCGTATCCGATCTCACACTATTACTCTTAGGACTGCCCAATGATCTCAGCGTTTAC

TGGCAACAGTATCCATGGCCTTTGGGTGAAGTTCTTTGTAAATTTCGTGCTTTAGTTTCA

GAAATGACTTCATACACGTCAGTGTTGACCATCGTAGCATTTTCGATGGAAAGATATCTG

GCCATCTGTCATCCCCTCCATTCATACGCCATGTCTGGGTTGAAGAGAGCCGTCCGAATA

ATCGCAGTTGTTTGGATGATTTCATTTTTCGCCGCACTGCCATTCGCCATGTTCACAACG

GTCGACTATGTGGATTATCCACCTGGGTCAGGAGATCCGTTGTACGAGAGCGCATTTTGC

GCGATGTTGGATAAGAACGTTCCGACAGGAGTGCCTGTATATGAATTGAGCTCGCTGCTG

TTTTTTCTTGTACCGATGATGATCATTATTGTGCTGTATGTGCTCATTGGACTCCAGATC

AGACAGAGTTCTAGGCACTCGCTAGGAAAACAAATGCAAGGGAACGTGCACGGGGAGACC

AAACAAATTCAATCGAAAAAGTCAATAGTCAGGATGCTAGCTGCTGTCGTCATTGCGTTT

TTCCTGTGCTGGGCGCCGTTCCACGCGCAGCGGTTGCTCTACCTGTACGCCAAGGACTCG

CCGTACTACTTCCAAGCCAACGAATTGCTGTACACGATCGCCGGTTGCTTCTATTACTTC

TCGTCGACGGTCAATCCGATACTGTACAATCTTATGTCGATGAAGTACAGGAGAGCGTTC

CGGGAGACGCTGTGCGGATACTCGGGGGACCGACGAAACCGCATGTCCCGGGAACTGCAG

TCGAGCTTCCGGGACACGACCGTGCCCCTGAACACGACGATCAGCACCGCCGATTGCAGT

CGTAAGTCAGTGGTGAACCGGTCCACGAGGAACCTGCAACAGTCCGATGCACCGTATAAC

CATCATCATTATGCGGCTGCACCGTCGTCCGACGATTGCAGTAGCGGCCGTCCGCCCGCG

ACGGCCTCCGACGTGCTGGTAATGATCTCTCCGGTCAACGGCAATACGCAGTGCTACAAA

ACGTTGCTCAGGGTGACCGTGCAAGGCCCCGACAACAAGACCACCACCACCACGGAACAC

GGCAACTTCAACAGCAAGCTGCAGGGCTGCAACGAAACACAAACCGAACATCCGCATTGC

ACCGAAATGGAGACTTGCATT*

>Ac_A26 c23525_g2

ATGGAGACCGTCACTGTTGCCGAAGAAGAACTGACAACTATGAAGTACGTCAATGCGACG

CCTGAGTTTACAAAAGAAGAGATAAACTCGTTTTTCTTCTACGAAACCATACAGTTTACA

GTCTTGTGGATATTGTTCTTGTCGATCGTGCTAGGCAACGGAGCCGTACTGGTAGCCTTG

TCATTTAATAAAGCCCGCAAGAATCGAATGAACTTTTTTATCATGCACCTGGCACTAGCT

GACTTATTGGTAGGACTGGTGAGCGTGATGATCGACATGATATGGAGAACGACTGTCACC

TGGTCAGCTGGTCCGATTGCTTGCAAAGTCGTCAAGTACTTACAGGTGGTCGTCACGTAT

TCGTCTACATACGTCCTGGTCGCCCTCAGCATCGATAGATACGACGCCATCACGCACCCA

ATGAATTTTTCTAGCAGCTGGCGTCGAGCTAGAGCATTGATCGGATGCGCTTGGATATTG

AGTTTTGTTTTCGCCGTACCCAGTGTATTTATTAATGAAGAAACAATAATACAAGGTCGC

ACTCAGTGTTGGATCGAAATGTCACCATGGCAATGGAAACTGTATATAAGCATAGTGGCG

ACAACGGTATTCGTCGTGCCGGCAATAGTCATCAGCGGATGTTACGCTATTATCGTGTAC

ACGATTTGGTCCAAAAGTAAACTCCTGTCACCGGCCAAAAATAACACGCTTCAACGTGGC

GCCAAAAAACCCGAAGAACACGACATCAGAAGGTCCAGTTCACGGGGCATCATACCGAAA

GCTAAAATCAAGACCGTAAAAATGACATTCGTTATCGTATTTGTATTCATTCTGTGCTGG

AGTCCGTATATAGTGTTTGATCTGCTACAAGTGTACGGCTACATACCAAAAACGCAAGCG

AGCGTGGCCCTCGCAACGTTCATACAAAGTCTGGCGCCGCTGAATTCCGCCGCCAACCCC

ATCATCTACTGCTTGTTCTCCACTCACATTTGCAGATCTCTCAGG*

>Ac_A27 Unigene0021484

TGGGAAGAGTACGTGGACGGTACGCTGGTGCCGGTGTACCGTACCGAGGCTATCACCGTG

TGGCCGGTACTGTTTTTCGTCGGCACCATATCGGCGTTCTTCGTGGTGCCGCTGTTCGTG

CTGTCCGTATTGTACGTGATCATCGCCCGTCACCTGATGGCCAATCCGGGAACGGTGGCG

CCAAACACCAACCGGGCGGCGCTCCGGTACCGCCGGCAGGTGGTGCTCATGCTGGGCACC

GTGGTCGTGTCCTTCTTCATGTGCCTGCTGCCGTTCCGGGCGCTCATCCTGTGGATCATC

CTGGCGCCGCCCGACTACAACATCATGGAGCTGCTGGGCGTCAAGAACTTTTACCTGTTG

CTGTTCTTCAGCCGCATCATGTTGTACATAAACTCGGCGCTCAATCCCATCCTGTACAAC

CTCATGTCGTCCAAGTTCCGGGACGGGTTCCGGCGGCTGTGCGGCCTGCGCCGCGGCCCG

TGGGCCAACCGGCACCTGGGCCGCAAGGGCACGGTGACGACGACCTCGGCGCACGCGGGC

GGCTCGGCCAACGGCGGCACGACCACGACCACGACCACGGCCACGTCGAGCGTGAAGAGC

GACGGGGGAGGCAACGACCGGGCCACCGCGGCGGCCACGGCCAACATGTACGCCCGGATG

AAGCGTAACGGCGTCACCGTCGTTTCGGCGGTTGACCGGAGTGACCGGACAGCCGGAGGC

CGGCGCATCATCAACCGGTTGAAC

>Ac_A28 c25921_g1

ATGAGTCTATTCAACACATCGTACCTGATGGGCAACGACACGGCGGGCGGATGGGGTCCC

AGGTACTTTTTTACGTTCTACTCCGAGTTCGGCGACGAGCAGGCCGAGTCGGCTGTCGAG

GTGACAGTGTTCATGGTGATATTCGCCGCGTCCGTCGTGGCCAACGTGTCCATTGCCTGG

GCCGTGCTCAGGTACCGGGAGATGCGCACCGTCACCAACTGCTTCTTGCTCAATCTCACC

GTCGCCGATCTGCTGTTCGCCGTCACCACGCCAGCGCTGGCCTACGTCCGTGTCCGGCCC

GACTGGCCGTTCGGCGATTTTGTGTGCCGGCTGTTGCCTTATTCGCAGTTTGTTTGCGGT

TTCGTGCTGCTGTGGACGCTGACGCTGATCAGCATGGACCGGCATCGGTGCATCGTCGTG

CCGCCGTACCGATCGCAGCTGACGCCTCGCCGTGCCACCGTACTCACCGTGCTCACGTGG

CTGATCGCGCTGGCGGTGTTCATGCCAGTGCCGTTCTGGTTCCACGAGCAGGCCGTGATG

GGCGGCACAGCGGTGAACGTGTGCACGCTGGTGTTCCCCAAGAACGACACGTTCAAAATG

TCCATCGTGTTTACCGTGTCCGTGGTATCCTTGTCCTGCATCCTACCGCTGTCGCTATTC

GTCTACCACTACCAGAGGATATTTCACAAGTTGAACAAGACCCGACGGCGGATCGAGCAC

TCGGTCTCGCATCGGTCCACCGCCGTCCACACTGCGTCGCGGAACAGTCTGTCGCCGCCC

ACCAACGGTTCCACACCACAGGTTTTGGTGCGTCACGAAGAGCTCCGGTACAGGAAGCAC

GTGCGAGTGGTGCGCGTGCTGCTGATCAACGTGATCGTGGTGCTGGTGATGTGGCTGCCG

ATCACCGTCGTGATGTGCCTGATCTACGTAGACGGCAGCCGGGACACGGAGGACACGGGT

TACTTCCTGCGCTCACACCACTTCATCATGGGCCTGCTGTTCGCGCTGCTCAACACCGTC

GTCAACCCCATCCTGTACGGCGTGCTGTCCGAGAACTTCCGCAAGTGTTTCGCCCGGCTG

TGGTTCATATCGAAGCGCCGGCGGGCCATGCACAGGGAACTGTTGGACAACGCCAGCAAG

TGCGGCGCCCGGACGCCCAGCAACGGCCACTACAACTCGACCCTGCAACCGGGAAGTTCG

GCGTCCGTGGTCGAGCTTCCGGCCACCGCGATCGTGTCGTCGTCCGCCACCAACGAGTGT

TGG*

>Ac_A29 c23903_g1

ATGGAAGACCATATAGATGATAATATTTTGAGCAATTGTACAGCCATGGTGTTGTCATTA

GAATTTGATAAACCATCAAAAGAACTTGAATATTACAATTCCACCTCTCTTTTGGCAATA

TTGGGAGATATGTTAAAATCTTCTGATTTGACGATGTCCAAACGCACAATTGCCAGAGAC

CGATTACATGATTGCGTTTATCCTTTACCGGAACCAGTGCATAATATTTCACTGTGGCAC

AAGTTAACCTGGTCCACTGTTTTTATCGCAATGTTAATCGTTGCCATTGTTGGCAACTCA

ATTGTCATATGGATAGTGGCAGCTCACAGACGAATGCGAACTGTTACTAATTGTTACATG

GTCAGTCTAAGTATCTCCGATTTGTTTATGGCTTCGCTCAATTGTCTACCAAACTTTATT

TATATGCTCAACTCTGATTGGGCTTTTGGTTTGGAACTTTGCATGGTATCCAATTTCGTT

GCTTACTGGACAGTTGCATCATCAGTCTTTACGTTGGTCGCAATCACCCTCAATAGGTAC

ATGGCAATTGTCCATCCACTTCGGCATAGGAGGTCAAAGACGAGAACACAAACGGTGCTT

ATTCTCATTTGGCTAATCAGTGTTTTTCTAGCAATGCCATGCATTTTATATTCTGACATT

AAAACAAAAAGATACATGAATGGAGAACTAAGAAGGGCGTGTTACATACTTTGGCCAGAC

GGAAGGTACCCGGATTCTAAAACTGAATACATATATAATATATTATTTCTATGCGTCACT

TATATAATACCATTGACAGTGATGGCTGTATGCTACACAATTATGGGTAGAGAACTTTGG

GGAAGTAAAACCATTGGACAGATGACACAAAGACACGTGGAGTCAATCAAATCGAAGAGA

AAAGTTGTCAGAATGTTCGCTGTAGTAGTGACAATTTTTTTAATCTGTTGGCTACCTTAT

CACTCGTATTTCGTATACGCATACCACAATAAAAGCATAGTAGTCAAGACGTATGTGCAA

GATCTGTTCTTGAGTTTCTACTGGTTAGCAATGTCAAACTCGATGGTGAATCCGATAATT

TATTACTGGATGAATCCTAGATTCAGAGCTTATTTTAAGTTGATCATATGCTACTGTGGA

GGCTTAAGAAATCCTGACAAAGTGTCTATTAATCTAAATGTGATTCGAATGAACGGTGAA

TCTCAATATTACTTAACCCGATCCAAATCAGGTCCAGTATCGTTGGGAGTCCAACACTTA

CGACGTCCACCAGAAACCCAAGTATTATGTCTACCCAGAGGCTACAGCGAAGTAGTACGT

CATCTTCCAAAAACACGAAATAATCAAGAAACAACGGTGATTCAAAGTTCTAGGCTTCAG

AAACTACAGAAGACTAGAAAACTTTCTACACAGAAGCTTATCTATAATCAA*

>Ac_A30 c20833_g1

ATGGACGCGATGAACTCGTCGGCCGACAACTACGGTTTAACAACGGTCGGCAATCGGTCG

TCGTCGCCTTTCCCTGAGTGGAGCGGCGGCGGTAACTGGTCCTCACAAAATGAGACGACC

GCAATTCTATGTCCCTTGTTCGAGAGTACGGACGACGACGATTACAACCTGCTGTTCGAG

TTCGTCACCTACGGCGTGCTGCTGAACGTGATCGGCGTGTTCGGCATACTGGGCAACGTC

ATATCCATGGTGATCCTGTCCCGGCCGCAGATGAAGTCGTCCATCAACTACCTGCTGATC

GGACTGGCGCGGTGCGACACCGTGCTGATCGTCACATCCATGCTGCTGTTCGGGCTGCCC

GTCGTCTACCCGGCCACCGGACACCTGTTTAACTACTACTTTAAGGTGTACCCGCTGATC

GCGCCCGTCGTCTACCCGATAGCGATGATCTCGCAGACCGTGTCCGTGTACCTGACGCTC

ACCGTCACCCTGGAACGGTTCGTGGCCGTCTGCCACCCGCTCCGCGCCCGGTCGCTGTGC

ACGTACGGCCGGGCCCGGGCCTACGTGGTGGCCACCATAGCGTTCGCCGTCTTCTACAAC

GTTACCCGGTTCCTGGAGGTGACCGTCCAGAAGTGCATGCACACCGGGTCCAACCAGTAC

GTGTACCAGGTGTACCCCAGCGACCTGCGCAACGACCACAACTACATATCCATTTACATA

CATTGGATGTATCTGCTCATCATGTACTTCATACCATTTGGGTCGCTGGCCGTGCTGAAC

GCTGCCATATACCGCCAGGTGAGGAGGGCTAACCGAGAGCGACAGCGGCTTTCGCGGCTG

CAGAAGAAGGAGATCGGACTGGCCACCATGCTGCTGTGCGTCGTAGTAGTGTTCCTACTG

TGCAACGTGTGGGCGCTCATATCGAACGTGGTAGAGGCATTCTACGGCATCATCGTCGAT

CACCTGGTCAAAGTCAGCAATCTGTTGGTGACGATCAACTCGTCCGTCAACTTCGTCATC

TACGTGATTTTCGGCGAGAAGTTCAAGCGGCTCTTCTTCAAGCTGTTCTTCCCGCGGGGC

GTGTGGATGTGCGGCTGGCAATTGGCCACCGACGGCAGGGGCGGCCCGGGCTGCGAGGGC

GGCGGTGGTGGCCACGTGGCAATGGACGACAGCGAGGCCACGTGCAACGGGGCGACGGCG

TTCGAGTGCAGGCAGCTCGGCACCGGGACGGGCGGTTCTACGTCGTACACCGACCACTTC

GGGCGGTCTCGCCGAGGACGCCACCATCACCAGCATCACCACCATCATCACCATCACCGG

GACGGCGACCTCCTGGACCGGAATCAGATGGCTAACAGCGTCGGTGGATGTGGTAACAGC

GTCAACGGTACATCCGACGATAGGGAGCTCTGCTTGAAGACGCCGACCAACAGCGGTATG

ATGTGGGAACACTCGACCACTACCACCACGACCACCGTAAACGTACATCAATTC*

>Ac_A31 c25336_g1

ATGGTTTGCTCGCTGGATGAAACAATGAGTACATTTATGGAAGCCTGTAATGATACAGAA

ATGAACTCAACATTTAATTTCAGTTTGGAAGAAGTTTATTCTATTATGTTGGAACATAGA

CGTAATTCCCGAAATTTGGATAAAAGTACTGAGACTCTGCTGATAATTGTGTACTTGGGC

TTGATGATCGTCGGGCTGTCTGCCAACTTGACAGTGATTTACGTGGTTGCCAGACGCGCT

CAGATGCACACTTCCCGGAACCTATACATAGTGAACTTGGCCGTTTCCGACATGACCCTG

TGCTTGGTGTGCATGCCGTTCACGCTCACGTCGATATTGCGACACCAGTGGAGCATGGGC

ACCATCCTGTGCAAGCTGGTGCCACTGTTGCAGGGCACCAACATCATGGTGTCGGTGGGC

ACGATCACGGTGATCGCCATAGATCGATACTGGGTGATCGTCCGCGGATCCGCGCAAAAC

GAACGGCGCACGGTGTACGTGTCCATAGCTATCGTGTGGCTGATGGCCGTCTTGACCACG

TCGCCAGTAGCTTATTACCAGGTGGTCGAACCGCTAAAATTTCAACATGTAGTCATATAC

GAATCTTGTCGGGAAAAATGGCCTTCGACCGATATCAAAGTGGCGTATAATATAGCAGTA

GTGCTCATTCAAGCAGTACTTCCAGCCACTGTTCTATTGGTGGTTCATATTCGTATTGCA

GCATATTTACATGCACACACTGCTTCTCAAAAAGATTCGAGGCGTGCACAGCGAGAACTT

CAAAGAAACAAACGGACCACTCTACTTCTTATAGGAGTGGCGGTCGTCTTTACCGTGAGT

TGGCTTCCGCTGGCCGTCTTCTCGCTGGCCGCCGACCTGATGACCAAGCCGATAACAACC

AAACAGCTGTACGTGACTCTGGCAGTTTGTCATCTGACCGCCATGACTTCAGCCATCTCA

AACCCGATTATCTACGGCTGGATGAACTCCAATATCCGCAACGAGCTGTTTCAACTGTTC

TACACAAAAATCTTGAGACGGCGACCGGGAAACCGGTCCACGGCAACGGCCACGACAACC

ATGAGGAACAGAACCCGACCTTTGATCACGTACAATACTTCGAATTACATGCCTGGAAGT

CAGGAGACGTTTTCGAAAGGAGTCACTGTGCTT*

>Ac_A32 c23253_g1

ATGGATATGATGGACTCGGACGCAAACACCGTGTTGCACTCGGTCACCAAGGGAGTGCAC

GTCGGTCCGCCACCTGTCTGGCCCGGCATGGACAACGTGACGAACTCGTCCATGTTCGAT

GAGTCGAATCTTCCGTACGACATGAAATTCAACGAGGGCCACGTGGTCGCCATAGTCACC

TACAGCATACTCATGGTCGTCTCGGCCATAGGAAACATCACGGTGCTGACGATCATCCTG

AAACGTAGGCGCAAGGCTGGAACGAGGATACACGCGATGCTCATGCACCTGGCCATCGCC

GATCTGTTGGTAACTTTTTTGATGATGCCATTGGAAATAACGTGGGCATGGACTGTGCAA

TGGATTCTCGGTGATCCACTATGCCGCATCATGTCATTTTTCCGAATATTTGGATTGTAC

CTTTCCAGTTTCATATTGATTTGTATCAGTGTTGATAGGTACTTGGCTGTTTTGCAGCCA

ATGCGTTTATATCAGATGGATCGCAGGGGAAAGTTAATGATTGCAGTGGCTTGGATTGCA

TCAGTTATCTGTAGCTTACCACAGAGTTACATATTTCACGTGGAACGACACCCTAATGCC

ACATGGTACGAACAATGCGTCACTTATAACGCTTTTTCGTCCAAATTGCACGAGCTGGCT

TACTTATATTTCGGCATGTTCATGATGTACTGGTTGCCTCTAATCGTTATACTATTCTGC

TACGCTTCCATCATCATTGAAATATATAGAAGGTCAAGAGAATCGATTTGCGGTCAAGGT

ACAGATAATGTACGTCGTCTGGGATTTTTAGGACGTGCCAAAAGCAGAACATTAAAGATG

ACAATCATTATTGTCATAGTATTTGTAGTATGTTGGACGCCCTACTACATTATGGCTATT

TGGTACTGGACGGACCACAAGTCGGCGCAGATGGTCGATCAGAAGGTACAGCACGCACTC

TTTATGTTTGCATGCACCAATTCGTGTATGAACCCGATCGTATACGGAGCGTTCAATATA

CGTACAAGGAGGACGCTGGTGACCCAGGGTGTTGGAGAGTCTGTAGCTTCGGTGAGAGTG

GTCACATGGCACAAATTGACTGTGCGATCAAAAGCTAATCGAAAATCTACAATTGGCAAT

AAATCGACATTGTTCAAATCTGTGCATAACGGTAATACGAATGATGATACTCTGGTCAAC

GAGAATGTCACAGTTACTACGACGTTGTGTAATGATAATAGCGACATTAACGACAAAAAT

ATT*

>Ac_A33 c20484_g1

TGGCCGGGTCTGTACGGCCCGAAC

GGCACGTTCGACGGAGGCGGCGACGTCGACGGTGTCGGGGCTGACGGCGGCCTAGGCGGT

TTCGACGGCGGGTTCCGGTTCAACAGCAGCGTCAAACTGGCGCTGGACGTGATATTCGAC

AACCAGTTGAACGTGGACAAGGTGCTGGTGATGTACCTGGAGGTGGCCATCGTGGTCGCG

TACGGCGTGCTTTTCGTCATCGGACTCGTGTCCAACGGACTGGTGTGCTTCGTCGTGTTC

CGCCAGTGCGGCAAGAAGAACGTGCCCAGCCAGGGACCGTCTCCCCGCAACCTGTACATC

GTCAACTTGGCGTTCGCCGACATCATCATGTGCGTGGTGTGCATGCCGTTCACGCTGTGG

GCGCTCATGTCCCGCCGCTGGACCTGGGGTCTGATCATGTGCAAGACCGTGCCGGCCGCC

CAGGGCGCCAACATAACCGTATCCGCGTGTACCATAACAGCCATCGCTCTGGACAGGTAT

TTTACGATCGTCCGGAATCCCCGGGGCTCGGTGTTCCGGTGTAGCGTAGCCAAGACATTG

GTACTCATTTGGCTAGTGTCGTTCGCTGCCATGGTGCCTCTGCTGTTGTACCAGAACGTC

GAGGAGGTGCACGTCGGACCCATTCGTCTGTACGAGGCGTGCGTCGAGAAATGGCCGTCT

CGCGTGGCCCAACAGACGTTCACCGTCGGCCTGGCGGTCGCACAGTTCGTGTTACCGTTC

ACTACCATATCACTGATCCATCTGAAGATATCGTCCTATCTCACAGTGCATCTCAAACAT

CCGACTATCCCGTCCAAGGAGATCAACTGCAGACGAGTGCGGAGGGAGCTGAGCCGGAAC

AAGCGGACCATGTGGATACTGTCGTCGATCGCCGTGGTATTTGCGCTCAGCTGGCTGCCG

CTGACGCTGTTCACATTGCTCGTCGAGTTCCAGCCGCACCTGATCGACTCTTCGGACTCC

CTGTACAAGGCTTTCGCGATCGTGCACATGATGGCCATGTCGACCACTTGTACCAACCCG

CTACTGTACGGCTGGCTCAACACCAACTTCCGGCGGGACATATCCGGTATATGCCGGCAG

ATGTGGTACTGCTGCGGTGACCAGAAACCGCCGCCCAGGCGGAGGCGGTACCCATCGGTG

GCCACCGATTACCGCGGCCCGAACGACCGGCGCATGGGCATGGGGTCCGAGGCGGCCGGC

GGATGTGGTGGCGGTGGCGGTGGAGGCAGTGGCGGCGGTGGCGGCGGAAGCCGACGGCAC

CAGATGCACCACGACCCCGAGACCACCGGCATGTCGATGGTGGTCAAGAGCGACCGGAGG

TTCAGCACCAGCTATCTGACCACACTGACCACGGTAACGTCCAGGTCGCAACCGGGTTCT

AGCATGGGACGCGTCGAGAACGTC

>Ac_A34 c23906_g1

ATGCTGTCTGAAGAAACTTTAACAAACGACACCTTCTACAACGACAGCATTAGCTACGGC

AATAACTACAGTTCACACGATGATCATCAGTGGGGCCCGAGGAGAGACTCGCTACCGGTT

GTCGTGCCCGTCACGCTCATCTACACGCTGATATTTGCCACAGGAATCGTGGGCAATGTG

AGCACGTGCATAGTGATCGCCCGAAATAAGTACATGCACACGGCCACCAATTACTATCTG

TTCAGTCTCGCCGTGTCCGACCTGCTACTACTGTTGTCCGGATTGCCGCAGGAAATTTAC

CTAACATGGTCCAGGTATCCTTATGTGTTTGGAGAGATGTTTTGTATAGTCAGAGGGTTT

GCTGCAGAGACATCGACGAACGCTACTGTACTAACAATCACGGCGTTTACCATTGAAAGA

TACGTTGCAATATGTCATCCGTTTTTATCACACACAGAATCCAATCTGTCCAGAGCATTC

AAATATATATTAATCATTTGGGTTGTAGCGTTAACACTTGCGTTTCCTCAGGCCATACAG

ATGGGACTTGTGTACGCTCGTACACAGGACGGCGCTATCATACCCGAGTTGTCCACGTGT

GGGCTGGCCAACAAGCTGCCTTACGCGTTCGAACTGTCTTCGGTACTATTCTTCCTCGCC

CCGTGGACGCTCATCATCGTCCTGTACATACTCATCGGCCTCAAGCTATACAAATCCAAA

CGCGACTCGTCCAGGACAACGTGCTCGGCTCACTGTCGGCACACCGCATGTCTGAGCTCC

ACCAATACCAGAGCCACCGGGCGCGTAGTCAAAATGCTGGTGGTCGTGGTTGTAGCTTTT

TTCGTCTGCTGGGCGCCGTTTCAAGCGCAAAGACTGGTTGCCATCTACGGATACGCGAAT

CACGAGGATCACTCAAGCACTTCGATAATCGATGCAAACGTTTATCAGGTGCTGAACTAC

GTATCGGGAATCTTATACTACTTGTCGGCTACCATAAACCCACTTCTGTATAATCTCATG

TCCTACAAATTCAGGACCGCGTTCAAAGAAACTTGGAGATCTTGCAAAGGAGCCAGTGGT

ATAAGTCCCGATCACAGTATGCCCAACCTAGACTACAAATGGCAGAGACACCACAGCCAT

AGCAGAAGTTTCAGCAGTAGTCATTTTGACGAGGTAAATTCAATGAAACGACTGATGGTG

GCTAATAAATCCTGGCAACCTACGATCGTAGCAATCAACGAGACCAGTGACACGGGCACG

TCGTCGGCCGCGGAGAAGACATTAAGGTTTCCACATAAAATGATTTTTAGCAAACAAGAC

GCTATCAAAGAAAACGGCACTGTGATGGAAAACGGAATAACCATTGGTGTGTTGACCAAC

AACAACAGGACAACCACGATGCTGATGACGCCGAAT*

>Ac_A35 c23752_g1 ATGTCACTGCAGAATGAGCTGTACATCGATGGCGACGGTAAAGGTAACCAGTTGAATTTG

ACAGCGGAAAATACATGGGACAACGACTACATAATGCCTAAACGGGATCCTCTATACATA

GTCGTACCGATGACCATAATGTACTCAGTAATATTCGTGACAGGTGTAATAGGAAACTCG

ATCACTTGCATGGTGATCGCCAAACACAAATACATGCACACAGCAACCAATTACTATTTA

TTTAGTTTGGCCATGTCCGATCTCATACTACTGGTATCTGGGTTACCGCAAGAAATGTGG

TCTATTTGGTCTAGATATCCGTATGTATTTGGTGAAATATTTTGTCAACTTAGAGGTCTG

TTTTCCGAAATGTCTGCCAATGCTACTGTACTTACCATAACAGCGTTCACGGCTGAGCGG

TATGTAGCTATTTGCCACCCATTCATGGCGCAAAGCATGTCAAAACTTTCCAGAGCCGTA

AAACTTATCATCATCATATGGCTGGTCGCTGTGTTGTTTGCTATACCTCAGGCATTGCAA

TTCACAGTATCATCGTGGGACGGTTCGTCAGAACTGATGCAGTGCAATATTCGAAGTATA

CTGCTAATGGACACGGATATAGAACTATCTACCGTATCGTTCACGCTGTCAACGATGTTG

TTTTTCTTGCTGCCAATGACACTGATAACTGTACTTTACGCGCTTATCGGACTCCGCCTC

CGGCGCTCCGACAAACTGAAACGCACCATCACGGTACGATCAACACACGGCGAGAAAAAA

ATGTCATCACCCGAGTACAGGGCCAACTCATCACCAAAAGTCCTTAAAATGCTCGTTGCT

GTCGTCGTGGCGTTCTTCATCTGCTGGGCTCCGTTTCATGCGCAAAGGCTAATCGCCATA

TTCGGAATTAGTCACGCATCGTCTGGTAACTTGGACGCAAAGGACATACCGTTTTTACAT

CAGCTGTACGGTATTTCCACGTATATCTCTGGCGTTCTCTATTATGTATCCACCACCATC

AATCCAATACTATATCACATCATGTCTCTGAAATTCAGAGGGGCATTTAAGTGTTTTTTG

TTGTGTACCGACAGGAAACGGTGTTACGGTGTTGTGGCGGCGACAGCGGTTGCGGCGGCG

ACGGATGGCCCGGAAAATCCAAACAACAACGTCCGGCACGTTGGGCGCGGAGGAGGCTGC

GCAGCGACTCGTCCGAACCTACCACCGATGACCGCCGACCAAAGTACAGACGAGTATCCG

GACGAACGACATCGTCCATCGAATTACCGGCGACCGCCGACGATGACGACGACGACGAAA

TTTGCAGTGGCCCAGGTCCCGGCCCCAACGCCGAGCTCGCCAATTGTTGGCGGCGGGCGC*

>Ac_A36 c27060_g1

TTCAAATTTTCTTTGCTAATACTGCTTCTGTTAATTCAATTTGACATTGGCGAATCGCAA

ACTGGTATAAAAAATAATTCGACAACAGTACCTACTTTATGTAATCAGTGCTTGGAGTCA

GATTCGTGTCAATTAAATTCCACTTCAGACATTTATCATTGTGATGAAACATACTGTATA

CCAAAATCGTTTGTTTGTAATGGTATACCCGATTGTTTTCGAGGCCAAGATGAAGCTGTA

TCTGAATGTGGTTGTTTACAAAATGAGTACCGATGTAAGAACAAATGTATTGAACTGGTT

AAAAGATGTGATAAAATTGCTGATTGTGATGAGGGAGAAGACGAAATCGATTGTAAAACA

CATGTTTGTCCAACTACACACTTTAAATGCGCAAATTATTTTTGTATACCCACTGATAAA

ACATGTGATTTCAAAGATGATTGTGGTGATGGATCAGATGAACTTCAATGCAAGCACAGA

GAGTGCTGGCATGGAGAATTTAAGTGTAAAAATTCAGAATGTATTCGTCCTGGATATCTA

TGTGATGGTGAAGTTAATTGTGTAGATGGTTCTGACGAAGAAAACTGTGAAATAAGCGAT

TTTATTAAGTGCGGTGGGAGTCATTCAGTACACTCAACGTTTTGGTGTGATGGATGGCCA

GAGTGTATTGATAATCATGCTGATGAATTATTATGTAACATTTCGTGTTCGGAAAATAAG

TTTCAGTGCCCAAATGGAAGATGTATAAACGATGCTAATGTATGTGATGGACAATGCGAC

TGTTTACAAAGTATCGATGGAGACTGTGCCGACGAAATGAATTGCACAGCGTTTTATAAT

AAAACCGATGATGTGGTTGTGTGCACAACAGGATCAACGCTAAGTTGCTGGATGCCTGAG

GGAAATCCTTCGCGTTGCATACGCCAAAAATATATTTGTGATGGTCAAAATGACTGCTTT

AATGGATTTTCCATTTCAGATGAATTTGGCTGCGATAAGCCAAATTCACATTTAAATGAT

GAATTTTTCAAGTGTAAGGATGGCAGATGGTTGCCGTTTAAACACCGTTGTAACTTTAAA

GCAGAATGTTTAGATGGTGACGATGAAACTGATTGCGAAGTTTCGTTACCGTGTGATGAA

GATCAGTTCCGGTGTGCTAGTGGCGAATGTGTGAAATCCGAGAACCGCTGCGACGGCCGC

ACCGATTGTTGGGATAAGAGTGACGAAATCGGATGCATGACCGTTCCATGTCCCAGTAAA

CATTGGCGGAGGTGCGAGATCGGTAAACAGTGTGTGCCAATAGAAAAATGGTGTGATTAT

GCAGTTGACTGTATGGATGGTTCTGACGAGAAAAACTGCCAATATAGGTTATGCAAAAAT

GATGAGTTTCAATGTGATAGTGGTCAATGTATTCCGTTAGAGTATAAATGTAAAAAATAT

CAAGAAGAACAAATGGGTTGCGTTGACAAATCACATCTAAGAAATTGCGATGATTCTAAA

TGCGCTGAAAATGAATTCAAATGTCACAGAGGGCCTTGTATACATCAGTCTATGGTATGC

GATGGGAAATTGGACTGTGATTTGACTTGGGACGACGAAGACAATAATTGTTATTTCATG

TGTTCGGATATAGCATCTGGATGCCAGTGCCAGGATGTCCATATCAATTGTACTGGTCAC

GGGCTTGATCAATTTCCGTACGACGTCGAAAAAGAAATCACCTTTTTCCATTTGGGTGGT

AATAATTTTTCTGAGAGCCTTCATGAAAATACTTTTCAACATTTGGATCGCTTGGTTCAT

TTAGACTTGACGAATAACTCAATAAAACATTTGGAGCCATTTCTGTTTTCCACGCTTTGG

CGTTTAAAAACATTAAACTTACAAAATAACAAAATAACGATTTTGCAAAATTGTTCGTTT

ATGGGACTCGGTCAGCTAACGGGATTACATTTACAAGGGAACAATATTTACAAGTTGAGT

TCAATGGCATTTCGGGGGTTGTCTTCTCTGATTACACTTGACTTAAGCAATCAAAACATT

ACGGATATCGAATCTGAAGCTTTTGTTGGTCTTAGATCGTTAAAAAGTTTAGATCTATCA

TATAACTCTCTCACTTATATACGAGATGGCACGTTTCGTGGGATGCCCCAAGTTGTATTT

CTAAACCTTAAGAATAATCAATTGAGAGTCATAGACAAGAACGTTTTTTTTACTATGCCG

TTACTCGAAACACTGTTTACCGACGAGTTTCGTTTCTGCTGCTTGGCTCGATATGTGAAA

CATTGCGAACCGCAGCCTGATGAATTTAGCTCATGCGAAGACTTGATGTCCAACATCGTG

TTACGGGTGTGTATTTGGATACTGGCTGTGGTTGCGATTACCGCTAATTTGTTAGTAATT

GTGTTCCGGGCCAAATACAAGCATACCAACCAAGTACACTCGTTCCTCATCGTAAACTTG

GCGTTAGGCGACTTCTTGATGGGTTCGTATCTGCTGGTGATAGCTGTGGTCGACTGGTAC

TACCGTGGCGTGTACTTTATCCATGACTCTGACTGGCGACAAAGTTCCATGTGCAACGTG

GCTGGTTTTATAAGCACATTTTCTAGTGAATTGTCTGTGTTCACGTTGACCGTGATCACT

CTGGAAAGGTTATTGGTAATCATATTTCCGTTCAAAGTCCGACGCTTGGAAATGGATTTT

ACGCGTATATTGATGGCCGTATGTTGGTTACTAGCAATAATTATATCTGCCATTCCACTA

TTCAATATTCATTATTTCAGAAATTTTTATGGACGGTCTGGCGTGTGTTTAGCATTGCAC

ATTACTCCAGACAAACCCAACGGTTGGGAGTATTCTGTATTCATATTTCTATTTGTAAAT

CTGGGTTCGCTGTTGTTGATATCCGGAAGCTACCTTTGGATGTTTTTCGTTGCTAAGATG

ACTCATCGTGCCACAGAAACCCTAATCCGACACCGATCCCTCAGTGAGTCTGCAATGGCC

TGGCGGATGAGTCTGTTAGTGGCTACGGACGCTGCGTGCTGGGTGCCCATCATAGGATTA

GGATTGTGGTCTTTGGCTGGATTCACCGTGCCCCCTCAAGTGTTTGCTTGGGTAGCTGTT

TTCGTGTTGCCTTTGAACGCGGCGGTAAACCCTGTGCTTTACACACTCTCTGCGGTTCCG

ATAATCCGTCGTAGTGTAGTGTCAAACAGAAGAGCGGTGTCGCTCAGACGGTCTGTGACT

AACGACCCACATCCGCACAAT

>Ac_A37 c26277_g1

GAAACATCAATCCAAAATCTTCCAACAGCCGGTCTCGAAGAACTGGACGTTTTGAAAATT

GAAAAAACGTACACTATGAACGTTTTTCCGTCCATCTACAACTTCAAGAACATAAAAGAA

GCATGGCTGACATATCCTTATCACTGTTGTGCTTTCCATTTTCCTAAAACACACAACCCT

CAAGGATACGCAAACCACGAGAAACTCCAACAAAAAATGATGCACGAATGCAAAAATATA

CCATTTGTTGACCCTATCTCTACGGTAAAAACAATGCACTCAACAACGGAGAATTTTATC

GGTGACGAAATGTTTCATTCAGGAGATGTGACAATCGGTACTAAAGTTGACGTGATTTGC

GGAAACGTTTCAAAAAACTATCTAGAAGTCAAGTGTTACCCGGAACCGGATGCGTTTAAC

CCGTGTGAGGATCTGATGGGAAACTGGACATTACGGGTGGCCGTGTGGATTGTGGCCGTG

GCCGCTTTGCTGGGAAACATGGCCGTGTTGTTCGTGTTGCTGAGCAGTCGGTTCCGATTG

ACTGTCCCCAAATTCCTCATGTGTAACTTGGCCATGGCTGACTTTTGCATGGGCCTCTAC

CTGTTACTGATCGCCGTTATGGACGCTCGGTCGATCGGACAGTACTTCAATCACGCCATA

TTCTGGCAGAGAGGTATTGGTTGCAAAGTCGCTGGTTTCCTAACCGTGTTTTCATGCATG

CTGTCCGTGTTTACGTTGATGATTATCACCGGTGAACGTTGGTATACTATCACTTACGCT

ATACACCTGAATCGTCGGCTCAAGCTCGGCGCTTCCGTCAACATAATGGCTGTCGGATGG

CTGTTCTCGCTGGTTATGGGTGCGTTGCCACTGATGGGCACCAGTGGTTACTCGAAGACC

AGCATTTGCTTGCCCATGGACAACTCGACGTTCGCCGACAAGGTTTACTTGTTCTCCCTG

CTGACGTTCAACGGCATGGCGTTCGTGTTGATATGCGCGTGCTACGCCAAAATGTACGCG

TCCATCAGGGGCGGCCGGGAAGCGGTGGCGTCGGTGGCCCGGTCCGACATGACGGTGGCC

AAGCGCATGGCCCTGCTGGTGTTCACCGACTTCGCGTGTTGGGCGCCCGTGGCGTTCTTC

GGGCTGACCGCGCTCGCCGGTTACCCGCTCATCGACGTGCCCAAGACCAAGATCCTGCTG

GTGTTCTTCTACCCGCTGAACTCGTGCGCGAACCCGTACCTGTACGCGCTGCTCACGCAA

CAGTACCGCCGCGACCTGTTCGTGTTGCTCAGCCGGTTCGGCGTGTGCAGCCGTCGCGCG

GACGAGTACAAGGGGACGGGCCCGGTGGCGTGCGGCCGCCGCAAGGCCCACTGCAACGAC

GCGCAGCGGCACCGGGACCGCCGGGGCTACGGCGGCGGGGGCGGTGGA

>Ac_A38 c17409_g1 TTCAAAGAAATGAAAGACCTCTCTTCGCTAAATATGAATGGTATAAATGAAGAAAATATT

GATTTTTCATCATTTAATAATTTGACGACTAGACTGGAAGTTCTTTATTTAGATGAATTC

CATTATTGTGTTTTTCACGCTCAGCATGTTAAACTTTGTTTTCCAAACATAGATGAGTTA

TCATCGAGTTTGAATCTCCTCCCAAATTCAACTTTTCGATGGTTCCTATGGATGGTAACA

GTTCTAATATTGGTTTTTAACTCAATTGTTTTATACGGTCGGATGTTTAATGTATTTAAA

TATGACAATAAAGCACTTAACTTTGTCATACGGAATTTAGCTGTTGCAGATTTGTTTATG

GCGATATACTTAATAGTTATTTGTTATCACGATCAAATATACCGAAACGAGTATTATTTG

TATGCACATAAGTGGGAGTCATCTAATTTGTGTACCATGGTTGGAATTATGGCTGTGATT

TCGTCAGAAGTATCTATGCTCATATTGGTGTTTCTCAGTTTGGACAGATATATAATCATA

GGTTTACATTTTATTGGTAATCCAGGATTGAAAATGAAAACAGCCGTGTTCACTATGGCA

TCAATTTGGGTGGTCGGGATAAGTTTATCAGTTGCACCATTAATTTTATGGAAACACTCG

TCAAAATTTTACGGTTCTAACGGTTTGTGCTATCCCTTGTATATTGAAGATCCATTTGTT

AGTGGATGGCAATATTCGGCATTCATCTTTTTGGGATTATATGCGATAAGTTTATTGCTT

ATGACCGTTTTGTATGCATTACTGTTTAAAAATATCAAAGAAACAAGAAAACGTGCTAAA

CGAATTTCGACTGGTGATTTTGATTTGACAGTTCGTTTCTTTTTCATAGTTTTGGCGAAT

ATTTTATGTTGGTCACCAATAATCGTTTTGAAATTGGCAGCATTAAGAAAGTAC

>Ac_A39 c22388_g1

GATCTCGGTTATATAGATTATGCAAACGATAGCGAAGAAGAATTTTTTAACGAGCCAGCT

ATTACAAATAAAACCCAGGACATTATATGTGGAAATATTACTCCACCTAACATGGAATCC

GATGTCAAATGTTGGCCACAACCAAACGCCTTGAATCCATGTGAAGATATAATGGGTTTC

TATTGGCTTAGAGTGTGCGTTTGGCTGGTGGGAATCGCAGCGTTACTGGCAAACGTAGTT

GTACTGTTGGTGGTTTTCCGTAAAAAGTTTAATTACTCCGTTCCCAGATTTCTGATGAGT

AATTTGGCTTTTGCCGACTTTTGTACGGCCATTTATTTGTTGCTATTGGCTTACGAGGAT

TTGGTGTCCAGTGAAAAATATTTCAACTACGCTTACACATGGCAAAACGGCGTTGGATGC

AAAATCGGAGGTTTTTTGACGGTTTTTTCGTCTCAACTATCGGTTTTTGCGTTATGCTTG

TTAACGCTTGAACGGTGGTACTCAATACGCAGGGCACTATACACTAACAAAATGACGTTC

GCTTCAACTGTGAGAATAATGGCTTTTGGTTGGGTGTATTCAATTGTAATGGCAACCATG

CCGTTACTGGGCATTTCGAGTTATTCTACTACCAGCATCTGCCTGCCGATGGACACGGCT

CGCGCGAGCGGCATGTGCTACGTGTTCACTCTGTTGACGTTCGCCGCTGCCGCATTTCTG

CTGATGCTGTTCTGTTACGTGCAGATCTACATGTCGCTGAGTTACGAGACCCGTCACGCG

GCCAAGGGGGAGGCGTCCGTGGCCCGCAAGATTTCGGTGCTGATCGGCACGAACTTCGCG

TGCACCGCGCCCGTCATTTTCTTCGGCTTCACCGCGCTGCTCGGCTACCCGCTCATTGAC

GTCACCAAGTCGAAAATACTGCTCGTCTTCTTTTATCCCATCAATTCGTGCGCCAATCCG

TTCCTCTACACCATACTCACGGCCAGTTTCAGACGCGAAGCCTTCTCCACCGTCACCAAG

TTCGGTCTTTGTTTGGAGAATGAAAAAGAATATAAAGTGATTTATTCAACGCAAACCAAC

AACACCCAACGCTTAACTCCGGTACACAAACACACACTGTCGTTGACGTTGCCTCAGCCC

>Ac_A40 c25922_g1 ATGATATCCGCGCTGCTAACGGCTTTGTCTTTAATGATATTGTCGCTTGCATCAGCGGAC

GTAAATTTTATGCAATCTTGTGAGTCTCCTGCGCAGTGTCGATGCGATGGTAACGAAGCA

ACAGAAGTGTCCTGTAGAAATTTGAGATTCACCCGAATACCCGATGATATACCCGTCAAC

ATTACTAAGCTAGATGTGGCCAGTAACAATATTACGGAAATAGATGAACATGTATTTAGC

CGACTGACCTTATTGGAAGATCTGGTATTAGCTGATAATCCTATCAAAGAGATACACCCC

GGAGCATTTCTAAACAATATACGGTTAAAACGTCTGTCGTTCCAGAAATGTCAACTTGTC

CGGGCGCCATGTGAAACATTCCAGAGTTTCAGACAACTGTCGTCCTTACAACTAGATCAA

AACCATTTGACTGAGATCGACGATCGGTGTTTCGACCAACTATCTCAATTAAGGAATCTC

CGACTGGAAAATAACAAACTTACTAAAGTTCCTAAACAAGCGTTAAGCCTAGTCCCTACC

TTAGAAGCATTAAACTTGGGAAGTAATTCAATTGTAGATATTTCAAATGACTCATTTTCC

TCGTTACCTAACCTGGTCATATTGCTACTTAAGAGAAACCAAATCGAATTCGTGGATGAA

ACTGCGTTTGAGAGCCTTACTTCTTTGAAAATATTGGAACTCGACGACAATCAGTTGGAT

ACGATCCCGGTCGCTCTTGCCAAGTTGACATCTCTTCAGGAGCTGTCATTGTCTGGTAAT

AATATTAAGTTCGTGCCAGAAGGCGTGTTGCAACGATCACAAGGTCTAGCGTTGCTCGAG

CTTAAAGGCAACCCTTTGATTGGCGTGCACCCATATGCTTTCGCTTCCTTACCGAAATTA

AGAAAACTAGTATTGTCAGAGGCCAGAGAACTCACAGAATTTCCGAATCTTAACGGCACG

TCAGCGCTCGAAGTGCTCCGAATCGACAGAGCGAGTATTTATTCGATACCTGATACGTTG

TGTACGACGTGTCCTAAACTAAAAAGCCTTGACATCAAATGGAACAGATTATCTAGGATA

CCCAATCTCAATAAGTGTAAAGAACTCAGAGTGTTAGATTTGGCGAATAACCATATAGCG

TCATTGGAAGGCTCATTGTTTCGTAATCTGTCTCATCTCCACGACCTATTACTTGGTCAC

AATTATATAACTTCGATACCCAGGGACGCCTTCCAAGGATTAGTTCAGCTAAAAGTCTTG

GATCTAGAATCGAATAAAATTGACCGTATTGATGACGAAACGTTTTTGTCATTTAGCCAA

CTGGAAGATTTAAACGTGGGAAAAAATGTATTTGAACATCTTCCTACCAAAGGTCTGGAG

AGATTGCTACACTTAAAGACGTTTAATAACCCGAACCTAAGACAATTTCCAACTCCTGAA

CATTTTCCCAGAATTCAATCATTGGTGCTATCATACGCATACCATTGTTGTTCGTTTTTG

CCACTCCAGTCAGAAGATGATTCACCGCCTTCTTCTCAGACAAGTCTTCACGAATCTGTC

ATATTTCCAGCCAACGAAAACGATTTTGACATGACTCTCTGGAACTCTAGCATGTCTGAC

ATATGGCCTCAATTACAAAATTTAAGCAAAAAATTTGGAACGCAAGTCAATGATTTATGG

GATTCATTTGGTTCAGACTTCACGTATCCAGGAAATTTACCATCGTACATGGAGGAATAT

TTTGAAGAACAACAATTGGAAGGAAGGTCACAAGATGTAATGTCGTCAAAAATCAAGTGC

TTGCCACTTCCTGGTCCATTTCTACCATGCCAAGATCTCTTTGATTGGTGGACATTGAGA

TGTGGAGTATGGGTTGTGTTCTTATTAGCCATGTTGGGAAATGGGACTGTGGTATTCGTG

CTTATATTTGCCAGAAGTAAAATAGACGTGCCCAGGTTTCTCGTTTGTAATTTGGCCGCA

GCCGATTTCTTTATGGGTCTTTATTTAGGTATATTAGCTGTTGTTGACGCTGGCACTTTG

GGAGAGTTTAAAGTGTTCGCTATACCTTGGCAAATGTCTGCCGGTTGTCAACTGGCTGGC

GTTTTGGCAGTGCTGAGTTCAGAACTTTCGGTGTACACGCTGGCCGTTATCACACTGGAA

AGAAATTACGCAATCACGCACGCCATGCACCTAAACAAACGTCTGTCACTTAAGCATGCA

TCATACATAATGCTCTGTGGTTGGATTTTCGCTGGTGGGATGGCCGCGTTGCCACTGTTC

GGCGTGTCTGATTACCGAAAGTATGCAACATGCTTGCCATTCGAAACTACTACGGGCACA

TGGAGTTTGGTGTACGTTGTCTTTTTGATGTTCATAAATGGTGTGGCTTTTTTCATACTC

ATGGGATGCTACTTGAAAATGTACTGCGCCATCAGAGGCTCTCAAGCCTGGAATTCTAAC

GATTCACGAATCGCTAAACGCATGGCATTGCTCGTTTTTACTGATCTGCTATGCTGGGCC

CCTATAGCATTCGTCTCTCTAACAGCTATTTGTGGGTTTCACCTTGTCTCGTTGGAACAA

GCTAAAGTGTTCACGGTATTTGTGTTGCCTTTGAACTCGTGCTGCAATCCCTTCCTGTAT

GCCATACTGACAAAACAGTTCAAGAAAGATTGCGTGATGATATGCAAAGCTATCGAAGAG

TCGAGAGTGACCAGAGGCATCGGAAGGTGTAGGCACAGTTCTAACTTCAGCAACAGGCAA

ACGCCAGCCAACACCAACAGTTTAGTTGATAGGTCTTCCCGAGACAATTATCCTCATCCT

CCATGTAGCTGTAACGTTAAATTGTTGGATGACAAGTCTGTGAAAACGGAAGAGACATTT

TACGATTGGATCAACATCAAATTTAGAAGGATTATGTCGTGTTTGCAGCGAGAATCTGAC

TCAGGCAGACAGTACATGAGAAGCGACCGGTATGCCTACCAAATCGCTGAAATACAACAA

AAGCAGCATAAACGTGCATCATCGATGTCTTCTAGTGAAAATTACAGTTCGTCGAGATCG

GATTCATGGAGACAACACCACCACCACCATCATCATCATCATCATTGTGGTGTTCCCTTG

AGACTTCTTGATCCAAAACAGAGGCGTGCCAGTTCGTGGATTGTGACGAGAAAGACTTCA

CAAGACTCAAATCTGTCGAGCTCGAGAAATGACAGTTCGGGTTCTGCTAACACGGCCAGC

ACTAGTGTAAGCACAAGGACTTCCAGGTCTAGCGTAGGTTGTAGTGATCATAAACCAAAA

CCTCGGCTGACTCGCCAACCAGCTGTGTTAGACGACCCCGAGCTGGTCGTGGGGGGTTCT

CCTAATAGATTGAGTGTTCGATTTTTGGCAACCATTCCTTCAGCTGCTGAGACAAGTATT

TACAGAGCAGACACTGAGGACGAAGATGATGAAAACAGAATGGATGATTCGTTAAGGCCA

GAAAATTCACCAACTACTACTGCCAGTGAAAGGACTACACCACCGGATTCTAAACCATCG

TCCCCACATAGC*

>Ac_B1 c24053_g1

ATGACTACAGAAAGTCCAACGGGGATTTCCGCAGAATTTCAAAATCATTTGAGATCAGAA

TGTGAACATAAATTAAATGAAACAGTTTTCACACATCCAAGTTCAAATTTCTGTCGGGGA

ACATTTGATGGATGGCTTTGCTGGCCAGATACTGAAGCCGGAGACACCGTTCACCAACCA

TGTCCAGAATTCATTTCTGGATTTGATCCAACTAGAACTGCTTATAAGATTTGCAACAAA

AATGCTACATGGTTCAAGCATCCAATATCTGGTGCAGTCTGGTCCAACTACACAACTTGT

ATTAACCACGAAGATTACAATTGGACGCAACAAATCAATAATATTTATCAAACCGGGTAC

TTGGTTTCGTTCATTGCACTTTTACTGTCGATTGCCATTCTTACATATTTCAAGTCTCTG

AGATGTGCACGCAACACATTGCACACGCACATGTTCACATCGTTCGCTATCAATAACCTT

CTATGGCTGCTGTGGTACAGACTGGTCGTCGAGCATCCGTCAGTCGTACTTCACAACGGG

TGGTGGTGTCAAATATTACACGTTGTTCTACACTATTTTTTGCTAACCAACTACGCATGG

ATGTTGGCTGAAGGTTTTTATTTACACACACTTCTTGTGTTCGCCTTTACGTCCGAAGAT

ACTCTAGTTCGATGGTCTTGGACTTTGGCTTGGTCCACGCCATTGGTCGTCATTTCTTTG

TACACTGTACTACGAATTTCCAACCATCACACGTCCGAATGTTGGATCAACGAGTCACCA

TTTACAGAGGTGCTAGTGGTGCCTGTATGCATGTCGATGGCGCTCAACCTGGTGTTTCTG

TGTAACATCGTCCGAGTTCTTTGGGTGAAGCTTCAGGCCGGTCCATCTCATTTGTCCAAC

AGCACGCCATCACGAACACTTTTGCAGGCATTCAGAGCTACACTTTTGCTGTTGCCACTG

CTCGGGCTCCATTATCTGCTCACACCTTTCCGGCCACCCAAACAACATCCCTGGGAACCA

TTTTACGAGGTAGTGTCCGCTACTACGTCATCATTCCAAGGACTTTGTGTGGCAACATTA

TTTTGCTTTCTAAATGGAGAGGTTGTAGCCCAAATCAAGAGACGGTGGCAATTCATGTTT

TTCCGAACAAGGGCAAACTCGTACACAGCAACAACGGTCTCGTTCGTGAGATCGACCGCA

GGTCCAGGCGACAATGAAGATAAAGTT*

>Ac_B2 c17700_g1

ATGAATAGGATGACAACTAATATTCTAACTTCATGTGATTCTAATTCATCGTATAATTTA

ATTACTTCCAATGATAAATTCTGTAATTGGTTCTATGATGAGAAAACTTGTTGGCCCATT

GCGAAAGCCAATACCAATGTATCCCAACCATGTCCAAAAGAACAAGGCTTTGACAAAATG

AAGATGGCTTACAAATATTGTACAGAAGACGGAAATTGGGAAAATGTAACAAATTTGTTT

GGATCAACTGATTATAGTAGCTGTTATACTCCAGAACTAACAGAAATGCTAGAAAAACTT

GGATCAGACTATGATACAAAACGAAAACTCAATATTGCTATTAACACTGGAGTAATTGAA

GTAGTTGGATGTCTAGTATCTATCATTTCATTGATCATATCATTATGTATTTTCATCAGG

CATAAGGGTTTATTAGAACAGAGGTTCAAGATACACATGAATTTATTTGTTGCTTTGTCACTACAA

ATGTTCATCAAACTAATTTTAAATATTGATAAACTGGATTTATTCAAAACAAAAAAATAT

CATTCGAATGTTACTTGTCTAGTGGACTGTAACAACCAGATTCGTTTAGTGAAAGATGAC

GACAATTGGTTTTTTGGAATTATGTATACACCAATAATTTGTGAGGGTAGTCAAGTCATA

CTGGAATGGTCAAAAACTGCTAGTTTTATGTGGATGTACAACGAAGGAGTATATCTATAT

AGAATTATAAAATCTAGAGTCTTAAGAATAAATTCAATAAACTTTCGTATGTATCTATTT

GGTTGGGGTTTACCAGCTTTGATGACGTCTGTATGGTTATTGGTAACAATGGTTTATTAC

AAAAAATCGTCTAGTGTCTGTTGGTGGGGTTACAGTTTATTACCCTTTTTTTGGATCCTC

GAAGGACCCCGTATGATAATTATCGTAGTAAATTTCATAATATTAATCATTGTATTGAGA

GAACTATTGAAGAAAATTAAATCAAAATTATCACCGTCCAATGAGTTGGATATCATAAGA

AAAGTATCCAAAGCAGCCATAGCTCTTGTCCCTTTACTTGGGATCACAAACTTTATATTA

GTTATATTACCTGAACCACATTCGAAAAGTCCTGAATTATTTGCTTTTTGGTCATACTCC

GCACATATATTACACTCATTCCAGGGCTTAATGGTGTCGATATTATATTGCTTTATGAAT

AAAGAAATTAAAAAGTCGTTTGATAAACGAATGATGCTCCAAAAATCACGACGAAATTTC

GAAATGGAAATGACA

>Ac_B3 c25414_g1

ATGGCTGATTATCAATATGGATTGTCTAGTATTCAG

GATAATCATATTCTGCACCAAGTTATTGCTGAACGAAGACAAATTTGTGAATCCATCATA

AATGATCCACCAGTATCCGACAGTAATGACTGGTGTCCAGGAGAATTTGATGGCTGGACG

TGTATTAATCGAACAAAGGCTGGCGAAGTAGCCAAATTTCCATGCCCTTATTTTATTTTG

GGGTTCGATACAAAACGTTTTGGTCAGAAAACGTGCCTGCCGGACGGCTCGTGGTTCAAG

CATCCGGACAGCAACAAGACGTGGTCCAACTATACTACGTGCGTCGATCTGGAAGATCTA

AAGTTGAGGAATCAAGTCAACATGATCTACAAATGGGGATACACGGTATCGCTGGCTGCG

TTGGCGGTTTCTATATTCATATTCTTTTATTTCAGGAGCCTCACGTGTACTAGGATACAG

ATACATAAGAATCTGTTCATATCGCTGGCAGTCAATAATTGTTTGTGGCTAGTGTGGTAC

GAGGCGGTGGTCGACAATTTGCCGGTACTGATGACAAACGGGCTGGGCTGCAAAGTGCTG

CACGTTCTGGTCCAATACTTTCTGGTGGCCACATACTTTTGGATGTTTTGCGAGGGTCTA

TACTTGCACACGCTACTGGTCGTCACGTTCCTAACCGAGTCCAGAGTGATGCCGTTCTTG

CACACGATCGGTTGGGGCATACCGGCCCTGTTGGTGTCTACGTACGCAGTACTCCGGACA

GCCACTCCTGGCGAGACTTTACATTGTTGGATACACGAATCTCTTTACTCGTGGATATTG

TCGGGTCCTGTGTGCCTATCCATGCTGGCCAACCTCGTTTTTCTGATCAACATCGTCAGA

CTACTGCTAGTCAAGTTGCACGCCCGACAGATCACTATGTCCAGTCCGAAGCACGCGGTA

CCCAGGGAACGGGCGCAGTCGTTCAGCCTCAGGAGCTTCAAACGGAACAATTCCAGTGTG

GACAGTTACGATAAGGCACCGGCGACGTCGAGTAGGACCGGCAAGGCAGTCCGGGCAACA

CTCATCCTGATACCGTTACTTGGGCTCCAGTACATCGTGACGCCATTTCGACCAGAACCC

GGCACTTCTTGGGAGCCTGTATACCAGGTTACATCCGCTGTAGTCGCTTCCTGTCAAGGC

TTGTGCGTGGCGCTGTTATTTTGCTTTTTCAACGGAGAGGTACTTTCCGAAATGAAGAAA

CGGTGGAAACACTGCTGGATTAACAAGAACAGGTCGTGGAATCCTTGTCTGAGAGTCACA

TCCGTCTCCTCACCGCAAAACCATCCACGACTATTGGTGACCGAAGACCAACAGACCCAG

AGAACTGGAAGCATACAAGTTTTGCCGCGAAAAGATTCTGCTGAATGTTTACAAAATGTT

CAACTC*

>Ac_B4 c24744_g1

ATGTCGGACCCCGCCGGAGCCAGGGTGCACGATTCAAACACGTTGTTTCTGATTGAACTT

CGACAAAAATGCTTCAAAAACGCAAAACCAGTAAATGTGACACAGAAATTCTGTCCAAGG

TATTTCGATGGTTATGCATGCTGGGAAGAAACATTACCTAATGTTACTGCTTTTGCCCCA

TGTCCGAATTACGTGGTGGGATTTGATCCATACAGAACGGCGTTTAAGAGCTGTCTGTCA

GATGGCACATGGTACCGACATCCGGATACCGGAAAACAGTGGGCCAATTACACCAGCTGC

GTGGACGTTGACGACTATGAGTTTCGGGATCTAATAAACAAACTGTACATCTTTGGCTAC

TCAGTCTCGCTGGCCGCACTGCTCACGTCGTTGCTGATTTTCTTCACGTTCAAGACTCTG

AGGTGCACTAGGATCGCTATACATGTGCATTTATTCATATCGTTGGCATTGAATAATCTC

ACATGGATCATTTGGTACAAAACGGTCGTGCAAGACCTGTCGGTTGTTCAACAAAACGGG

ATATATTGCCGGTCTCTGCATGTGGCTCTGCAATTTTTCATGGTGGCCAATTACATGTGG

ATGTTCTGCGAAGGACTGCACTTGCACATGGCCTTGGTTGTGGTGTTCGTCAACGACGTG

TATGCGATGCGGTGGTTCTACGCTATCGGCTGGGGCGCACCCGCCGTACTTACCATCCTG

TATGTTTCCTGGAGGAGTAATTCTGAAGACACCACACAGTGTTGGATGCACGAGAGCCAC

TGCCAGTGGGTGTTGACAGTACCGGTTTTCGCCTCCATACTGACAAGCTTACTGTTCCTG

ATGAACGTCATGCGAGTGCTGCTGACCAAACTGCACCGAAACTCGACAAACCCGGCTCCA

ATCGGCGTCCGCAAGGCGGTCAGGGCAGCTTTGATCCTGGTGCCCTTGTTTGGCATACAC

TACATTCTCATACCGTATCGTCCCAAGCACAAGACCACCGTGGAAATAATTTATCAAATA

TTTTCCGTAATACTTGTATCGACCCAGGGCCTTTGCGTGTCAGTGTTGTTCTGTTTTGCA

AACGTTGACGTACACGGAGCGTTTCACAAGTATATACGGAGGATCAAGAGACGCGGCACC

ACGCACACCAACGTCACAGGCACCACACAACCAGTCCAGTCGCAAATAAGGGACGCCATC

GTC*

>Ac_B5 c27642_g1

ATGAATATCACTACTACAAACAACACTTGTCCACCTGAAAAAGTACTAAATCCTGGTTGG

TGTCCTAATCACTGGGATCTATTGTTATGTTGGAAAGCTTCGAAACCTGGTGCTGTGGTT

TACCAAGCATGTTTTGATGAATTGAACGGAGTGCGTTATGATACATCACGAAATGCTAGT

CGTCGATGTAAATCGAATGGTGTTTGGGAAAATTCTTCCGATTATATGAATTGTAAACCT

TTGGATACACACAGTTCATTGTATCCAGATCCATCAATTGTATACACATCATACTTTTAC

TATGGTGGTTATACCATATCATTAGTGGCATTGGTGGCAGCTGTTTCCATATTTGTTTAC

TTCAAAGATTTACGGTGCTTGAGAAACACAATACACACAAATTTGATGTGTACTTATATA

CTATCTGATTTTACATGGATATTGACGTCAACATTACAGGAATGGCTTTCAGCTAGTAAT

AATGCTTGTGTATTATTTACATTTTCATTACATTATTTTGTTTTGACAAATTTCTTTTGG

ATGTTTGTCGAAGGCTTATACCTGTATATTTTGGTTGTTGAAACTTTTACCCGGGAAAAT

ATCAAGCTCCGCGTTTACATGTCTATTGGCTGGGGTATTCCACTTGTAATTATGATTATT

TGGGGTGTTTCTAAGATCATCACACCAATGGAAATAGAGGAAAGAAGCGATTCTTGTTCA

TGGATGACACCACACCCCGTGCACGATTGGATCTATCAAGGGCCGGCAATAATAGTGCTG

ATAGTAAATTTAGTTTTTCTATCAAAAATAATGTGGGTACTAATAACAAAGTTAAGGTCA

GCGAATTCGGCTGAAACGCAACAATACCGCAAAGCCAGTAAAGCTTTGTTAGTTCTTATA

CCATTGTTAGGTGTTACCTATATACTAACGATGGTCGGGCCTACGGAATCCGGTACTTAC

GCTAACTATTATTCTTATGGCAGAGCTACACTTTTGTCTATGCAAGGCTTTATGGTGGCA

ATATTTTACTGCTTTGTCAATTCCGAAGTAAAGAACACGTTCAAACATCATTTCGCTCGG

TGGAATGAGGCAAGAAATCTGAGAACCCGCGGTAGCCGAAGATTCACTTATTCAAAAGAT

TGGTCACCAAATACAAGGCCCAGTGTTCATATAGGCGAAAAGAAAACTACAGGATGCGCC*

>Ac_B6 Unigene0036280

ATGGATTATCTCAATGACAGTTGGTCACCAAACGAAGAAGCTGCTTTGAAATGCAGTTCT

TGGTACGAATGGGATGATCGTTGGACAAACGGGTGTAATGCATCATCAGATTCGCTGATA

TGTTGGCCTCCTACTCCTGCTGGTGTAATCGTATATCAGCCATGTTTTCAAGAGCTCAGA

GGAATATTATACGATACAACTAAAAATGCAAGTCGAATATGCTTCGAGAACAATACTTGG

GGCCTAACCGATTTTAATGAGTGCACAATTCTTGGTGAGCCAAAAGCGGTGACATACGAT

GAAGAAGACACTGTTGATACAATAATTTATTTGTATATAGCCGGTCATTGTTTATCCCTT

ATAATGACATCGCTGGCTATTTTTGTATTCTGCCGATTTAAAGAACTGAAATGTTTGCGT

AACAAAATCCACTCAAATTTGATGGCATCGTACTTACTAGCAGGTATCATGTGGATACTT

AACTACACTAACTTGACCGACACAGGGACATTTAAATGTGCTTTGTTGGTGTTACCACTT

TACTATTTCACCATGACGAATTATTTTTGGGCGTTTATCGAAGGGATGTATTTATTTATA

TTGGTCGTAGATACATTTTTTCCAGATAGAGTGCGATTGCGAACATATATGGCTATTGGA

TGGGGAATACCTTTGATCATTATTCCCACTTGGTGTGTTACAAAACTTTTGGTTCCAACT

AAAAACGACTTAGATTTGTACAACCAGATCACATACGAAAGATACTGTCCGTTAATGGCC

TCATACGTTGACGACTGGATATACCAGTCACCGATAGTCATCGTGTTGCTGATCAACTCT

GTGTTCCTTGTAAAAATTATGCGAGTGCTGATAACAAAAATACGGTCCACGAAATCCGCA

GAAACGCACAACTACAAAAAGGCCACTAAGGCTCTTCTAGTGTTGATACCACTGCTGGGC

ATTACTTTTTGTTTGGATATGATCAATCCCAGTTCTACCGGACTACTGGTTAATATTTAC

AAATTCAGCAAAGTTGTAATTATCAGCACGCAGGGATTTACCGTCTCGTTACTGTATTGT

TTCTTTAACAATGAAGTTCAAAGCACATTGAAATACCATATAACTAGATGGCAGACAAAG

CGGAAATTCCTTGCTTCGAGAAAAAAATATGGACGGTCCTGGTTATCAGTAAAACAAAAC

AATATAATATGTGATCATCAGTTGGACCCAAAGGAACTTATGCCATGGATACCAGCTAAC

AACGTTAGATCATCGTCTTGCGTCAGCAACGACACGACTACTTCAGCGTTGTCAAACATG

AGACTGCCCCCCGATACGACTATCGAAATACCACCGCATCCAGAACAACAACCACTATAC

GATGAGGACACAAAATATGTCGAAGACAACCGTGAACCA*

>Ac_A1 c25397_g1

MDYPIAMPLTTVWPTGAGTYNGTDMGPYGNGTGDRDALYCDDTSGHESSLYLISKILYVI

VCVIGLVGNTLVIYVVIRFSKMQTVTNMYIVNLAIADECFLIGIPFLIVTMSMEFWPFGN

VMCKVYMTTTSVNQFTSSIFLMIMSADRYIAICHPISSSKVRTAYVSKIVSVTAWTFSII

LMIPVIMYANTMDKGNVKSCNIIWPENELFSGQTAFTLYSFVLGFAIPLMLIFVFYILVI

RKLQTVGPVNKSKEKKKSHRKVTKLVLTVITVYVLCWLPYWITQVALIFTPPKQCQSKIV

ITIFLLAGCLSYSNSAMNPILYAFLSDNFKKSFLKACTCAAGKDVNAGLHQENSTFPRRN

RGGSERGGGRTGRATTILCQAVSGGTGGGGAGGCCKDDDGGCCGETGPLVGRAELVSKEN

TSTALTMTSRSTCNENNVQPAQL

>Ac_A2 c19014_g1

MNPTAGMPTALEGTAMYRYEMCWNISA

GNFPLCVNSTEFNDFNETDDEILQVQRIVSLAVPILFGIIVVVGLLGNLLVVIVVMANQQ

MRSTTNLLIINLALADLLFIVFCVPFTAVDYMLPYWPFGDVWCKMVQYLIVVTAYASVYT

LVLMSLDRFLAVVHPIASIYVRTERNASSAILVTWVLIVLLALPVLARHGEVKYTFSSIE

HTACIFLDRDQKTRPDGYNKPAYQIFFFLTSYAIPLAIICILYVLMLMRLWKGVNPGGQP

PSAESRRGKKRVTRMVVVVVAIFAFCWFPIQVILVLKSINYYEITAISVIVQIVSHCLAY

TNSCLNPILYAFLSENFRKAFRKVIIPWRPEVNVQGRFANGDARSMAVTRTTRTTNNDIL

>Ac_A3 c23632_g1

MLCTDLSSDRQSYNYTWIAELRKLNLTQEHLQYLLNITTSYDDDDLDVGCYNCGGAVKSY

SLLFREVHGYVSLFLCVFGALANALNIAVLTRKDLAGSPINRILCGLALADLALMVEYTP

FACYMYLSTAKKEEFSHVGAVYVLLHTYVSQVLHTTSIALTLVLAMWRYVVVKLPNSMHA

ICSDRRCTIAIKLSYLLPFIICSPTFLVFEILETRVVENGTVATLYHLGLSTIARVNHEL

LYMIHLWTYAVIIKLLPCLILTVVTISLINALSEASERKAKRLPTQQQMARIRNMKLKKR

MDRTSRIMIAVLLLFLATEFPQGILGLLSGILGRGFFRTCYNLFGELMDMLALLNASLNF

VFYCCMSKQFRVAFGQLFKTQPSIMFKPSNILETFV

>Ac_A4 c27545_g1

MVIEPGLMDMLGNSLFDMLAPVSSTESSAVGSTVSAHGSGDGSGGRVDGSGSAVDDDEDD

GSGMGGGSGAGGSGGIGPGELWYRHSPAMTAVYCFAYTMVFLVGLVGNLLVVSVVCRSPR

MRNVTNYFIVNLAVADILVLVFCLPATLLSNIYVPWILGSWMCKIVPYVQGVSVAASVYS

LIAVSVDRFLAIWYPLKCQITTRRARYIIAIIWLASTTITIPWALFFDMVAIFKDAPNLE

LCLEVWPDYLDGNLYFLLGNLGLCYVVPTVAISLCYVMIWVKVWRRTIPTDNKCARMERI

QQQSKVKVVKMLAMVVVLFVASWLPLYAIFARIKLGGRLVPWEEDFLPVATPIAQWLGAS

NSCINPVLYAFFNRKFRRGFTAVLQSRRCCGTLRYNENLQHSASASGNGGGKASSYYITN

HHAYTKRQSSQETNVSYIFNV

>Ac_A5 c24582_g1

MEFPSRDANQSGEAFWDYDGPDNWPWQKYRMRYSPEVTVLFCIAYTAVFVVGFV

GNMSVVLVVYKNVRMQSSPTNIFIVNLAIADLLVIVVCVPFTLIGSITTEWRLGLVICKL

VPYFQGVSVNASINTLMAISVERCLSICYPMNPVGKGVCKRVVAIIWIISLTITMPWAIY

FDLQPMEEGSDNQICLESWPTVESGNLYFVLANLVLCYVLPLTVIAVCYMFIWQKVSRRK

VPGEPVHNGANMVQRSKMKVITMIMYVVVLFAVSWLPLYVAFSLIKFWPLPPAVESYTVA

SLPVAQWLGAANSSINPLLYAIFNHRFRDGYRALLSGKICQAFDYSNSVRYLRGGGRAGT

AAAFKRNNNDYDNGGGRDRNRKTIGAIYVHATR

>Ac_A6 c25283_g1

MISALDVVAFGNDSSPLTVTGNDTAGGAGPSSNDTAANGVIQFLGDDLSFPDYIRTTCMVVCVIILGVGVVGNMMVPIVILKSKDMRNSTNIFLMNLSIADLMVLLICTPTVFVEVNSRPETWVLGEELCKAVPFVELTVAHASVLTILAISFERYYAICEPLRAGYVCTKTRAMIICLLAWGLAALFTSSQSDHMISEYTQMDYIDGTKVPVCLTKANTFWPIAFFVTIIGVFFVVPLFVLVVLYTVIAVHLMADPGTSCTDSACNQRARRQVVLMLATVVLSFFVCLLPFRVFTMWIILVPEHTFLDLGVKHYYIILYSSRVMVYLNSAVNPILYNLMSSKFRRGFCKLCRSQCGGGGSVDDCYDSYDGGGAGGCVIGCGG

>Ac_A7 Unigene0031155

MANGSGGGGFEALQNVA

PNVFPPIYISPGHGSNGTNAVNYSEAIEREYVEDYVFQSAFSFVYLLIFTLGVFGNVLVV

YVVWANKHMRTVTNIFIVNLAVSDIMLCGLAVPFTPLYTFTGHWMFGEIFCHIVPYAQGT

SVYTSTLTLTSIAIDRFFVIIYPFQPRMTIWTTAQIIATIWIFSLVATLPYGIYMANKEI

YGKDFCEETWPQETFRKIFGAITAILQFVLPFLIIAFCYIRVWLKLNDRARCKPGTSTKN

ARREEVERERKSRTNRMLIAMVTIFGVSWLPLTAINLLNDFYLQTATWKHYYLFFFSAHA

VAMSSTCYNPFLYAWLNENFRKEFKQVLPCWRNGSGYGTTGADLGQGRRGRVGGYRSERT

CNGNDTCQETLLPTSIVLPSGRTTATTDCTGLDLVDGLMMGDQEDNQDAVEVMLVAYTRD

DGISGRSGNVQQNSIKTQV

>Ac_A8 Unigene0019560

DNCAALIIQDQKYCYFRKAVHFWAQQALLPAVVAVGVVGNMLSVVVLTREPMKSSTSTYL

TALAVSDLFYLLFVFTISFENYPWIVEADYYIYWKWYPYGLWLTDAASNTSVLLTVSFTV

ERYIAVCHPLRGRMLCTESRAKRVILIVALFCIACTATTPYEWHIAINAATGKFQKSSTE

LGRNDIYKKAYNWFCIVTFICVPLLVLAVLNWFLINAVNQSRRNRTRLTCQGNMVWNRQR

QENKMTMTLIAVVIMFCVCQTPTAVMMLTASVYEPPEKTPAYYVNRGLHTIFNFLMVVNA

ASNFMLYCAMSRKYRRTLMITFMPFLAARHARNATLRSSVSYPRSGTIVRRNTEVTQMSD

ITGVSSSAAAAGKRELLVRSGTNHGRHAAAANNNNKQQQLTATAVL

>Ac_A9 c13254_g1

MSSSNQSVGFEPWLKENPKIMYAAAFMTLLIMCIGIIGNSLTILVILKSPRIRNVASTFI

ISLGAADLLFCIAVLPFNASRFLNINWVQYPELCSIVPFLQYGNIGVSLLFITMITINRY

IMIVHSSLYSIVYRPIWITSMIMLCLIISFGMLIPTLLSKWGKFEYDPKLGTCSIVSDEF

GQSSKSALLVTGFIIPCIVIVCCYTGIFLVVRNSEKRMRRHQTSTQSDPTATNMQSIRRK

LSEWRITKMVLAIFLSFVLCYLPITITKTLDPGVQYPALLLIAYIMIYASACFNPIIYVI

MNKQYRKAFKSVLNYGCCEKATKRVPVLRSISKRLQPHGNLTSSTVMQTLQTISPG

>Ac_A10 c21429_g1

MDEENYTAVVPTYLFDFNGTANGGNDTEGYLNVTT

EMPVRYARPMYGFVMPFLLLVTIVANTLIVVVLSKRHMRTPTNVVLMSMALSDMFTLLFP

APWLFYMYTLGNHYKPLSPVESCYAWYAMNEVIPTLFHTASIWLTLALAVQRYIYVCHAP

VARTWCTMPRVLKCVAWISVMASLHQSTRFVDRTYEPIKISWRGQDSVVVCRMKHAYWVE

HWVTLDVYFTLYYAFRVIFVHTGPCISLVVLNLLLFRAMRDAQLKRDKLFKENRKNECKR

LRDSNCTTLMLIVVVTVFLMTEIPLAVVTVLHIISSSIKEILDYSVANLLVLFTNFFIIV

SYPINFAIYCGMSRQFRETFKELFIRGSVQINRKHGAGGSSRYSLVNGPRTCTNESLL

>Ac_A11 c44582_g1

CCVIYMMVGVPGNLITIIALFRCKKVRNATAVFIINLSVSDLSFCCFNLPLAASTFWYRS

WIHGQLLCRLFPLVRYGLLAVSLFTILAITINRYIMIGHPSLYPKMYKKFYLGVMVTVTW

VGGFGLLIPTWLGKWGQFGLDVTVGSCSILPDSV

>Ac_A12 c18209_g1

MDQLQSANQFDDLPHKWVDVVADYLRGFRNDTTDFNRPQLRSSVRHTYPAFVCGYSALIM

AGALCNAYVLAIVARKRLYATDPVYVYVANLAVTGIVECVSVLPISLMVLLVQNWIFGRF

LCFFLPMLQDVPTHVIMLTFLLMAIDRYKHLKHPNKMRLPPLACTFGCWIVAFCIVLPYP

VYTAYLDLGAYIKVQFEGVGICAVNMADDMQDYLRSLFVLTYLVPLVSMGYLYSKMSEIL

RDLMNLPAVFYSQDLTPRSCEIPSRLNEVQSTVSDSDDEEIDGYKESKTQKYLISMIISY

AVCLCPLMILRLAKLEVSETYENSRHFDLTFMICVWLAFVPTVTTPLLFVAWNSDSSTKD

RIRSYFKRTKVTAEQATRTVSTEGLAARNSIYTVQESIPA

>Ac_A13 c26478_g1

MENGNGTSGADGGAAHISPYCGDMLVDLHDVYVHYHGYASLLVCAFGSVANVLNIAVLTR

KEMVSPTNAILTGLAVADLLVMVEYVPFAYHMYLRPTNYPRADRFSYNWSLFVLLHSDFS

QAFHTISIWLTVTLAVWRYVAVVHPQLNRIWCRMETTLSTIALGYVVCPIICIPSYLSFN

LFSRVETLDANGNRPTAVLQTALRRANNGTDVHNAHVIGGGSGGSGAVGGGSSAGAVNGG

SGPLRNVTLYYVNVSDLATSTYLADINFWVYSVVIKIIPCVALTVLSLRLICALLEAKRR

RAKLTGSGRKSADKERQTDRTTRMLLAVLMLFLITEFPQGILGLLTLLLGKRFFQDCYQN

MGEVMDMLALVNSAINFILYCVMSRQFRNTFSLLFLPSWISKVESQALSHGNPTTTQVTQ

V

>Ac_A14 c12924_g1

VIVSCVPFTSTVYTFPTWPYGLAVCKVSETAKDVSIGVSVFTLTALSADRYFAIVNPMRK

LHASIGGRLATRFTLTVAAAIWAVAVVCAVPAARFSYVRQFRVHNVTLFEACYPFSEHLG

PAYPKVMVTVKFLVYYAVPLAVIACFYVLIARYLLHTTNNMPGELQGQIRQVRARKKVAK

AVLAFVLMFAICYLPHHVFMLWFYNYPKSTDEYNTFWHVLRIVGFCLSFINSCINPIALY

LVSGTFRKHFDKQLFWWCMASSAVTTESNLFVIKKNGATSRDTKITEFIMLPSMHSTANK

NKSNKTFTMIANTE

>Ac_A15 c24117_g1

MAASIAISGNYTDVNDSD

NVYEPYSNRPETYIVPVVFAMIFVVGVLGNGTLVLVFIRHRSMRNVPNTYILSLALGDLL

VIITCVPFTSTVYTVESWPYGELICKLSEATKDVSIGVSVFTLTALSAERYCAIVNPIRR

HVSSKPFTLMTAVAIWILAVVLATPSATFSHLATESIPNTNVTIEYCYPFPIELGNGYAR

GMVMFKLLAYYVVPLCVIGCFYLLMAHHLMVSTRNMPGELQHAGQSGQIRARKKVAKMVL

SFVVIFMVSFLPYHVFMVWFHFNSNSRDEYDDYWHAFRIVGFCLSFINSCVNPVALYFIS

GVFRKHFNRYLFCCCPFARSGPATVESTIQDINLTHVNSTSCRRHNSVVTSHATTLGHAT

>Ac_A16 c27080_g1

MHSFTLGSNKMWVKWTLITMIAFSTTGVTEGHINMTTRRESTIPPTAIENDTEYDEMANYTDE

EYRNYLVEYVTPRKSEWVFIVMHSMVFVVGLIGNALVCVAVYRNRTMRTVTNYFIVNLAV

ADFLVILICLPPTVVWDVTETWFMGTIACKLVLYFQTVSVTVSVMTLTSISIDRWYAICH

PLKFKSTTSRARTAIIIIWIVGLASDIPELLVLEAIEKTKRVSSIYLTQCEALWSQESET

IYQIAKTIILYILPLLLMSVAYYQIVRVLWKSDNIPGHTETVQMFNANAYNGFNRTATMG

CTSTMAQIKARRKAAKMLVAVVVMFALCFFLVHLMNLLRFTVGIQQSQATALVSNLSHWL

CYANSAVNPLIYNFMSGKYRNEFKRLFLCWGSHRQNRLRRAAHTSRSGTYICRFTMTTMK

TDNVSFGLSPEDIQ

>Ac_A17 c24908_g1

MDDLVNNPEYFLKHWTNNLTRHISVPSAHEMFNDTTVR

NVSSLWTKNAAGMEQAAVIDELIAEFSSSQMKFTETRSIMLIGLYVPLFLVAAIANSVVI

VVVIKYHYMRSVTNYFLVNLSIADLLVTFICMPMAVGQSVTGLWLYGETMCKLTSYLQGV

SVGASVFTIAAMSIDRYLAIEHSMSFRKVLNRKSTIYVILALWLVSMTIFGPVLWVRQTE

SVELGDDPILIDAVHRYGLAWCIEDWGNAHAKSTLSKHVYGILCFVLVYATPGFLVTGAY

TLMGRRLWAVRPPFDDQQGMISVQQVRMVRERRRVARILFVLAVIFALCWLPYNLLTLFL

DLDITLDKFGLDQEYLMKWYPFTLLLGHANSAINPLLYCFMTRNFRRTIKGFVCNTGIAK

PRRRNRCKKGLTEKSTTSGYGSFRNPRRLCFTLAQLRQNNTVQTRTATISNLAVL

>Ac_A18 c25234_g1

MSTHTNEDLEDEISLTDRFVKYYPLAVVCLGSLGNCLSVLVFFGTKLRKQSSSYYLSSLA

ISDTLFLLIQLMPVLSKVGIGIYHMHGFCQFFVYLAQICSFISVWLVVVFTSERFIAVRY

PLHRSVICTVYRAKIVLFILITFALIVHIPYLVISTPNSTVLENNSTTTECSLNFSWYEL

YKWLNYADVMMNMLIPFFLIVIFNSMICQSVCRLARIRRTMTLHPSRRRQSTSQNSQHTS

QIKVTEMLLVVSTVFLCLNLPSYVFRVWMVWDNTSTKYKTIQVIANQMYNTHFGINFVLY

CVSGQNFRRALVELWNKRRHPNKRLRETQVTTVLSEFSKSGVGSKQTTVNGTWKEVHELI

PIAHS

>Ac_A19 Unigene0035849

MDGGSGVVNNTSNSTLLVDEYDYSERPETYIVPVLFAFIFFVGTVGNGSLVLIFIRHRNM

INVPNIYILSLALGDLLVLMSCIPFTSTVYTVPSWPFGLTICKVSETTKDISIGVTVFTL

TALSADRFFAIVDPMRKLHASVGGRRATKFTVTVAVTIWCLAVACAVPAATNSYVRQFQQ

NNVTLFEACYPYAEELGPTYPRLVVVIRFLVYYVVPLSAIACFYAMMARHLIHSTRNMPG

EVQGQMRQVRARKKVAKTVLAFVLVFAVCFLPYHVFMLWFYLNPRSQDEYNIFWHVLRIA

GFCLCYSNSCINPIALYLVSGTFRKHFDRQLFWWLVKPPGGQITESKNGYMRRKNGTREK

DRTINNDSTTIANVQLSTFTRRTADTNHTNTTTVLICTGLNDANAII

>Ac_A20 Unigene0006642

MILIGFQTTVYLMYTAVFVVSLVGNGLVCYVVLSSTQMQSATNMFIVNMAVGDLVMTLFC

VPFPFVTTFLLEYWPLENYVCQIFTFGKIVAVMVGAYTLVAISVNRYIAIMWPLKQRTRK

HQAKYIIALVWTMSVITSFPFLLATSLDQKFDIYFCTDKWSSEFIRRFFNAALFLLQCCI

PFAVLLFTNIHIGVVVWGKLPPGEAQNSRDIKMAKSIRKMIKMMATVVIAFIVCWLPYDI

LLVLRAYGMSLRAWSNQPYVWFAFHWLAISHTCYNPLIYFCMNTRYRAGFVSALRNVPGL

GYTRAQQHGDELPETGRPRVASVVSV

>Ac_A21 c21731_g2

MSESDDEPSATTWNVTATVAARWNGTEAVDELYCGADGSAAFQTLVYLTYTIVFVVSLVG

NGLVCYVVVFSAQMHSVTNLFIMNMAVGDLLMTLFCVPFSFVATLLLQYWPFGSDLCHTV

SFAQAVAVLVSAYTLVAISVDRYIAIMWPLKPRASRHQAKYIIALVWTVAVITAFPILLV

TTLEQPSSWHQECGLYICNEKWSSENVRHYYNVALLVLQYCIPFAVLLFTYVNIGVVVWG

KRTPGEAQNSRDVRMAKSKRKMIKMMVTVVIAFTVCWLPYNILLILWDHEPSLSTWSSLP

YVWFLFHWLAMSHTCYNPLIYCWMNTRYRTGFAAVLRNVPGFGRCLGGYLRATQNQSHRY

NSHNDPSQADGLHRINTTSSFVSVKSRLKSFNGRPAAYGRNRQNWHEERL

>Ac_A22 Unigene0038062

MAAAEIATKIAVNFTENLTANITANVTSNVTVNDTLLHDQTTSDLYKVPALLVVVLSVLY

GSISVIAVAGNGLVIWAIVTSKRMRSVTNHYLANLAFADILIALFAIPFEFQAALLQRWN

LPSFMCAFCPFIHVLSITVSVFTLTAIAVDRRQAILNPFAARTSKTQCLCVIALIWIAGL

VLSSPMAYAQRVVFVSEDWPFCLNVNLSNNVMLVYRALLVVVQYVIPLSIMTWAYSGIGF

ALWGSSAPGNAQSQRDLNLMRNKKRVIKMLIIVVALFTLCWLPLQTYNILQHIFPQINEY

PYINIIWFCFDWFAMSNSCYNPFIYSIYNEKFKQEFKMRLDFMAGKRRLTRDLSAFSSGR

FEWRTNHVNTHERNLKHSSIVTNDTPLILTPDA

>Ac_A23 c18353_g1

MDENNYKIVCNESLDENINIKSNNSIAIDRCNDYSNYTNHTDLYLETTFLYDVPVSIIVL

LSMFYGTISVMAVVGNALVIWIVTSSKRMHNVTNLYIANLALADIVIGLFSIPFQFQAAL

LQRWNLPAIMCPFCPFVQVLSVNVSIFTLTAIAVDRHRAILSPLSAIPSKFRAKMSIATI

WAIAFVLATPMAIALRVQFIEYGDRDGRKLVKPFCYNVRLPERSMLFYRITLLFVQYLVP

VVIITVVYMRMALRLWGSHAPGNAQDSRDANLMRNKKKVIKMLVIVVGLFVLCWLPLQTY

NVLQDIFPSINQFRYINIVFFCCDWLAMSNSCYNPFIYGIYNEKFKREFRIKLRHFRRGR

FRGKSNANHGRFASMRSTTLSEWKRGYSTRCTDRTTANGVCVDSPPPRRDELEMFVCKSG

KITLIKCGSRSDLEELCL

>Ac_A24 c21909_g1

MNGSDFGGPNLTDNGTDGVVWPDSNQFELPWWHQLAWTVLFVPMIMVATGGNLIVIWIVMT

NKRMRNVTNYFLVNLSIADAMVSTLNVSVNFSYMLTSNWTFGTAYCKISQFVAVLSICAS

VFTLMAISIDRYIAIIHPLRPRLGRKTTLMIATSIWVVGTILSIPNLIFFTTHTELFPNG

DQRVICYAEWPDGITTNSFQEYVYNVSFMIITYFIPIGLMGFTYAMIGHELWGSQSIGEC

TQRQLEHIKSKRRVVKMMIVVVTIFAICWLPYHIYFIVTSHMPELTTSPYIQDIYLAFYW

LAMSNSMHNPIVYCWMNSRFRQGFKQFFSFVPCINVRTGSLIRREVVTSRYSYSGSPDAH

YRIVRNGTVCIPLNHLCNDNGRGHLKVPAAHWRQAKKHGIDSSVGEMSSTSFSNAVTGVT

IAMASTSTCNENSINCNEDSLKTCLGKNANSAS

>Ac_A25 c19757_g1

MNNTTSGSYMVQLDDTANETVEGYLLRTRGPKHLSLNIVLPITIIYVFI

FVTGVIGNIAVCVVIVRNNFMHTATNYYLFSLAVSDLTLLLLGLPNDLSVYWQQYPWPLGEVLCKFRALVSEMTSYTSVLTIVAFSMERYLAICHPLHSYAMSG

LKRAVRIIAVVWMISFFAALPFAMFTTVDYVDYPPGSGDPLYESAFCAMLDKNVPTGVPV

YELSSLLFFLVPMMIIIVLYVLIGLQIRQSSRHSLGKQMQGNVHGETKQIQSKKSIVRML

AAVVIAFFLCWAPFHAQRLLYLYAKDSPYYFQANELLYTIAGCFYYFSSTVNPILYNLMS

MKYRRAFRETLCGYSGDRRNRMSRELQSSFRDTTVPLNTTISTADCSRKSVVNRSTRNLQ

QSDAPYNHHHYAAAPSSDDCSSGRPPATASDVLVMISPVNGNTQCYKTLLRVTVQGPDNKTTTTTEHGNFNSKLQGCNETQTEHPHCTEMETCI

>Ac_A26 c23525_g2

METVTVAEEELTTMKYVNATPEFTKEEINSFFFYETIQFTVLWILFLSIVLGNGAVL

VALSFNKARKNRMNFFIMHLALADLLVGLVSVMIDMIWRTTVTWSAGPIACKVVKYLQVV

VTYSSTYVLVALSIDRYDAITHPMNFSSSWRRARALIGCAWILSFVFAVPSVFINEETII

QGRTQCWIEMSPWQWKLYISIVATTVFVVPAIVISGCYAIIVYTIWSKSKLLSPAKNNTL

QRGAKKPEEHDIRRSSSRGIIPKAKIKTVKMTFVIVFVFILCWSPYIVFDLLQVYGYIPK

TQASVALATFIQSLAPLNSAANPIIYCLFSTHICRSLR

>Ac_A27 Unigene0021484

WEEYVDGTLVPVYRTEAITVWPVLFFVGTISAFFVVPLFVLSVLYVIIARHLMANPGTVA

PNTNRAALRYRRQVVLMLGTVVVSFFMCLLPFRALILWIILAPPDYNIMELLGVKNFYLL

LFFSRIMLYINSALNPILYNLMSSKFRDGFRRLCGLRRGPWANRHLGRKGTVTTTSAHAG

GSANGGTTTTTTTATSSVKSDGGGNDRATAAATANMYARMKRNGVTVVSAVDRSDRTAGG

RRIINRLN

>Ac_A28 c25921_g1

MSLFNTSYLMGNDTAGGWGPRYFFTFYSEFGDEQAESAVEVTVFMVIFAASVVANVSIAW

AVLRYREMRTVTNCFLLNLTVADLLFAVTTPALAYVRVRPDWPFGDFVCRLLPYSQFVCG

FVLLWTLTLISMDRHRCIVVPPYRSQLTPRRATVLTVLTWLIALAVFMPVPFWFHEQAVM

GGTAVNVCTLVFPKNDTFKMSIVFTVSVVSLSCILPLSLFVYHYQRIFHKLNKTRRRIEH

SVSHRSTAVHTASRNSLSPPTNGSTPQVLVRHEELRYRKHVRVVRVLLINVIVVLVMWLP

ITVVMCLIYVDGSRDTEDTGYFLRSHHFIMGLLFALLNTVVNPILYGVLSENFRKCFARL

WFISKRRRAMHRELLDNASKCGARTPSNGHYNSTLQPGSSASVVELPATAIVSSSATNEC

W

>Ac_A29 c23903_g1

MEDHIDDNILSNCTAMVLSLEFDKPSKELEYYNSTSLLAILGDMLKSSDLTMSKRTIARD

RLHDCVYPLPEPVHNISLWHKLTWSTVFIAMLIVAIVGNSIVIWIVAAHRRMRTVTNCYM

VSLSISDLFMASLNCLPNFIYMLNSDWAFGLELCMVSNFVAYWTVASSVFTLVAITLNRY

MAIVHPLRHRRSKTRTQTVLILIWLISVFLAMPCILYSDIKTKRYMNGELRRACYILWPD

GRYPDSKTEYIYNILFLCVTYIIPLTVMAVCYTIMGRELWGSKTIGQMTQRHVESIKSKR

KVVRMFAVVVTIFLICWLPYHSYFVYAYHNKSIVVKTYVQDLFLSFYWLAMSNSMVNPII

YYWMNPRFRAYFKLIICYCGGLRNPDKVSINLNVIRMNGESQYYLTRSKSGPVSLGVQHL

RRPPETQVLCLPRGYSEVVRHLPKTRNNQETTVIQSSRLQKLQKTRKLSTQKLIYNQ

>Ac_A30 c20833_g1

MDAMNSSADNYGLTTVGNRSSSPFPEWSGGGNWSSQNETTAILCPLFESTDDDDYNLLFEFVTYGVLLNVIGVFGILGNVISMVILSRPQMKSSINYLLIGLARCDTVLIVTSMLLFGLPVVYPATGHLFNYYFKVYPLIAPVVYPIAMISQTVSVYLTLTVTLERFVAVCHPLRARSLCTYGRARAYVVATIAFAVFYNVTRFLEVTVQKCMHTGSNQYVYQVYPSDLRNDHNYISIYIHWMYLLIMYFIPFGSLAVLNAAIYRQVRRANRERQRLSRLQKKEIGLATMLLCVVVVFLLCNVWALISNVVEAFYGIIVDHLVKVSNLLVTINSSVNFVIYVIFGEKFKRLFFKLFFPRGVWMCGWQLATDGRGGPGCEGGGGGHVAMDDSEATCNGATAFECRQLGTGTGGSTSYTDHFGRSRRGRHHHQHHHHHHHHRDGDLLDRNQMANSVGGCGNSVNGTSDDRELCLKTPTNSGMMWEHSTTTTTTTVNVHQF

>Ac_A31 c25336_g1

MVCSLDETMSTFMEACNDTEMNSTFNFSLEEVYSIMLEHRRNSRNLDKSTETLLIIVYLG

LMIVGLSANLTVIYVVARRAQMHTSRNLYIVNLAVSDMTLCLVCMPFTLTSILRHQWSMG

TILCKLVPLLQGTNIMVSVGTITVIAIDRYWVIVRGSAQNERRTVYVSIAIVWLMAVLTT

SPVAYYQVVEPLKFQHVVIYESCREKWPSTDIKVAYNIAVVLIQAVLPATVLLVVHIRIA

AYLHAHTASQKDSRRAQRELQRNKRTTLLLIGVAVVFTVSWLPLAVFSLAADLMTKPITT

KQLYVTLAVCHLTAMTSAISNPIIYGWMNSNIRNELFQLFYTKILRRRPGNRSTATATTT

MRNRTRPLITYNTSNYMPGSQETFSKGVTVL

>Ac_A32 c23253_g1

MDMMDSDANTVLHSVTKGVHVGPPPVWPGMDNVTNSSMFDESNLPYDMKFNEGHVVAIVT

YSILMVVSAIGNITVLTIILKRRRKAGTRIHAMLMHLAIADLLVTFLMMPLEITWAWTVQ

WILGDPLCRIMSFFRIFGLYLSSFILICISVDRYLAVLQPMRLYQMDRRGKLMIAVAWIA

SVICSLPQSYIFHVERHPNATWYEQCVTYNAFSSKLHELAYLYFGMFMMYWLPLIVILFC

YASIIIEIYRRSRESICGQGTDNVRRLGFLGRAKSRTLKMTIIIVIVFVVCWTPYYIMAI

WYWTDHKSAQMVDQKVQHALFMFACTNSCMNPIVYGAFNIRTRRTLVTQGVGESVASVRV

VTWHKLTVRSKANRKSTIGNKSTLFKSVHNGNTNDDTLVNENVTVTTTLCNDNSDINDKN

I

>Ac_A33 c20484_g1

WPGLYGPNGTFDGGGDVDGVGAD

GGLGGFDGGFRFNSSVKLALDVIFDNQLNVDKVLVMYLEVAIVVAYGVLFVIGLVSNGLV

CFVVFRQCGKKNVPSQGPSPRNLYIVNLAFADIIMCVVCMPFTLWALMSRRWTWGLIMCK

TVPAAQGANITVSACTITAIALDRYFTIVRNPRGSVFRCSVAKTLVLIWLVSFAAMVPLL

LYQNVEEVHVGPIRLYEACVEKWPSRVAQQTFTVGLAVAQFVLPFTTISLIHLKISSYLT

VHLKHPTIPSKEINCRRVRRELSRNKRTMWILSSIAVVFALSWLPLTLFTLLVEFQPHLI

DSSDSLYKAFAIVHMMAMSTTCTNPLLYGWLNTNFRRDISGICRQMWYCCGDQKPPPRRR

RYPSVATDYRGPNDRRMGMGSEAAGGCGGGGGGGSGGGGGGSRRHQMHHDPETTGMSMVV

KSDRRFSTSYLTTLTTVTSRSQPGSSMGRVENV

>Ac_A34 c23906_g1

MLSEETLTNDTFYNDSI

SYGNNYSSHDDHQWGPRRDSLPVVVPVTLIYTLIFATGIVGNVSTCIVIARNKYMHTATN

YYLFSLAVSDLLLLLSGLPQEIYLTWSRYPYVFGEMFCIVRGFAAETSTNATVLTITAFT

IERYVAICHPFLSHTESNLSRAFKYILIIWVVALTLAFPQAIQMGLVYARTQDGAIIPEL

STCGLANKLPYAFELSSVLFFLAPWTLIIVLYILIGLKLYKSKRDSSRTTCSAHCRHTAC

LSSTNTRATGRVVKMLVVVVVAFFVCWAPFQAQRLVAIYGYANHEDHSSTSIIDANVYQV

LNYVSGILYYLSATINPLLYNLMSYKFRTAFKETWRSCKGASGISPDHSMPNLDYKWQRH

HSHSRSFSSSHFDEVNSMKRLMVANKSWQPTIVAINETSDTGTSSAAEKTLRFPHKMIFS

KQDAIKENGTVMENGITIGVLTNNNRTTTMLMTPN

>Ac_A35 c23752_g1

MSLQNELYIDGDGKGNQLNLTAENTWDNDYIMPKRDPLYIVVPMTIMYSVIFVTGVIGNS

ITCMVIAKHKYMHTATNYYLFSLAMSDLILLVSGLPQEMWSIWSRYPYVFGEIFCQLRGL

FSEMSANATVLTITAFTAERYVAICHPFMAQSMSKLSRAVKLIIIIWLVAVLFAIPQALQ

FTVSSWDGSSELMQCNIRSILLMDTDIELSTVSFTLSTMLFFLLPMTLITVLYALIGLRL

RRSDKLKRTITVRSTHGEKKMSSPEYRANSSPKVLKMLVAVVVAFFICWAPFHAQRLIAI

FGISHASSGNLDAKDIPFLHQLYGISTYISGVLYYVSTTINPILYHIMSLKFRGAFKCFL

LCTDRKRCYGVVAATAVAAATDGPENPNNNVRHVGRGGGCAATRPNLPPMTADQSTDEYP

DERHRPSNYRRPPTMTTTTKFAVAQVPAPTPSSPIVGGGR

>Ac_A36 c27060_g1

FKFSLLILLLLIQFDIGESQTGIKNNSTTVPTLCNQCLESDSCQLNSTSDIYHCDETYCI

PKSFVCNGIPDCFRGQDEAVSECGCLQNEYRCKNKCIELVKRCDKIADCDEGEDEIDCKT

HVCPTTHFKCANYFCIPTDKTCDFKDDCGDGSDELQCKHRECWHGEFKCKNSECIRPGYL

CDGEVNCVDGSDEENCEISDFIKCGGSHSVHSTFWCDGWPECIDNHADELLCNISCSENK

FQCPNGRCINDANVCDGQCDCLQSIDGDCADEMNCTAFYNKTDDVVVCTTGSTLSCWMPE

GNPSRCIRQKYICDGQNDCFNGFSISDEFGCDKPNSHLNDEFFKCKDGRWLPFKHRCNFK

AECLDGDDETDCEVSLPCDEDQFRCASGECVKSENRCDGRTDCWDKSDEIGCMTVPCPSK

HWRRCEIGKQCVPIEKWCDYAVDCMDGSDEKNCQYRLCKNDEFQCDSGQCIPLEYKCKKY

QEEQMGCVDKSHLRNCDDSKCAENEFKCHRGPCIHQSMVCDGKLDCDLTWDDEDNNCYFM

CSDIASGCQCQDVHINCTGHGLDQFPYDVEKEITFFHLGGNNFSESLHENTFQHLDRLVH

LDLTNNSIKHLEPFLFSTLWRLKTLNLQNNKITILQNCSFMGLGQLTGLHLQGNNIYKLS

SMAFRGLSSLITLDLSNQNITDIESEAFVGLRSLKSLDLSYNSLTYIRDGTFRGMPQVVF

LNLKNNQLRVIDKNVFFTMPLLETLFTDEFRFCCLARYVKHCEPQPDEFSSCEDLMSNIV

LRVCIWILAVVAITANLLVIVFRAKYKHTNQVHSFLIVNLALGDFLMGSYLLVIAVVDWY

YRGVYFIHDSDWRQSSMCNVAGFISTFSSELSVFTLTVITLERLLVIIFPFKVRRLEMDF

TRILMAVCWLLAIIISAIPLFNIHYFRNFYGRSGVCLALHITPDKPNGWEYSVFIFLFVN

LGSLLLISGSYLWMFFVAKMTHRATETLIRHRSLSESAMAWRMSLLVATDAACWVPIIGL

GLWSLAGFTVPPQVFAWVAVFVLPLNAAVNPVLYTLSAVPIIRRSVVSNRRAVSLRRSVT

NDPHPHN

>Ac_A37 c26277_g1

ETSIQNLPTAGLEELDVLKIEKTYTMNVFPSIYNFKNIKEAWLTYPYHCCAFHFPKTHNP

QGYANHEKLQQKMMHECKNIPFVDPISTVKTMHSTTENFIGDEMFHSGDVTIGTKVDVIC

GNVSKNYLEVKCYPEPDAFNPCEDLMGNWTLRVAVWIVAVAALLGNMAVLFVLLSSRFRL

TVPKFLMCNLAMADFCMGLYLLLIAVMDARSIGQYFNHAIFWQRGIGCKVAGFLTVFSCM

LSVFTLMIITGERWYTITYAIHLNRRLKLGASVNIMAVGWLFSLVMGALPLMGTSGYSKT

SICLPMDNSTFADKVYLFSLLTFNGMAFVLICACYAKMYASIRGGREAVASVARSDMTVA

KRMALLVFTDFACWAPVAFFGLTALAGYPLIDVPKTKILLVFFYPLNSCANPYLYALLTQ

QYRRDLFVLLSRFGVCSRRADEYKGTGPVACGRRKAHCNDAQRHRDRRGYGGGGGG

>Ac_A38 c17409_g1

FKEMKDLSSLNMNGINEENIDFSSFNNLTTRLEVLYLDEFHYCVFHAQHVKLCFPNIDEL

SSSLNLLPNSTFRWFLWMVTVLILVFNSIVLYGRMFNVFKYDNKALNFVIRNLAVADLFM

AIYLIVICYHDQIYRNEYYLYAHKWESSNLCTMVGIMAVISSEVSMLILVFLSLDRYIII

GLHFIGNPGLKMKTAVFTMASIWVVGISLSVAPLILWKHSSKFYGSNGLCYPLYIEDPFV

SGWQYSAFIFLGLYAISLLLMTVLYALLFKNIKETRKRAKRISTGDFDLTVRFFFIVLAN

ILCWSPIIVLKLAALRKY

>Ac_A39 c22388_g1

DLGYIDYANDSEEEFFNEPAITNKTQDIICGNITPPNMESDVKCWPQPNALNPCEDIMGF

YWLRVCVWLVGIAALLANVVVLLVVFRKKFNYSVPRFLMSNLAFADFCTAIYLLLLAYED

LVSSEKYFNYAYTWQNGVGCKIGGFLTVFSSQLSVFALCLLTLERWYSIRRALYTNKMTF

ASTVRIMAFGWVYSIVMATMPLLGISSYSTTSICLPMDTARASGMCYVFTLLTFAAAAFL

LMLFCYVQIYMSLSYETRHAAKGEASVARKISVLIGTNFACTAPVIFFGFTALLGYPLID

VTKSKILLVFFYPINSCANPFLYTILTASFRREAFSTVTKFGLCLENEKEYKVIYSTQTN

NTQRLTPVHKHTLSLTLPQP

>Ac_A40 c25922_g1

MISALLTALSLMILSLASADVN

FMQSCESPAQCRCDGNEATEVSCRNLRFTRIPDDIPVNITKLDVASNNITEIDEHVFSRL

TLLEDLVLADNPIKEIHPGAFLNNIRLKRLSFQKCQLVRAPCETFQSFRQLSSLQLDQNH

LTEIDDRCFDQLSQLRNLRLENNKLTKVPKQALSLVPTLEALNLGSNSIVDISNDSFSSL

PNLVILLLKRNQIEFVDETAFESLTSLKILELDDNQLDTIPVALAKLTSLQELSLSGNNI

KFVPEGVLQRSQGLALLELKGNPLIGVHPYAFASLPKLRKLVLSEARELTEFPNLNGTSA

LEVLRIDRASIYSIPDTLCTTCPKLKSLDIKWNRLSRIPNLNKCKELRVLDLANNHIASL

EGSLFRNLSHLHDLLLGHNYITSIPRDAFQGLVQLKVLDLESNKIDRIDDETFLSFSQLE

DLNVGKNVFEHLPTKGLERLLHLKTFNNPNLRQFPTPEHFPRIQSLVLSYAYHCCSFLPL

QSEDDSPPSSQTSLHESVIFPANENDFDMTLWNSSMSDIWPQLQNLSKKFGTQVNDLWDS

FGSDFTYPGNLPSYMEEYFEEQQLEGRSQDVMSSKIKCLPLPGPFLPCQDLFDWWTLRCG

VWVVFLLAMLGNGTVVFVLIFARSKIDVPRFLVCNLAAADFFMGLYLGILAVVDAGTLGE

FKVFAIPWQMSAGCQLAGVLAVLSSELSVYTLAVITLERNYAITHAMHLNKRLSLKHASY

IMLCGWIFAGGMAALPLFGVSDYRKYATCLPFETTTGTWSLVYVVFLMFINGVAFFILMG

CYLKMYCAIRGSQAWNSNDSRIAKRMALLVFTDLLCWAPIAFVSLTAICGFHLVSLEQAK

VFTVFVLPLNSCCNPFLYAILTKQFKKDCVMICKAIEESRVTRGIGRCRHSSNFSNRQTP

ANTNSLVDRSSRDNYPHPPCSCNVKLLDDKSVKTEETFYDWINIKFRRIMSCLQRESDSG

RQYMRSDRYAYQIAEIQQKQHKRASSMSSSENYSSSRSDSWRQHHHHHHHHHHCGVPLRL

LDPKQRRASSWIVTRKTSQDSNLSSSRNDSSGSANTASTSVSTRTSRSSVGCSDHKPKPR

LTRQPAVLDDPELVVGGSPNRLSVRFLATIPSAAETSIYRADTEDEDDENRMDDSLRPEN

SPTTTASERTTPPDSKPSSPHS

>Ac_B1 c24053_g1

MTTESPTGISAE

FQNHLRSECEHKLNETVFTHPSSNFCRGTFDGWLCWPDTEAGDTVHQPCPEFISGFDPTR

TAYKICNKNATWFKHPISGAVWSNYTTCINHEDYNWTQQINNIYQTGYLVSFIALLLSIA

ILTYFKSLRCARNTLHTHMFTSFAINNLLWLLWYRLVVEHPSVVLHNGWWCQILHVVLHY

FLLTNYAWMLAEGFYLHTLLVFAFTSEDTLVRWSWTLAWSTPLVVISLYTVLRISNHHTS

ECWINESPFTEVLVVPVCMSMALNLVFLCNIVRVLWVKLQAGPSHLSNSTPSRTLLQAFR

ATLLLLPLLGLHYLLTPFRPPKQHPWEPFYEVVSATTSSFQGLCVATLFCFLNGEVVAQI

KRRWQFMFFRTRANSYTATTVSFVRSTAGPGDNEDKV

>Ac_B2 c17700_g1

MNRMTTNILTSCDSNSSYNLITSNDKFCNWFYDEKTCWPIAKANTNVSQPCPKEQGFDKM

KMAYKYCTEDGNWENVTNLFGSTDYSSCYTPELTEMLEKLGSDYDTKRKLNIAINTGVIE

VVGCLVSIISLIISLCIFIRHKGLLEQRFKIHMNLFVALSLQMFIKLILNIDKLDLFKTK

KYHSNVTCLVDCNNQIRLVKDDDNWFFGIMYTPIICEGSQVILEWSKTASFMWMYNEGVY

LYRIIKSRVLRINSINFRMYLFGWGLPALMTSVWLLVTMVYYKKSSSVCWWGYSLLPFFW

ILEGPRMIIIVVNFIILIIVLRELLKKIKSKLSPSNELDIIRKVSKAAIALVPLLGITNF

ILVILPEPHSKSPELFAFWSYSAHILHSFQGLMVSILYCFMNKEIKKSFDKRMMLQKSRR

NFEMEMT

>Ac_B3 c25414_g1

MADYQYGLSSIQDNHILHQVIAERRQICESIINDPPVSDSNDWCPGEFDGWTCINRTKAG

EVAKFPCPYFILGFDTKRFGQKTCLPDGSWFKHPDSNKTWSNYTTCVDLEDLKLRNQVNM

IYKWGYTVSLAALAVSIFIFFYFRSLTCTRIQIHKNLFISLAVNNCLWLVWYEAVVDNLP

VLMTNGLGCKVLHVLVQYFLVATYFWMFCEGLYLHTLLVVTFLTESRVMPFLHTIGWGIP

ALLVSTYAVLRTATPGETLHCWIHESLYSWILSGPVCLSMLANLVFLINIVRLLLVKLHA

RQITMSSPKHAVPRERAQSFSLRSFKRNNSSVDSYDKAPATSSRTGKAVRATLILIPLLG

LQYIVTPFRPEPGTSWEPVYQVTSAVVASCQGLCVALLFCFFNGEVLSEMKKRWKHCWIN

KNRSWNPCLRVTSVSSPQNHPRLLVTEDQQTQRTGSIQVLPRKDSAECLQNVQL

>Ac_B4 c24744_g1

MSDPAGARVHDSNTLFLIELRQKCFKN

AKPVNVTQKFCPRYFDGYACWEETLPNVTAFAPCPNYVVGFDPYRTAFKSCLSDGTWYRH

PDTGKQWANYTSCVDVDDYEFRDLINKLYIFGYSVSLAALLTSLLIFFTFKTLRCTRIAI

HVHLFISLALNNLTWIIWYKTVVQDLSVVQQNGIYCRSLHVALQFFMVANYMWMFCEGLH

LHMALVVVFVNDVYAMRWFYAIGWGAPAVLTILYVSWRSNSEDTTQCWMHESHCQWVLTV

PVFASILTSLLFLMNVMRVLLTKLHRNSTNPAPIGVRKAVRAALILVPLFGIHYILIPYR

PKHKTTVEIIYQIFSVILVSTQGLCVSVLFCFANVDVHGAFHKYIRRIKRRGTTHTNVTG

TTQPVQSQIRDAIV

>Ac_B5 c27642_g1

MNITTTNNTCPPEKVLNPGWCPNHWDLLLCWKASKPGAVVYQACFDELNGVRYDTSRNAS

RRCKSNGVWENSSDYMNCKPLDTHSSLYPDPSIVYTSYFYYGGYTISLVALVAAVSIFVY

FKDLRCLRNTIHTNLMCTYILSDFTWILTSTLQEWLSASNNACVLFTFSLHYFVLTNFFW

MFVEGLYLYILVVETFTRENIKLRVYMSIGWGIPLVIMIIWGVSKIITPMEIEERSDSCS

WMTPHPVHDWIYQGPAIIVLIVNLVFLSKIMWVLITKLRSANSAETQQYRKASKALLVLI

PLLGVTYILTMVGPTESGTYANYYSYGRATLLSMQGFMVAIFYCFVNSEVKNTFKHHFAR

WNEARNLRTRGSRRFTYSKDWSPNTRPSVHIGEKKTTGCA

>Ac_B6 Unigene0036280

MDYLNDSWSPNEEAALKCSSWYEWDDRWTNGCNASSDSLICWPPTPAGVIVYQPCFQELR

GILYDTTKNASRICFENNTWGLTDFNECTILGEPKAVTYDEEDTVDTIIYLYIAGHCLSL

IMTSLAIFVFCRFKELKCLRNKIHSNLMASYLLAGIMWILNYTNLTDTGTFKCALLVLPL

YYFTMTNYFWAFIEGMYLFILVVDTFFPDRVRLRTYMAIGWGIPLIIIPTWCVTKLLVPT

KNDLDLYNQITYERYCPLMASYVDDWIYQSPIVIVLLINSVFLVKIMRVLITKIRSTKSA

ETHNYKKATKALLVLIPLLGITFCLDMINPSSTGLLVNIYKFSKVVIISTQGFTVSLLYC

FFNNEVQSTLKYHITRWQTKRKFLASRKKYGRSWLSVKQNNIICDHQLDPKELMPWIPAN

NVRSSSCVSNDTTTSALSNMRLPPDTTIEIPPHPEQQPLYDEDTKYVEDNREP
