# Supplementary material for: Identification and Characterization of Neuropeptides and Their G Protein-Coupled Receptors (GPCRs) in the Cowpea Aphid Aphis craccivora
Source: Front Endocrinol (Lausanne). 2020 Sep 17;11:640. doi: 10.3389/fendo.2020.00640 (PMC7527416; doi:10.3389/fendo.2020.00640)
Supplement: Supplementary file 4 [file Data_Sheet_4.docx]

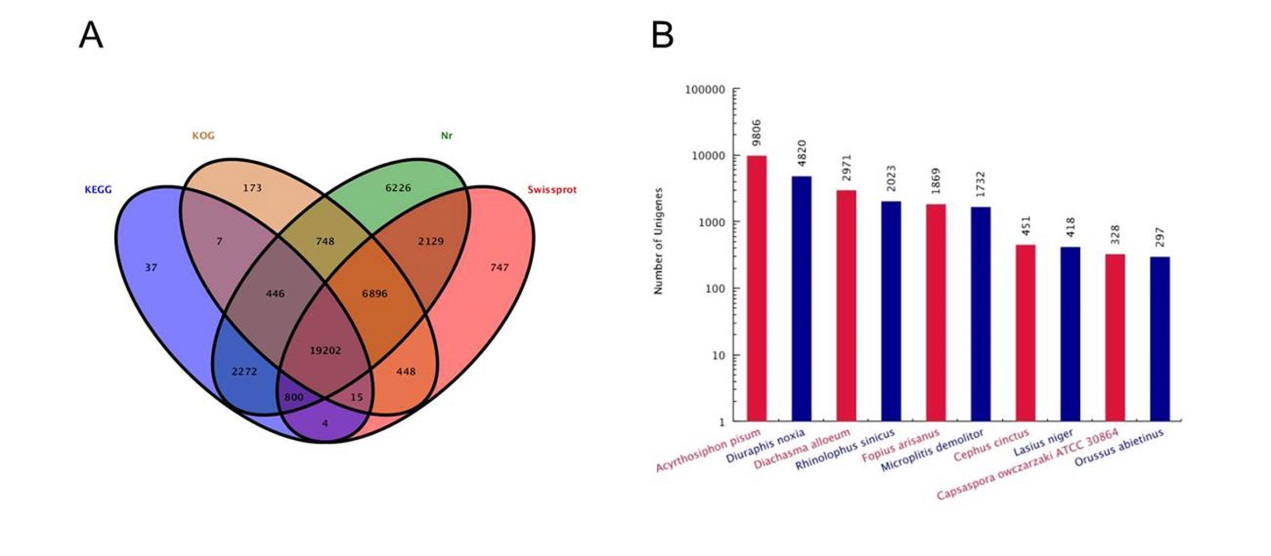


Supplementary Figure 1 Multiple Venn diagram of annotation results (A) and top species distribution for Blast hits (B).


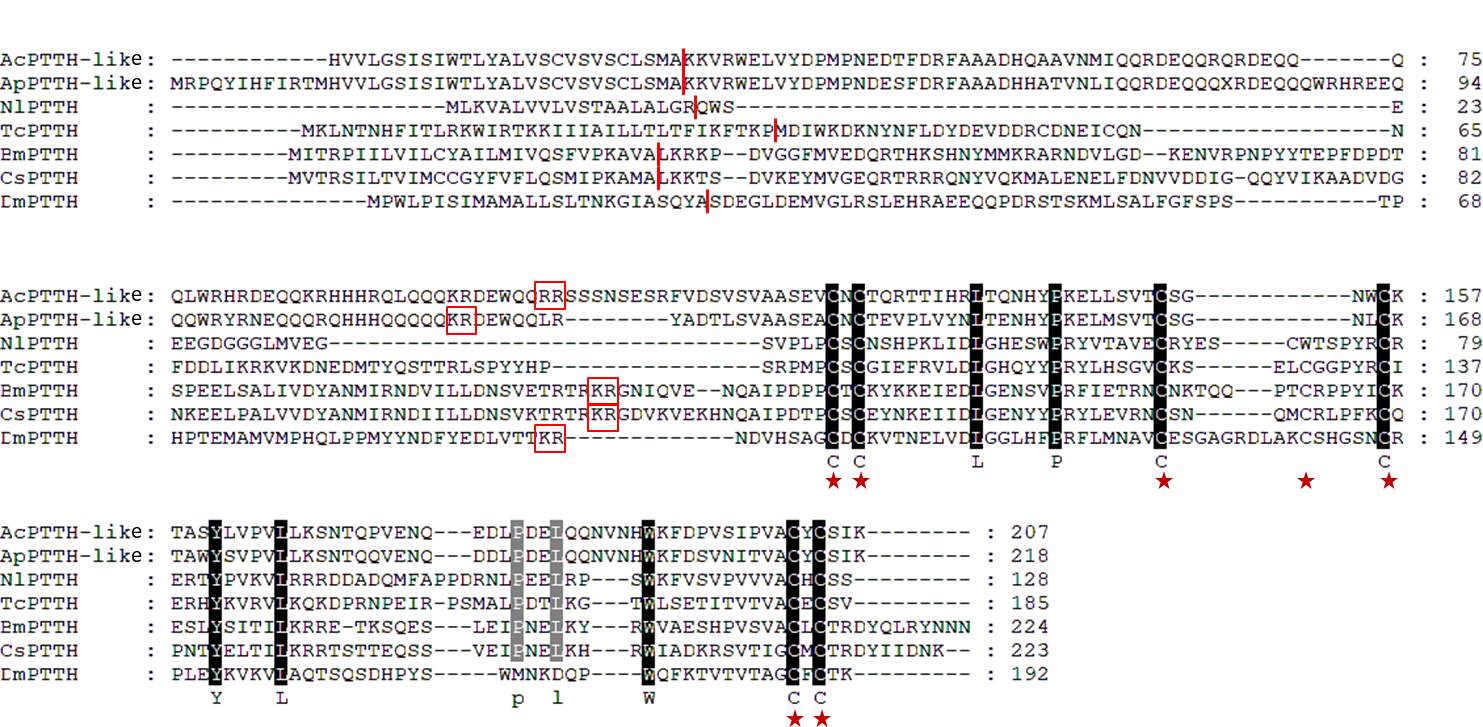


Supplementary Figure 2 Multiple alignments of the prothoracicotropic hormone (PTTH) precursors identified from *A. craccivora* and other insects. The cleavage sites of the signal peptidase are marked by vertical lines and proteolytic cleavage sites are boxed. Amino acid residues that are common in all seven sequences and in six sequences are highlighted in black and in gray, respectively. The conserved cysteine residues are marked by asterisks. Ac: *A. craccivora*; Ap: *A. pisum*; Nl: *N. lugens*; Tc: *T. castaneum*; Bm: *B. mori*; Cs: *C. suppressalis*; Dm: *D. melanogaster*.
