# Supplementary material for: Identification and Characterization of Neuropeptides and Their G Protein-Coupled Receptors (GPCRs) in the Cowpea Aphid Aphis craccivora
Source: Front Endocrinol (Lausanne). 2020 Sep 17;11:640. doi: 10.3389/fendo.2020.00640 (PMC7527416; doi:10.3389/fendo.2020.00640)
Supplement: Supplementary file 5 [file Data_Sheet_5.docx]

Table S1 Primers for RT-PCR

| **Primer name** | **Forward primer (5` - 3`)** | **Reverse primer (5` - 3`)** |
| --- | --- | --- |
| c19757_g1 | TGAACAATACGACGAGTGGA | AAACGCAATGACGACAGC |
| c21909_g1 | GACGGATAAATAAAGCAGAG | CAAAGATGGTTCAAGGGTA |
| c27060_g1 | GGTTCGTATCTGCTGGTG | CATTGTGCGATGTGGGTC |
| c21429_g1 | CCTAGCCGACCGAATCAA | TCAACCCAATATGCGTGC |
| c20484_g1 | GACGACATCGGCGGCATT | GTATCAGGTGCGGCTGGAACT |

Table S2 qRT-PCR primers for expression profiling of genes encoding neuropeptides and their GPCR in *A. craccivora*.

| **Primer name** | **Forward primer (5` - 3`)** | **Reverse primer (5` - 3`)** |
| --- | --- | --- |
| RPS8 | AGTCATAGACGCAACACCATTC | GCTTCCCAAACATGCTAATAGAC |
| AKH | TGGTATAGTGGCGTTGGTCAT | TGTTGTTGTTGGCTGTGTTCA |
| AstA | CTCTTCCTCTCCTCCGACAAG | CGCACAAGCAGTACGAGTTC |
| AstB | CCGCCGCCTTATACGCTAA | GGTAGTCCGAGTTATCTTGTTCAG |
| AstC | GGAAATCATCTTTATCGCCCATTG | CACTCGCCGTCCTAACTGT |
| AstCC | AACGCCACCATTAGTCTATCCA | CCTTCACATTACTCAGATACGACAA |
| AT | TGGTCCGATTGTTGGTTATAGAG | ATCCGATGGCATTAAATTCACG |
| Burα | GTCGTAATGTCTACCATCAACCA | GCTTCTCGTTCACCACTCTC |
| Burβ | GCCATCAGGATCGTAGCAATG | GAAGGAGCGTGTAATTCACAAGTA |
| CAPA | CTTCTATCTCTGTTACTGTCTCGTT | CATTGCGTATTCGTCCGTTCT |
| CCAP | CGACAGCCATGAATCCATCAA | GACATTATCTGCCTGCTAATCTCT |
| CCHa1 | CCAAATCCAGAACAATCACAAGAAG | CAGGTATCAATGGTGTCATCGTTA |
| CCHa2 | CGATTGGTTCCGTCTTCACAT | CGTCGTCACCGCACTTTAC |
| DH31 | GAAAATGACGGAGACCCTG | AACCCAAATCAAGCCCACG |
| EH1 | TTACACGGCTGCGGACAT | GCTTGTTGAGGAACGGTGAA |
| EH2 | CGTGGCACCGTTCCTCAA | GTACAGTCGCTTGGTGGATT |
| EH3 | ATGTCCGCCGCCGTATAAC | CAGTGTATCGTCCGATCTTCCT |
| ETH | CCAGGATCGGTCGTAGGAATA | TTGATGTCGTTCGGCTGTAAG |
| FMRFa | TCGGCATCGGCAGTCAAG | CGTAGTGGCGTCTGGGTAT |
| GPA2 | TGGGTATGCTTATCTTCCTAG | GATGTTCTTCTTGGCTGTCC |
| GPB5 | TGAAGGACTGCGACGAAGG | GGTGGAAATGAACTGGAAATCTCT |
| ILP1 | TGTGGTTCTAGTTTGGCAAA | GCTTCTTGTCTTGATTGGTATG |
| ILP4 | GACAACATTCGTCGATGATACTTC | GCAGCGTTCACCTCAACAATA |
| ILP5 | GATGTTACCGTCCCAAGC | CGGTGTTATTCCCGAGTTTC |
| ILP7 | CCGAAAAATGGGAATACCACTTA | ATTCTATTGTTGACTTGGTGC |
| ILP8 | CCAAGCATTAGCCGATGAACTA | ACAGCATTCGTTGACAATACCT |
| ILP10 | TATTGTTCACGGAGGATTCTCT | ATTGGTCATAAAGGGATGTTCC |
| ITPL | CAGTGAGTGGAGTTGTGGTTC | GATGGCGATAATGGCTGTGT |
| LK | TGGTTGCTGCTGGTTCTAATG | TCGGTGCTAGGTTCTACTTGG |
| MS | GTGCGTGAGTAGGTGTTAG | CAGCAGCCCTGGCATACA |
| NPF | CGTAACATCTTCTGAGGTGGAA | GTTGTAATCTCGGTGTTGTGATAG |
| NPLP1 | GTGTTGGAGCAAGCAAGTGA | AAGAATCGGATGTGGTCTAACG |
| NPLP3 | GAGACCAGATGTGATGATGTTGT | CACCAGCACCAGCCTACTT |
| PBAN/PK2 | CTGAGTCGCTGTCCACAAC | CGCTACCGCCATAGTCGTA |
| Proc | CCGACAGTACCACACCATCA | GTTCACGCAGTAATACCTACAGT |
| PTTH | CCGATGCCGAACGAAGATAC | GAACCTTGACTCGCTATTGCT |
| SIFa | GACAACATATTCGCTCTTCAGAAC | CATCAACACTACCATCAACAGGAA |
| sNPF | TAACCTCAGCGACGGAGAC | CCACATCCACAGTAAGTCAGG |
| TK | GGAGAGTTCCGACTTCAGTTC | AGAAGGACTACTACGGCAACA |
| A1 | CACCAGGACACCAACAACATT | GGCTCGGAACCAGGATTGA |
| A2 | AAGCGAGTGACCCGAATGG | CGCAAGACAATGGCTGACTA |
| A6 | GCACCAAGACCAGAGCAATG | ACGAAGAACACGCCGATGA |
| A7 | GTCGTTTGTCGCACGCACTT | GTCGGTCCGTCGCATCTT |
| A8 | TGGCTTACTGACGCTGCTA | GTGCTGCTCTTCTGGAACTT |
| A9 | CTACAGCCACACGGTAACTTG | CACGGTCTTCTTGAACTCCTATT |
| A10 | TCCGTTGGCTGTAGTGACG | AATGGCGAAGTTGATAGGATAAC |
| A11 | TTATATGATGGTTGGCGTTCCT | GCTGAGGTTGATGATGAATACG |
| A12 | AACGACACGACCGACTTCA | CAGCAACACCATCAGCGATAT |
| A13 | ACAAAGAGCGGCAGACGG | TCCAGGACGGCAAGAAGAG |
| A15 | GTCGTGAGTTGTGGTCGTATTC | CGTGTCGCTGTTGTTAGTTGT |
| A16 | CTATACGGATGAGGAATATCGCAAT | TGGTGGCAAGCAGATTAGGAT |
| A17 | ATTCACCGAGACCAGAAGTATCA | CCATAAGCCAGTCACCGATTG |
| A18 | GCGTGTGGCTAGTTGTAGTG | CTGTGCTGTTAGGCGTTGAA |
| A19 | TGTTCTGGTGGCTCGTCAA | CTATGGTGGTGGAATCGTTGTT |
| A20 | TTTTGGTGTTGAGGGCGTACG | TCGGGTGTTCATGCAGAAGTAG |
| A21 | GCATCAGGAATGCGGACTTTA | GTAGAACAGCGAATGGTATGC |
| A22 | CAAGGACATCAAAGACCCAATG | GCGCTCTATACACGAGCATC |
| A23 | GCTATTCTCAGTCCGCTCAG | GCCGTCTCTATCTCCGTATTC |
| A24 | CGTTGCCGTGCTGTCCATA | GCTATTCGTAGTGATACCGTCTG |
| A25 | ACCGCTAACGAGACAGTAGAG | GCAGTCCTAAGAGTAATAGTGTGAG |
| A26 | CAGTCTTGTGGATATTGTTCTTGTC | TGCGTGATGGCGTCGTAT |
| A27 | GGCACCATATCGGCGTTCT | GGCAGCAGGCACATGAAGA |
| A28 | ATCGTGGTGCTGGTGATGT | TGCGGAAGTTCTCGGACAG |
| A29 | CAGACGAATGCGAACTGTTACTA | TGACCTCCTATGCCGAAGTG |
| A31 | CTGTTCTATTGGTGGTTCATATTCG | GTTGTTATCGGCTTGGTCATCA |
| A32 | CGCCATAGTCACCTACAGCATA | TGTATCCTCGTTCCAGCCTTG |
| A33 | CCGCACCTGATCGACTCTT | CCGCAGCAGTACCACATCT |
| A34 | ATCGGGAATCTTATACTACTTGTC | GCTATGGCTGTGGTGTCTC |
| A35 | TGTCGTCGTGGCGTTCTT | GTATTGGATTGATGGTGGTGGAT |
| A36 | GAACCGCAGCCTGATGAATT | AAGTCGCCTAACGCCAAGT |
| A39 | TCTGCTGATGCTGTTCTGTTAC | CCGTGAGTATGGTGTAGAGGAA |
| A40 | GGACTGTGGTATTCGTGCTTAT | GCGTGCGTGATTGCGTAA |
| B1 | CTCCATTATCTGCTCACACCTT | TGAATTGCCACCGTCTCTTG |
| B2 | GGTAGTCAAGTCATACTGGAATGG | CACCAACAGACACTAGACGATT |
| B3 | CAGTGTGGACAGTTACGATAAGG | CTTGACAGGAAGCGACTACAG |
| B4 | CTTGGTTGTGGTGTTCGTCAA | GTGGTGTCTTCAGAATTACTCCTC |
| B5 | ATCCTGGTTGGTGTCCTAATCA | CGCACTCCGTTCAATTCATCA |
| B6 | ACTCCTGCTGGTGTAATCGTAT | CAGTGTCTTCTTCATCGTATGTCA |

Table S3 Neuropeptide genes in *A. craccivora* and other insects

| **Neuropeptide** | ***A. craccivora*** | ***A. pisum*** | ***D. melanogaster*** | ***A. mellifera*** | ***T. castaneum*** | ***B. mori*** | ***N. lugens*** | ***R. prolixus*** | ***D. citri*** |
| --- | --- | --- | --- | --- | --- | --- | --- | --- | --- |
| Adipokinetic hormone (AKH) | + | + | + | + | + | + | + | + | **+** |
| Allatostatin A (AstA) | + | + | + | + | nd | + | + | + | **+** |
| Allatostatin B (AstB) | + | + | + | nd | + | + | + | + | **+** |
| Allatostatin C (AstC) | + | + | + | + | + | + | + | + | **+** |
| Allatostatin CC (AstCC) | + | + | + | + | + | + | + | + | **+** |
| Allatotropin (AT) | + | + | nd | + | + | + | + | + | **+** |
| Bursicon alpha (Burα) | + | + | + | + | + | + | + |  |  |
| Bursicon beta (Burβ) | + | + | + | + | + | + | + |  | **+** |
| Capability (CAPA) | + | + | + | + | nd | + | + | + | **+** |
| Crustacean cardioactive peptide(CCAP) | + | + | + | + | + | + | + | + | **+** |
| CCHamide 1 (CCHa1) | + | + | + | + | + | + | + | + | **+** |
| CCHamide 2 (CCHa2) | + | + | + | + | + | + | + |  | **+** |
| CNMamide (CNMa) | nd |  | + | + | + | nd | + |  |  |
| Corazonin (Crz) | nd | nd | + | + | nd | + | + | + |  |
| Diuretic Hormone 31 (DH31) | + | + | + | + | + | + | + | + | **+** |
| Diuretic Hormone 44 (DH44) | + | + | + | + | + | + | + | + | **+** |
| Eclosion hormone (EH) | + | + | + | + | + | + | + | + | **+** |
| Elevenin (Ele) | nd | + | nd |  | + | + | + | + | **+** |
| Ecdysis-triggering hormone (ETH) | + | + | + | + | + | + | + | + | **+** |
| FMRFamide (FMRFa) | + | + | + | + | + | + | + | + |  |
| Glycoprotein hormone alpha 2 (GPA2) | + | + | + | nd | + | + | + |  |  |
| Glycoprotein hormone beta 5 (GPB5) | + | + | + | nd | + | + | + |  | **+** |
| Insulin-like peptide (ILP) | + | + | + | + | + | + | + |  | **+** |
| ITG | nd | + | + | + | + | + | + |  | **+** |
| Ion transport peptide (ITP) | + | + | + | + | + | + | + | + | **+** |
| Leucokinin (LK) | + | + | + | + |  | + | + | + | **+** |

Table S3 (Continued)

| **Neuropeptide** | ***A. craccivora*** | ***A. pisum*** | ***D. melanogaster*** | ***A. mellifera*** | ***T. castaneum*** | ***B. mori*** | ***N. lugens*** | ***R. prolixus*** | ***D. citri*** |
| --- | --- | --- | --- | --- | --- | --- | --- | --- | --- |
| Myosuppressin (MS) | + | + | + | + | + | + | + | + | **+** |
| Neuroparsin (NP) | nd | nd | nd | + | + | + | + | + | **+** |
| Neuropeptide F (NPF) | + | + | + | + | nd | + | + | + |  |
| Neuropeptide-like precursor (NPLP) | + | + | + | + | + | + | + | + | **+** |
| Natalisin (NTL) | nd |  | + | + | + | + | + |  | **+** |
| Orcokinin (OK) | + | + | + | + | + | + | + | + | **+** |
| PBAN/PK2 | + | + | + | + | + | + | + | + | **+** |
| Pigment-dispersing factor (PDF) | nd |  | + | + |  | + | + | + | **+** |
| Proctolin (Proc) | + | + | + | nd | + | + | + | + | **+** |
| Prothoracicotropic hormone (PTTH) | + | + | + | nd | + | + | + | - | **+** |
| RYamide (RYa) | nd |  | + | + |  | + | + |  | **+** |
| SIFamide (SIFa) | + | + | + | + | + | + | + | + | **+** |
| Sulfakinin (SK) | nd | nd | + | + | + | + | + | + |  |
| Short neuropeptide F (sNPF) | + | + | + | + | + | + | + | + | **+** |
| Sex peptide (SP) | nd | nd | + | nd | nd | nd | nd | nd | nd |
| Trissin (Tris) | nd | nd | + | nd | + | + | - | - |  |
| Tachykinin (TK) | + | + | + | + | + | + | + | + | **+** |
| Arginine vasopressin-like peptide (AVPL) | nd | nd | nd | nd | + | nd | + |  | **+** |

The data of other insects are referred from *A. pisum* (Huybrechts et al. 2010), *D. melanogaster* (Hewes and Taghert 2001; Broeck 2001), *A. mellifera* (Hummon et al. 2006), *T. castaneum* (Li et al. 2008), *B. mori* (Roller et al. 2008), *N. lugens* (Tanaka et al. 2014), *R. prolixus* (Ons et al. 2011; Ons 2017), *D. citri* (Wang et al. 2018). +, identifed; nd, not identifed.
